# MicroRNA-Regulated Protein-Protein Interaction Networks and Their Functions in Breast Cancer

**DOI:** 10.3390/ijms140611560

**Published:** 2013-05-30

**Authors:** Chia-Hsien Lee, Wen-Hong Kuo, Chen-Ching Lin, Yen-Jen Oyang, Hsuan-Cheng Huang, Hsueh-Fen Juan

**Affiliations:** 1 Graduate Institute of Biomedical Electronics and Bioinformatics, National Taiwan University, Taipei 106, Taiwan; E-Mails: tfglove@gmail.com (C.-H.L.); kdragongo@gmail.com (C.-C.L.); yjoyang@csie.ntu.edu.tw (Y.-J.O.); 2 Department of Physiology, College of Medicine, National Taiwan University, Taipei 100, Taiwan; E-Mail: brcancer@gmail.com; 3 Institute of Biomedical Informatics and Center for Systems and Synthetic Biology, National Yang-Ming University, Taipei 112, Taiwan; 4 Institute of Molecular and Cellular Biology and Department of Life Science, National Taiwan University, Taipei 106, Taiwan

**Keywords:** miRNA, breast cancer, protein interaction network, functional analysis

## Abstract

MicroRNAs, which are small endogenous RNA regulators, have been associated with various types of cancer. Breast cancer is a major health threat for women worldwide. Many miRNAs were reported to be associated with the progression and carcinogenesis of breast cancer. In this study, we aimed to discover novel breast cancer-related miRNAs and to elucidate their functions. First, we identified confident miRNA-target pairs by combining data from miRNA target prediction databases and expression profiles of miRNA and mRNA. Then, miRNA-regulated protein interaction networks (PINs) were constructed with confident pairs and known interaction data in the human protein reference database (HPRD). Finally, the functions of miRNA-regulated PINs were elucidated by functional enrichment analysis. From the results, we identified some previously reported breast cancer-related miRNAs and functions of the PINs, e.g., miR-125b, miR-125a, miR-21, and miR-497. Some novel miRNAs without known association to breast cancer were also found, and the putative functions of their PINs were also elucidated. These include miR-139 and miR-383. Furthermore, we validated our results by receiver operating characteristic (ROC) curve analysis using our miRNA expression profile data, gene expression-based outcome for breast cancer online (GOBO) survival analysis, and a literature search. Our results may provide new insights for research in breast cancer-associated miRNAs.

## 1. Introduction

Breast cancer is a global health threat for women. According to a 2008 survey [1], breast cancer was the leading cause of cancer deaths in women. Our knowledge of possible risk factors has led to developments in diagnostic methods, drugs, and surgery procedures for treatment [2,3]; however, the details of breast carcinoma progression, and perhaps most importantly, how to cure breast cancer, remain elusive.

Previous research has identified a number of risk factors for breast cancer. Early menarche, late menopause, obesity, late first full pregnancy, and hormone replacement therapy were considered as high risk factors for breast cancer [2]. Breast cancer risk has also been reported to be related to fat intake in diets rich in red meats and high-fat dairy foods [3].

MicroRNAs (miRNAs) [4], are short endogenous non-coding RNAs which are able to regulate gene expression. After miRNA precursors are transcribed from the genome or generated from spliceosomes, they are exported to the cytoplasm and further processed by the Dicer complex [5]. The mature miRNA is then bound to Argonaute protein, forming a miRNA-protein complex known as the RNA-induced silencing complex (RISC) [6], miRNP, or RNAi (RNA interference) enzyme complex [7,8]. The RISC has been reported to down-regulate target genes by translational repression [9] or mRNA cleavage [10].

Like other protein-based regulators, miRNAs have been associated with cancer. Calin *et al.*, reported that miR-15 and miR-16 were deleted in leukemia [11], which was believed to be one of the earliest reports associating miRNAs with cancer [12]. After this report, many miRNAs were found to act as tumor suppressors or oncogenes (also known as oncomirs). For example, miR-21 was identified as an oncomir in hepatocellular cancer [13], breast cancer [14], and kidney cancer [15]. On the other hand, let-7c was found to be a tumor suppressor in prostate cancer [16], and miR-181a was reported as a tumor suppressor in glioma [17]. Further, miR-125b [18], and miR-145 [19] were identified as tumor suppressors in breast cancer, and miR-125a was found to repress tumor growth in breast cancer [20]. Thus, it is highly likely that miRNAs play an important role in breast cancer.

Since miRNA functions by regulating its target genes, we may deduce the effects of miRNAs by analyzing their regulated networks. To use such a method to elucidate miRNA functions, targets of miRNAs should be deduced. Currently, predictions in most target prediction database are based on sequence and statistical methods [21]. For example, in TargetScan, seed base pairing, target site context, conservation of target site and miRNA, and site accessibility are considered in the prediction process [22].

Another method to elucidate miRNA targets is to integrate expression profiles of miRNA and mRNA. In the work of Huang *et al.* [23], a Bayesian-based algorithm, GenMiR++, was developed to predict possible targets of 104 miRNAs in humans. They also verified their results by RT-PCR and microarray experiments. However, the power of other sequence-based target prediction algorithms was not utilized in their work.

It is also possible to combine sequence-based target prediction and expression-based target prediction methods. By integrating expression data into sequence-based predictions, possible false positives can be reduced. Previously, miRNA-mRNA interactions were explored with splitting-averaging Bayesian networks [24]. In that work, expression profiles of miRNA and mRNA from public databases, miRNA target prediction databases, and miRNA sequence information were integrated together to discover miRNA-mRNA interaction networks.

**Figure 1:**
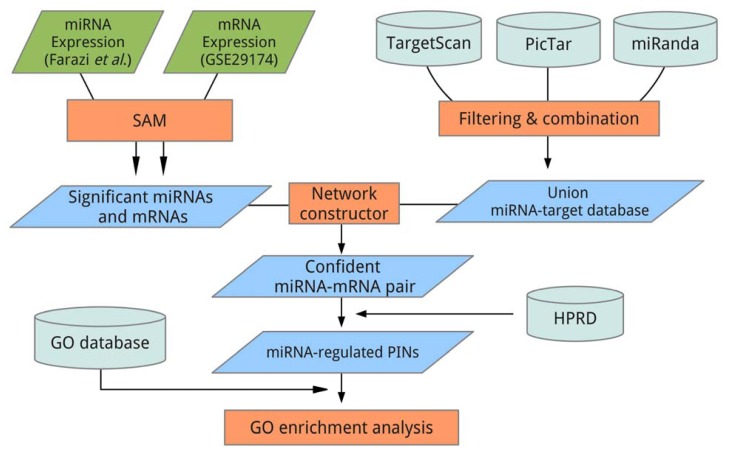
Analysis flow chart used in this work. After expression profiles and target prediction databases were fetched and preprocessed, they were subjected to the analysis process described here and in the “Experimental Section”.

Here, we combined expression profiles of miRNA and mRNA, and three target prediction databases, TargetScan, PicTar and miRanda, to obtain confident miRNA-mRNA relationships and construct miRNA-regulated protein-protein networks for breast cancer. Furthermore, we explored the functions of the miRNAs by inspecting the underlying protein interaction networks (PINs) of the miRNAs with functional enrichment analysis. This method, as described in Figure 1, was used to elucidate the functions of gastric cancer-related miRNAs in our previous work [25]. In that study, a gastric cancer-associated miRNA, miR-148a, was identified and validated as being involved in tumor proliferation, invasion, migration, and the survival rate of the patients. By using a similar method, we aimed to elucidate breast cancer-related miRNA-regulated PINs and their functions.

## 2. Results and Discussion

To construct miRNA-regulated PINs, differentially expressed miRNAs and genes from the dataset from Farazi *et al.* [26] were extracted following proper processing of the expression profiles. From our selected public miRNA dataset, we found 89 down-regulated miRNAs (93 prior to fold-change filtering) and only 1 up-regulated miRNA (Table S1). In gene expression dataset GSE29174, we found a total of 1268 down-regulated genes and 587 up-regulated genes before applying the fold change filter. There were 726 down-regulated genes (Table S2) and 437 up-regulated genes (Table S3) after significantly and differentially expressed genes were filtered by fold change (fold change >2).

From the results of SAM analysis, we identified some well-known breast cancer-related miRNAs (Table S1). For example, miR-214-3p [27] and miR-335-5p [28] have been previously reported to be down-regulated in breast cancer. Let-7c was found to be down-regulated in this work, while let-7a, another member of the let-7 family, was found to be down-regulated in another work [29]. MicroRNAs of the let-7 family were also reportedly down-regulated in several types of cancer [30]. We also found that miR-21-5p, the sole up-regulated miRNA in our list, was also previously found to be up-regulated [14,31]. However, changes in the expression of most of the miRNAs in our down-regulated list have not been reported in the literature. Therefore, we could not rule out the possibility that these miRNAs were novel breast cancer-related miRNAs. There are also some well-known miRNAs not presented in our list (for such a list, one may see [32–34]). The reason that some known miRNAs, for example, miR-19a, miR-155 and miR-205, did not show up in our result might be that we used a very stringent threshold (described in Experimental Section) when selecting differentially expressed miRNAs for PIN construction.

**Figure 2:**
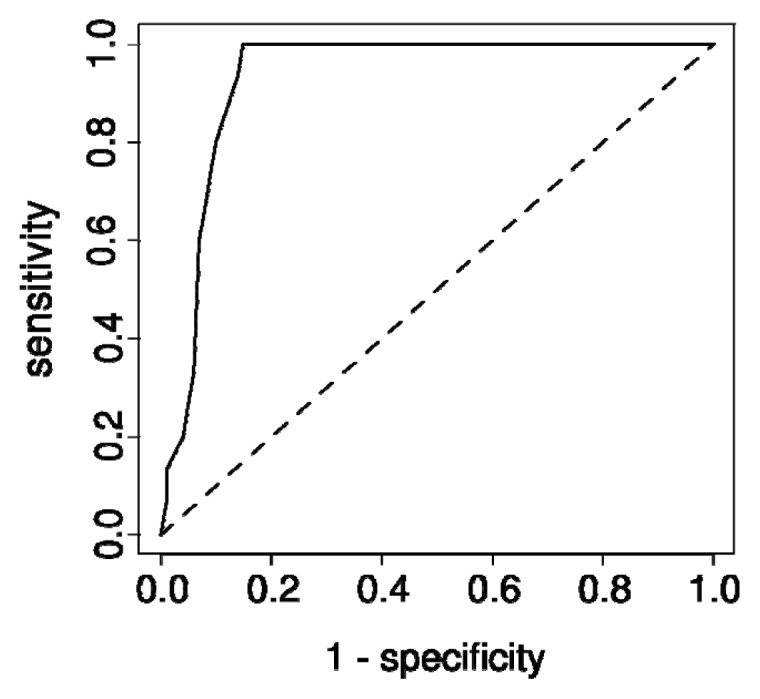
Receiver operating characteristic (ROC) curve of let-7c from our miRNA array dataset. For ROC curves of other miRNAs, see Figure S1.

Since the miRNAs of the miRNA-regulated PINs were differentially expressed between normal and tumor tissues, and we identified some cancer-related functions in our functional enrichment analysis, the miRNAs may potentially be useful diagnostic markers for breast cancer. To verify this, we applied ROC curve analysis on the miRNA expression profile that was not used in constructing the miRNA-regulated PINs. Notably, our results (Figures 2 and S1 and Table S4) showed that let-7c (Figure 2), miR-497-5p, miR-125b-5p, and some other miRNAs of miRNA-regulated PINs, performed well when used as breast cancer diagnostic markers.

Following elucidation of differentially expressed miRNAs and genes, miRNA-regulated PINs could then be constructed. We identified and constructed partial networks, containing the miRNA and its direct target, with the differentially expressed miRNAs and genes as described in the “Experimental Section”. We then extended the network by appending known interactions from the HPRD database. Finally, 18 miRNA-regulated PINs were constructed by the steps described above (Figure 3, Figures S2–S13, and Table S5).

After construction of the 18 PINs was completed, we observed that the sizes of the PINs were not similar: some of the miRNAs seemed to regulate larger sized PINs, while other miRNAs affected only a small number of genes. Small miRNA-regulated PINs may be caused by the strict *q*-value threshold set during SAM analysis, the processing steps performed on the target prediction databases discussed previously, and possibly by lack of protein-protein interaction data for some proteins in the HPRD. Although the HPRD may be considered the most comprehensive source of protein-protein interaction data [35], some proteins may not have been considered and researched by other investigators, and therefore, interaction data for those proteins would not be included in the HPRD. However, it may be true that some of the miRNA-regulated PINs were small in breast cancer, since the construction of the PINs were based on differentially expressed miRNAs and genes between normal tissues and tumor samples, and those miRNAs with small PINs may not be as important as others with larger PINs.

To elucidate the functions of a miRNA with a regulated PIN, GO enrichment analysis was applied to the miRNA-regulated PINs. We did not consider the miRNAs with ≤5 genes in their regulated PINs, and some of the miRNA-regulated PINs had no enriched functions using the defined threshold, FDR < 0.0001. To exclude GO terms that describe a broad range of concepts, we only included high level GO terms, *i.e.*, larger than 5.

The results of the GO enrichment analysis for let-7c related to cancer are listed in Table 1. (Results of all miRNA-regulated PINs were in Table S6). We defined a GO term as cancer-related if a GO term contained “cell proliferation”, “cell death”, “apoptosis”, “signaling”, “microtubule”, and “actin”. We noted that 7 miRNAs had enriched GO terms related to apoptosis, cell death, and cell proliferation, *i.e.*, miR-520d-3p, miR-497-5p, miR-125b-5p, miR-21-5p, miR-31-5p, let-7c, and miR-125-5p. Further, some miRNA-regulated PINs may have functions other than cell survival. For example, the nerve growth factor receptor pathway was enriched in miR-regulated PINs of miR-520d-3p, miR-497-5p, miR-125a-5p, miR-125b-5p, and miR-31-5p, and the epidermal growth factor receptor pathway was enriched in miR-regulated PINs of miR-520d-3p, miR-21-5p, and miR-497-5p. Most of the miRNAs had been previously described and were known to be implicated in breast cancer. Let-7c was not only likely to be down-regulated in breast cancer [29], but was also found to be a tumor suppressor in prostate cancer [16]. Another reported tumor suppressor was miR-125b-5p, which was found to be down-regulated in breast tumor tissue [18], and this finding was consistent with our functional enrichment results. The miR-125a-5p-regulated PIN was found to be able to inhibit apoptosis and regulate epithelial cell proliferation, and has been reported to repress cell growth [36].

We also found that some breast cancer-specific functions were enriched in our results. For example, in the miR-497-5p-regulated PIN, the term “androgen receptor signaling pathway” was enriched. Although it is not clear whether androgens are related to breast cancer, androgen receptors are known to be up-regulated in breast cancer and related to node invasiveness [37].

To further verify the results of the enriched cancer-related functions, we used GOBO for survival analysis. Our hypothesis is that expression of genes annotated with enriched cancer-related terms may be related to survival outcome of patients. With exception to some cell death/proliferation-related terms, it is already known that some pathways or functions are also related to clinical outcomes. For example, cell proliferation-related GO terms have a high probability of affecting survival of cancer tissues, and patient outcome may worsen if cancer tissue survives. In addition, some signaling pathways were known to enhance invasiveness, migration abilities, or were associated with reduced patient survival. For example, the BMP signaling pathway is known to confer various tumor cells with enhanced migration and invasion abilities [38], and nerve growth factor receptor (NGFR) was found to be associated with overall survival of breast cancer [38]. Furthermore, Toll-like receptor 4 has been reported to promote adhesion and invasive migration in breast cancer [39]. Finally, the cytoskeleton plays an important role in regulating cell motility in all cells. Actin filaments are known to participate in the invasive migration of cancer cells [40]. Since some of these functions were present in our enriched terms, we wished to test if the expression of a gene set annotated to the cancer-related enriched terms in the PIN would be related to clinical outcome of patients.

**Figure 4:**
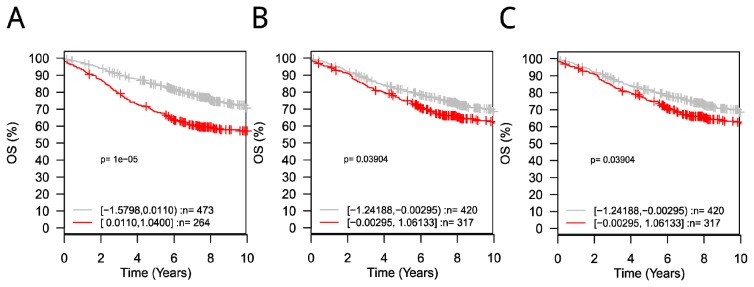
Validation of let-7c result. Gene expression-based outcome for breast cancer online (GOBO) survival analysis of let-7c-regulated PIN members marked with the following functions: (**A**) Microtubule cytoskeleton; (**B**) Negative regulation of programmed cell death; and (**C**) negative regulation of cell death. **Red**: samples with high expression of selected gene set (PIN members); **grey**: samples with low expression of selected gene set (PIN members).

As shown in Figure 4, Figure S14 and Table S7, only some of the enriched terms were significantly associated with clinical outcome. This may be because the changes in these key genes occurred at the protein level, such as in protein expression or even post-translational modification; therefore, mRNA expression-centric tools like GOBO cannot explore association of such genes to clinical outcomes. Alternatively, it is possible the miRNA did not regulate the whole pathway, or the miRNA did not target the key part of the pathway directly, and thus the clinical outcome of gene sets of the enriched GO terms in this condition cannot be determined. However, some functions associated with clinical outcomes were observed. For example, proteins annotated with the terms “microtubule cytoskeleton”, “negative regulation of programmed cell death”, and “negative regulation of cell death” in the let-7c-regulated PIN were related to 10 year survival rate of patients, as reported by GOBO (Figure 4). Also, the enriched term “regulation of epithelial cell proliferation” for both miR-125a-5p and miR-125b-5p were found to be associated with the 10-year survival rate of patients. Therefore, these results further supported the GO enrichment analysis discussed previously.

## 3. Experimental Section

### 3.1. miRNA Microarray Experiments

We performed a miRNA microarray to obtain the expression profiles for receiver operating characteristic (ROC) curve analysis. This dataset was deposited in Gene Expression Omnibus (GEO, http://www.ncbi.nlm.nih.gov/geo/, Series Accession: GSE45666). In total, there were 15 normal samples and 101 tumor samples in the expression profile. Detailed pathophysiogical characteistics of these samples were in Table S8 and GSE45666. All human tissue samples collected from breast cancer patients were approved and human subject confidentiality was protected by the Institute Review Board of National Taiwan University Hospital (IRB, 20071211R).

Total RNA was extracted from tissues collected from the patients using Trizol^®^ Reagent (Invitrogen, Carlsbad, CA, USA, USA) according to the manufacturer’s protocol. Purified RNA was quantified at OD260nm by using a ND-1000 spectrophotometer (Nanodrop Technology, Wilmington, DE, USA) and qualitated by using a Bioanalyzer 2100 with the RNA 6000 Nano LabChip kit (Agilent Technologies, Santa Clara, CA, USA).

After RNA extraction, 100 ng of total RNA was dephosphorylated and labeled with pCp-Cy3 using the Agilent miRNA Complete Labeling and Hyb Kit in conjunction with the microRNA Spike-In kit (Agilent Technologies, Santa Clara, CA, USA). Briefly, 2X hybridization buffer (Agilent Technologies) was added to the labeled mixture to a final volume of 45 μL. The mixture was heated for 5 min at 100 °C and immediately cooled to 0 °C. Each 45 μL sample was hybridized onto an Agilent human miRNA Microarray Release 12.0, 8 × 15 K (Agilent Technologies) at 55 °C for 20 h. After hybridization, slides were washed for 5 min in Gene Expression Wash Buffer 1 at room temperature, then for 5 min in Gene Expression Wash Buffer 2 at 37 °C. Slides were scanned on an Agilent microarray scanner (Agilent Technologies, model G2505C) at 100% and 5% sensitivity settings. Feature Extraction (Agilent Technologies) software version 10.7.3.1 (Agilent Technologies, Santa Clara, CA, USA) was used for image analysis.

### 3.2. mRNA Expression Profiles

For miRNA-regulated protein-protein interaction network construction, the mRNA expression profile was fetched from GEO (Series Accession: GSE29174). This dataset was produced by Farazi *et al*. [26], which was the only dataset publicly available with large size of tumor samples and reasonably sized normal samples. In total, 161 clinical samples were collected from breast cancer patients by biopsy: 110 invasive ductal carcinoma (IDC), 11 normal, 17 ductal carcinoma *in situ* (DCIS), 1 mucinous A, 8 atypical medullary, 4 apocrine, 8 metaplastic, and 2 adenoid, as classified by Farazi *et al.* The 110 IDC samples were classified as the tumor group and the 11 normal samples were classified as the normal group in this study.

### 3.3. miRNA Expression Profiles

The miRNA expression profile used in this work was fetched from Table S4A of the work of Farazi *et al*. [26]. There were 189 samples in this dataset, with 6 of them from cell lines, and another 183 samples from patient tissues. In the 183 clinical samples collected from breast cancer patients by biopsy in this dataset, there were 128 IDC, 11 normal, 18 DCIS, 1 mucinous A, 8 atypical medullary, 4 apocrine, 9 metaplastic, and 2 adenoid cases, as classified by Farazi *et al.* The 128 IDC samples were classified as the tumor group and the 11 normal samples as were classified as the normal group.

### 3.4. Data Analysis

The overall workflow design was similar to the previous work of Tseng *et al*. [25] (see Figure 1). However, we applied the workflow on breast cancer expression profiles instead of gastric cancer as in the work of Tseng *et al.* Additionally, we used 3 miRNA target prediction databases, TargetScan (v6.0) [22,41,42], PicTar [43,44], and miRanda (release August 2010) [45] here, while only TargetScan was used in the previous study.

To construct the networks, we first elucidated differentially expressed miRNAs and mRNAs from published datasets. Both of miRNA (generated with miRNA-Seq technique) and mRNA array expression data were produced by Farazi *et al.* [26] from the same batch of clinical tumor samples. To obtain a list of differentially expressed genes and miRNAs between normal and tumor groups, we used significance analysis of microarrays (SAM) [46] implemented in R package samr (version 2.0; Stanford University, Stanford, CA, USA). We set the false discovery rate (FDR) as ≤0.0001%, fold change as ≥2.5 for miRNA, and fold change as ≥1.9 for genes as thresholds to reduce false positives. If a gene/miRNA was expressed higher in the normal group compared to the tumor group, we defined that gene/miRNA as a down-regulated gene/miRNA, and *vice versa*.

Following this, we paired the miRNAs and mRNAs with different expression trends. For example, an up-regulated miRNA would be paired with a down-regulated mRNA. If such pairs could be found in 2 (or more) of the 3 miRNA target prediction databases, they were then added to their corresponding miRNA-regulated network. To further extend the coverage of our network, we incorporated the human protein reference database (HPRD) [47], which contains experimentally verified interaction data, into our miRNA-regulated PINs.

Finally, we used gene ontology (GO) enrichment analysis to explore the function of the miRNA-regulated PINs. Hypergeometric tests were used to determine if a GO term was enriched in a PIN. In this section, we excluded PINs with less than 5 proteins. We also excluded GO terms with levels of less than 5 to avoid non-specific GO terms. Since we tested multiple GO terms on each miRNA-regulated PIN, we adjusted the significance of the test with the FDR method developed by Benjamini *et al.* [48]. We used the adjusted *p*-value < 0.0001 as our threshold. A GO term would be excluded if its *p*-value was larger than 0.0001. Therefore, for each network, we ran the GO enrichment analysis, collected the calculated *p*-values, and adjusted these values using the methods described above.

### 3.5. ROC and GOBO Survival Analysis

After the PINs were constructed, we attempted to verify our results by literature search, ROC, and GOBO survival analysis [49]. To determine if the miRNAs we found could serve as classification markers for discriminating between normal and tumor samples, we applied ROC analysis on our miRNA array data (described in Section 2.1). ROC analysis is usually used to evaluate the efficiency of a classifier or a biological marker. R package ROCR [50] (version 1.0.4) was used to plot the ROC curve and calculate the area under curve (AUC). The standard error of AUC was then calculated as described in the work of Hanlye and McNeil [51]. The *p*-value of AUC was thus calculated with standard error obtained in the previous step. To further validate if the PINs we found were related to cancer, we used survival analysis implemented in GOBO [49] (available at http://co.bmc.lu.se/gobo), which provides a large amount of breast cancer gene expression profiles collected from public databases with clinical outcome data. In both ROC analysis and GOBO survival analysis, we considered our results significant when the *p*-value was smaller than 0.05.

## 4. Conclusions

Using integrative analysis of miRNA and mRNA expression profiles, we have identified not only breast cancer-related miRNAs and genes, but also putative roles for miRNAs in cancer as elucidated from miRNA-regulated PINs constructed in this work. Here, some previously known functions of miRNAs were again presented in our results, e.g., the relationship between the miRNAs, let-7c, miR-125a-5p, miR-125b-5p, and miR-21-5p, and breast cancer were demonstrated in this research. Furthermore, we have identified additional miRNAs and their related functions that have not been previously reported or discussed, providing valuable resources for further research in breast cancer.

## Supplementary Information

**Table S1:** Significantly differentially expressed miRNAs found in miRNA dataset in Farazi *et al*. [26]. There are 89 down-regulated miRNAs and 1 up-regulated miRNA in this list. *Q*-values reported by SAM were 0 for all miRNAs in this list.

miRBase Accession	miRNA Name	Fold Change
**MIMAT0004761**	hsa-miR-483-5p	0.01
**MIMAT0004552**	hsa-miR-139-3p	0.01
**MIMAT0000738**	hsa-miR-383	0.02
**MIMAT0002856**	hsa-miR-520d-3p	0.02
**MIMAT0002811**	hsa-miR-202-3p	0.03
**MIMAT0002177**	hsa-miR-486-5p	0.04
**MIMAT0022721**	hsa-miR-1247-3p	0.05
**MIMAT0002175**	hsa-miR-485-5p	0.06
**MIMAT0000265**	hsa-miR-204-5p	0.07
**MIMAT0000752**	hsa-miR-328	0.07
**MIMAT0000421**	hsa-miR-122-5p	0.07
**MIMAT0000447**	hsa-miR-134	0.08
**MIMAT0000722**	hsa-miR-370	0.09
**MIMAT0004513**	hsa-miR-101-5p	0.09
**MIMAT0000446**	hsa-miR-127-3p	0.10
**MIMAT0000097**	hsa-miR-99a-5p	0.10
**MIMAT0004566**	hsa-miR-218-2-3p	0.10
**MIMAT0000729**	hsa-miR-376a-3p	0.11
**MIMAT0009197**	hsa-miR-205-3p	0.11
**MIMAT0004615**	hsa-miR-195-3p	0.11
**MIMAT0005899**	hsa-miR-1247-5p	0.11
**MIMAT0000720**	hsa-miR-376c	0.12
**MIMAT0000762**	hsa-miR-324-3p	0.12
**MIMAT0004679**	hsa-miR-296-3p	0.12
**MIMAT0004614**	hsa-miR-193a-5p	0.12
**MIMAT0003880**	hsa-miR-671-5p	0.12
**MIMAT0004795**	hsa-miR-574-5p	0.12
**MIMAT0004599**	hsa-miR-143-5p	0.13
**MIMAT0000423**	hsa-miR-125b-5p	0.13
**MIMAT0004957**	hsa-miR-760	0.13
**MIMAT0004911**	hsa-miR-874	0.14
**MIMAT0004603**	hsa-miR-125b-2-3p	0.15
**MIMAT0004952**	hsa-miR-665	0.15
**MIMAT0018205**	hsa-miR-3928	0.15
**MIMAT0004767**	hsa-miR-193b-5p	0.15
**MIMAT0002861**	hsa-miR-518e-3p	0.15
**MIMAT0004604**	hsa-miR-127-5p	0.16
**MIMAT0002807**	hsa-miR-491-5p	0.16
**MIMAT0004689**	hsa-miR-377-5p	0.16
**MIMAT0004762**	hsa-miR-486-3p	0.16
**MIMAT0000732**	hsa-miR-378a-3p	0.17
**MIMAT0017981**	hsa-miR-3605-5p	0.18
**MIMAT0004605**	hsa-miR-129-2-3p	0.19
**MIMAT0006789**	hsa-miR-1468	0.20
**MIMAT0000737**	hsa-miR-382-5p	0.21
**MIMAT0000077**	hsa-miR-22-3p	0.21
**MIMAT0000089**	hsa-miR-31-5p	0.21
**MIMAT0004612**	hsa-miR-186-3p	0.21
**MIMAT0004592**	hsa-miR-125b-1-3p	0.22
**MIMAT0001639**	hsa-miR-409-3p	0.22
**MIMAT0015032**	hsa-miR-3158-3p	0.22
**MIMAT0004496**	hsa-miR-23a-5p	0.22
**MIMAT0000690**	hsa-miR-296-5p	0.22
**MIMAT0000731**	hsa-miR-378a-5p	0.23
**MIMAT0000448**	hsa-miR-136-5p	0.23
**MIMAT0004796**	hsa-miR-576-3p	0.23
**MIMAT0010133**	hsa-miR-2110	0.23
**MIMAT0004951**	hsa-miR-887	0.23
**MIMAT0003239**	hsa-miR-574-3p	0.25
**MIMAT0005901**	hsa-miR-1249	0.25
**MIMAT0000510**	hsa-miR-320a	0.26
**MIMAT0002172**	hsa-miR-376b	0.26
**MIMAT0000250**	hsa-miR-139-5p	0.27
**MIMAT0005825**	hsa-miR-1180	0.27
**MIMAT0000437**	hsa-miR-145-5p	0.28
**MIMAT0004601**	hsa-miR-145-3p	0.28
**MIMAT0003322**	hsa-miR-652-3p	0.28
**MIMAT0000756**	hsa-miR-326	0.28
**MIMAT0000098**	hsa-miR-100-5p	0.29
**MIMAT0003296**	hsa-miR-627	0.29
**MIMAT0002820**	hsa-miR-497-5p	0.31
**MIMAT0004507**	hsa-miR-92a-1-5p	0.31
**MIMAT0000271**	hsa-miR-214-3p	0.32
**MIMAT0004702**	hsa-miR-339-3p	0.33
**MIMAT0004611**	hsa-miR-185-3p	0.33
**MIMAT0000064**	hsa-let-7c	0.34
**MIMAT0004673**	hsa-miR-29c-5p	0.35
**MIMAT0000733**	hsa-miR-379-5p	0.35
**MIMAT0004594**	hsa-miR-132-5p	0.35
**MIMAT0000765**	hsa-miR-335-5p	0.35
**MIMAT0002819**	hsa-miR-193b-3p	0.36
**MIMAT0000088**	hsa-miR-30a-3p	0.36
**MIMAT0005951**	hsa-miR-1307-3p	0.36
**MIMAT0004597**	hsa-miR-140-3p	0.37
**MIMAT0004556**	hsa-miR-10b-3p	0.37
**MIMAT0000272**	hsa-miR-215	0.37
**MIMAT0004511**	hsa-miR-99a-3p	0.37
**MIMAT0000443**	hsa-miR-125a-5p	0.38
**MIMAT0004482**	hsa-let-7b-3p	0.38
**MIMAT0000076**	hsa-miR-21-5p	6.58

**Table S2:** Down-regulated genes found in dataset GSE29174. There are 726 down-regulated genes in this list. *Q*-values reported by SAM were 0 for all genes in this list.

NCBI gene ID	Gene Symbol	Fold Change
**2949**	GSTM5	0.06
**10894**	LYVE1	0.06
**5950**	RBP4	0.07
**762**	CA4	0.09
**54997**	TESC	0.09
**3489**	IGFBP6	0.09
**3952**	LEP	0.09
**213**	ALB	0.09
**3131**	HLF	0.10
**4023**	LPL	0.10
**10633**	RASL10A	0.11
**364**	AQP7	0.11
**1908**	EDN3	0.11
**1811**	SLC26A3	0.11
**91851**	CHRDL1	0.11
**729359**	PLIN4	0.13
**1149**	CIDEA	0.13
**5959**	RDH5	0.13
**5348**	FXYD1	0.14
**5346**	PLIN1	0.14
**10249**	GLYAT	0.14
**158800**	RHOXF1	0.14
**221476**	PI16	0.14
**3040**	HBA2	0.14
**6939**	TCF15	0.14
**79645**	EFCAB1	0.14
**80343**	SEL1L2	0.14
**9413**	FAM189A2	0.15
**26289**	AK5	0.15
**25891**	PAMR1	0.15
**3679**	ITGA7	0.15
**1264**	CNN1	0.15
**92304**	SCGB3A1	0.15
**2167**	FABP4	0.15
**23285**	KIAA1107	0.15
**7145**	TNS1	0.16
**4881**	NPR1	0.16
**1028**	CDKN1C	0.16
**1036**	CDO1	0.16
**130271**	PLEKHH2	0.16
**8736**	MYOM1	0.16
**8908**	GYG2	0.16
**619373**	MBOAT4	0.17
**130399**	ACVR1C	0.17
**1646**	AKR1C2	0.17
**80763**	C12orf39	0.17
**2159**	F10	0.18
**84889**	SLC7A3	0.18
**1308**	COL17A1	0.18
**83699**	SH3BGRL2	0.18
**84417**	C2orf40	0.18
**4081**	MAB21L1	0.18
**3484**	IGFBP1	0.18
**5239**	PGM5	0.19
**4969**	OGN	0.19
**2719**	GPC3	0.19
**116362**	RBP7	0.19
**948**	CD36	0.19
**5764**	PTN	0.19
**3043**	HBB	0.19
**56920**	SEMA3G	0.20
**94274**	PPP1R14A	0.20
**57447**	NDRG2	0.20
**84795**	PYROXD2	0.20
**84649**	DGAT2	0.20
**2690**	GHR	0.20
**22802**	CLCA4	0.20
**5179**	PENK	0.20
**6663**	SOX10	0.20
**6649**	SOD3	0.21
**54922**	RASIP1	0.21
**8406**	SRPX	0.21
**1446**	CSN1S1	0.21
**7123**	CLEC3B	0.22
**9647**	PPM1F	0.22
**1842**	ECM2	0.22
**3909**	LAMA3	0.22
**8639**	AOC3	0.23
**2934**	GSN	0.23
**9370**	ADIPOQ	0.23
**3202**	HOXA5	0.23
**9452**	ITM2A	0.23
**6290**	SAA3P	0.23
**4604**	MYBPC1	0.23
**79785**	RERGL	0.16
**221091**	LRRN4CL	0.17
**3991**	LIPE	0.17
**27175**	TUBG2	0.24
**1346**	COX7A1	0.24
**6376**	CX3CL1	0.24
**50486**	G0S2	0.24
**6285**	S100B	0.24
**443**	ASPA	0.24
**947**	CD34	0.25
**84632**	AFAP1L2	0.25
**3866**	KRT15	0.25
**147463**	ANKRD29	0.25
**2878**	GPX3	0.25
**7079**	TIMP4	0.25
**54345**	SOX18	0.25
**51277**	DNAJC27	0.25
**84870**	RSPO3	0.25
**55323**	LARP6	0.25
**6387**	CXCL12	0.25
**137835**	TMEM71	0.25
**5212**	VIT	0.25
**26577**	PCOLCE2	0.25
**845**	CASQ2	0.25
**6422**	SFRP1	0.25
**10351**	ABCA8	0.26
**10840**	ALDH1L1	0.26
**65983**	GRAMD3	0.26
**84327**	ZBED3	0.26
**57124**	CD248	0.26
**3235**	HOXD9	0.26
**2192**	FBLN1	0.26
**91653**	BOC	0.26
**4147**	MATN2	0.26
**126669**	SHE	0.27
**2788**	GNG7	0.27
**129804**	FBLN7	0.27
**270**	AMPD1	0.27
**79656**	BEND5	0.27
**58503**	PROL1	0.27
**3316**	HSPB2	0.27
**729440**	CCDC61	0.27
**54438**	GFOD1	0.27
**5243**	ABCB1	0.27
**1128**	CHRM1	0.23
**83878**	USHBP1	0.24
**63970**	TP53AIP1	0.24
**79192**	IRX1	0.28
**3400**	ID4	0.28
**57519**	STARD9	0.29
**57666**	FBRSL1	0.29
**3590**	IL11RA	0.29
**57664**	PLEKHA4	0.29
**197257**	LDHD	0.29
**66036**	MTMR9	0.29
**2321**	FLT1	0.29
**126**	ADH1C	0.29
**1363**	CPE	0.29
**56131**	PCDHB4	0.29
**22915**	MMRN1	0.29
**7069**	THRSP	0.29
**57161**	PELI2	0.30
**770**	CA11	0.30
**53342**	IL17D	0.30
**79987**	SVEP1	0.30
**857**	CAV1	0.30
**222166**	C7orf41	0.30
**27190**	IL17B	0.30
**116159**	CYYR1	0.30
**4487**	MSX1	0.30
**9068**	ANGPTL1	0.30
**10411**	RAPGEF3	0.30
**3199**	HOXA2	0.30
**2944**	GSTM1	0.30
**2920**	CXCL2	0.30
**201134**	CEP112	0.31
**220001**	VWCE	0.31
**83888**	FGFBP2	0.31
**6366**	CCL21	0.31
**6711**	SPTBN1	0.31
**85378**	TUBGCP6	0.31
**26040**	SETBP1	0.31
**4692**	NDN	0.31
**25890**	ABI3BP	0.31
**23531**	MMD	0.31
**30846**	EHD2	0.31
**6196**	RPS6KA2	0.31
**2009**	EML1	0.31
**810**	CALML3	0.27
**6898**	TAT	0.27
**5648**	MASP1	0.28
**25999**	CLIP3	0.28
**125875**	CLDND2	0.28
**7102**	TSPAN7	0.28
**1879**	EBF1	0.28
**23252**	OTUD3	0.28
**5493**	PPL	0.28
**83987**	CCDC8	0.28
**9073**	CLDN8	0.28
**221981**	THSD7A	0.28
**64102**	TNMD	0.28
**137872**	ADHFE1	0.33
**27151**	CPAMD8	0.33
**387923**	SERP2	0.33
**145581**	LRFN5	0.33
**6263**	RYR3	0.33
**2354**	FOSB	0.33
**51302**	CYP39A1	0.33
**4128**	MAOA	0.34
**117248**	GALNTL2	0.34
**10268**	RAMP3	0.34
**7730**	ZNF177	0.34
**10873**	ME3	0.34
**7461**	CLIP2	0.34
**7049**	TGFBR3	0.34
**79901**	CYBRD1	0.34
**5152**	PDE9A	0.34
**50805**	IRX4	0.34
**8644**	AKR1C3	0.34
**5915**	RARB	0.34
**2770**	GNAI1	0.34
**54996**	2-Mar	0.35
**79791**	FBXO31	0.35
**54776**	PPP1R12C	0.35
**9079**	LDB2	0.35
**57104**	PNPLA2	0.35
**30008**	EFEMP2	0.35
**91461**	PKDCC	0.35
**23368**	PPP1R13B	0.35
**23461**	ABCA5	0.35
**9572**	NR1D1	0.35
**23338**	PHF15	0.35
**6289**	SAA2	0.31
**345275**	HSD17B13	0.31
**2701**	GJA4	0.32
**112609**	MRAP2	0.32
**727**	C5	0.32
**477**	ATP1A2	0.32
**9627**	SNCAIP	0.32
**4435**	CITED1	0.32
**10974**	C10orf116	0.32
**11005**	SPINK5	0.32
**80325**	ABTB1	0.33
**221395**	GPR116	0.33
**10014**	HDAC5	0.33
**1489**	CTF1	0.37
**35**	ACADS	0.37
**3749**	KCNC4	0.37
**140738**	TMEM37	0.37
**2791**	GNG11	0.37
**23604**	DAPK2	0.37
**10217**	CTDSPL	0.37
**23550**	PSD4	0.37
**4306**	NR3C2	0.37
**119587**	CPXM2	0.37
**7942**	TFEB	0.37
**3815**	KIT	0.37
**1805**	DPT	0.37
**23242**	COBL	0.37
**4313**	MMP2	0.37
**4139**	MARK1	0.37
**9104**	RGN	0.37
**2329**	FMO4	0.37
**25802**	LMOD1	0.38
**4239**	MFAP4	0.38
**10392**	NOD1	0.38
**6794**	STK11	0.38
**85458**	DIXDC1	0.38
**4123**	MAN2C1	0.38
**54476**	RNF216	0.38
**9920**	KBTBD11	0.38
**6329**	SCN4A	0.38
**10253**	SPRY2	0.38
**1910**	EDNRB	0.38
**9249**	DHRS3	0.38
**22869**	ZNF510	0.38
**114800**	CCDC85A	0.35
**2550**	GABBR1	0.35
**4638**	MYLK	0.35
**2327**	FMO2	0.35
**139411**	PTCHD1	0.35
**10391**	CORO2B	0.35
**25854**	FAM149A	0.35
**55701**	ARHGEF40	0.36
**1759**	DNM1	0.36
**22849**	CPEB3	0.36
**57716**	PRX	0.36
**1628**	DBP	0.36
**80031**	SEMA6D	0.36
**259217**	HSPA12A	0.36
**6909**	TBX2	0.36
**1511**	CTSG	0.36
**79971**	WLS	0.36
**90865**	IL33	0.36
**11343**	MGLL	0.36
**55800**	SCN3B	0.36
**1949**	EFNB3	0.36
**284217**	LAMA1	0.36
**22927**	HABP4	0.37
**23645**	PPP1R15A	0.39
**342574**	KRT27	0.39
**83543**	AIF1L	0.39
**624**	BDKRB2	0.39
**347**	APOD	0.39
**84935**	C13orf33	0.39
**858**	CAV2	0.39
**5138**	PDE2A	0.40
**114928**	GPRASP2	0.40
**58190**	CTDSP1	0.40
**513**	ATP5D	0.40
**57684**	ZBTB26	0.40
**7041**	TGFB1I1	0.40
**5787**	PTPRB	0.40
**7294**	TXK	0.40
**56301**	SLC7A10	0.40
**55937**	APOM	0.40
**6368**	CCL23	0.40
**55020**	TTC38	0.40
**134265**	AFAP1L1	0.40
**4485**	MST1	0.40
**3384**	ICAM2	0.38
**8613**	PPAP2B	0.38
**1950**	EGF	0.38
**55273**	TMEM100	0.38
**6297**	SALL2	0.38
**9365**	KL	0.38
**8863**	PER3	0.38
**8404**	SPARCL1	0.38
**2202**	EFEMP1	0.38
**8369**	HIST1H4G	0.38
**5187**	PER1	0.39
**30815**	ST6GALNAC6	0.39
**256364**	EML3	0.39
**57381**	RHOJ	0.39
**761**	CA3	0.39
**83989**	FAM172A	0.39
**1408**	CRY2	0.39
**2281**	FKBP1B	0.39
**51222**	ZNF219	0.39
**54540**	FAM193B	0.39
**4053**	LTBP2	0.39
**55184**	DZANK1	0.39
**5740**	PTGIS	0.39
**84814**	PPAPDC3	0.42
**79365**	BHLHE41	0.42
**316**	AOX1	0.42
**23380**	SRGAP2	0.42
**84033**	OBSCN	0.42
**90353**	CTU1	0.42
**9013**	TAF1C	0.42
**474344**	GIMAP6	0.42
**84883**	AIFM2	0.42
**58480**	RHOU	0.42
**65982**	ZSCAN18	0.42
**666**	BOK	0.42
**79762**	C1orf115	0.42
**525**	ATP6V1B1	0.42
**4675**	NAP1L3	0.42
**3257**	HPS1	0.43
**55781**	RIOK2	0.43
**63947**	DMRTC1	0.43
**1969**	EPHA2	0.43
**25927**	CNRIP1	0.43
**57685**	CACHD1	0.43
**51559**	NT5DC3	0.40
**7169**	TPM2	0.40
**51705**	EMCN	0.40
**8938**	BAIAP3	0.40
**10365**	KLF2	0.40
**59**	ACTA2	0.40
**80309**	SPHKAP	0.40
**3779**	KCNMB1	0.41
**10826**	C5orf4	0.41
**219654**	ZCCHC24	0.41
**92162**	TMEM88	0.41
**7450**	VWF	0.41
**10266**	RAMP2	0.41
**25875**	LETMD1	0.41
**1938**	EEF2	0.41
**121551**	BTBD11	0.41
**2119**	ETV5	0.41
**9696**	CROCC	0.41
**1031**	CDKN2C	0.41
**9037**	SEMA5A	0.41
**3397**	ID1	0.41
**84707**	BEX2	0.41
**57616**	TSHZ3	0.41
**1471**	CST3	0.41
**55214**	LEPREL1	0.41
**3914**	LAMB3	0.41
**57478**	USP31	0.41
**3783**	KCNN4	0.41
**8839**	WISP2	0.41
**1583**	CYP11A1	0.42
**10124**	ARL4A	0.42
**738**	C11orf2	0.42
**29800**	ZDHHC1	0.42
**23135**	KDM6B	0.44
**171024**	SYNPO2	0.44
**10350**	ABCA9	0.44
**3691**	ITGB4	0.44
**2348**	FOLR1	0.44
**11145**	PLA2G16	0.44
**554**	AVPR2	0.45
**64072**	CDH23	0.45
**80177**	MYCT1	0.45
**5957**	RCVRN	0.45
**408**	ARRB1	0.45
**29997**	GLTSCR2	0.43
**26051**	PPP1R16B	0.43
**83604**	TMEM47	0.43
**2308**	FOXO1	0.43
**55225**	RAVER2	0.43
**54839**	LRRC49	0.43
**122953**	JDP2	0.43
**29775**	CARD10	0.43
**166**	AES	0.43
**25924**	MYRIP	0.43
**2852**	GPER	0.43
**51421**	AMOTL2	0.43
**124936**	CYB5D2	0.43
**1294**	COL7A1	0.43
**127435**	PODN	0.43
**84952**	CGNL1	0.43
**83483**	PLVAP	0.43
**1958**	EGR1	0.43
**230**	ALDOC	0.43
**65987**	KCTD14	0.43
**4804**	NGFR	0.44
**64852**	TUT1	0.44
**84253**	GARNL3	0.44
**5866**	RAB3IL1	0.44
**10608**	MXD4	0.44
**4211**	MEIS1	0.44
**83547**	RILP	0.44
**9172**	MYOM2	0.44
**57192**	MCOLN1	0.44
**255877**	BCL6B	0.44
**56904**	SH3GLB2	0.44
**51285**	RASL12	0.44
**3425**	IDUA	0.44
**402117**	VWC2L	0.46
**81490**	PTDSS2	0.46
**283748**	PLA2G4D	0.46
**23523**	CABIN1	0.46
**6146**	RPL22	0.46
**85360**	SYDE1	0.46
**60468**	BACH2	0.46
**57451**	ODZ2	0.46
**4013**	VWA5A	0.46
**339768**	ESPNL	0.46
**3860**	KRT13	0.46
**144699**	FBXL14	0.45
**83719**	YPEL3	0.45
**22841**	RAB11FIP2	0.45
**283927**	NUDT7	0.45
**293**	SLC25A6	0.45
**90507**	SCRN2	0.45
**37**	ACADVL	0.45
**112744**	IL17F	0.45
**6709**	SPTAN1	0.45
**8086**	AAAS	0.45
**7423**	VEGFB	0.45
**64221**	ROBO3	0.45
**7273**	TTN	0.45
**2657**	GDF1	0.45
**59271**	C21orf63	0.45
**132160**	PPM1M	0.45
**27244**	SESN1	0.45
**51310**	SLC22A17	0.45
**4828**	NMB	0.45
**54360**	CYTL1	0.45
**203245**	NAIF1	0.45
**23166**	STAB1	0.45
**2121**	EVC	0.45
**116496**	FAM129A	0.45
**23239**	PHLPP1	0.45
**51673**	TPPP3	0.45
**64094**	SMOC2	0.45
**6383**	SDC2	0.45
**2180**	ACSL1	0.45
**23770**	FKBP8	0.45
**55901**	THSD1	0.46
**25895**	METTL21B	0.46
**23731**	C9orf5	0.46
**126393**	HSPB6	0.46
**4056**	LTC4S	0.46
**79825**	CCDC48	0.46
**10810**	WASF3	0.46
**29911**	HOOK2	0.46
**583**	BBS2	0.46
**28984**	C13orf15	0.46
**1465**	CSRP1	0.46
**55258**	THNSL2	0.46
**161198**	CLEC14A	0.46
**3699**	ITIH3	0.48
**7094**	TLN1	0.46
**4232**	MEST	0.46
**1410**	CRYAB	0.46
**57452**	GALNTL1	0.47
**63935**	PCIF1	0.47
**25873**	RPL36	0.47
**9812**	KIAA0141	0.47
**51665**	ASB1	0.47
**64123**	ELTD1	0.47
**6122**	RPL3	0.47
**222962**	SLC29A4	0.47
**23102**	TBC1D2B	0.47
**3476**	IGBP1	0.47
**93408**	MYL10	0.47
**5310**	PKD1	0.47
**4628**	MYH10	0.47
**221935**	SDK1	0.47
**23328**	SASH1	0.47
**8522**	GAS7	0.47
**10023**	FRAT1	0.47
**7301**	TYRO3	0.47
**2767**	GNA11	0.47
**9457**	FHL5	0.47
**4094**	MAF	0.47
**65268**	WNK2	0.47
**54585**	LZTFL1	0.47
**375449**	MAST4	0.47
**138311**	FAM69B	0.47
**160622**	GRASP	0.47
**22837**	COBLL1	0.47
**51435**	SCARA3	0.47
**217**	ALDH2	0.47
**6236**	RRAD	0.47
**8322**	FZD4	0.47
**653275**	CFC1B	0.47
**10908**	PNPLA6	0.47
**57526**	PCDH19	0.47
**8424**	BBOX1	0.47
**9905**	SGSM2	0.48
**10435**	CDC42EP2	0.48
**23087**	TRIM35	0.48
**60314**	C12orf10	0.48
**1073**	CFL2	0.48
**5256**	PHKA2	0.49
**92922**	CCDC102A	0.48
**65057**	ACD	0.48
**9095**	TBX19	0.48
**6441**	SFTPD	0.48
**22846**	VASH1	0.48
**51066**	C3orf32	0.48
**23179**	RGL1	0.48
**4664**	NAB1	0.48
**50511**	SYCP3	0.48
**6430**	SRSF5	0.48
**11078**	TRIOBP	0.48
**78991**	PCYOX1L	0.48
**6623**	SNCG	0.48
**23384**	SPECC1L	0.48
**53826**	FXYD6	0.48
**9397**	NMT2	0.48
**6041**	RNASEL	0.48
**113510**	HELQ	0.48
**64788**	LMF1	0.48
**2217**	FCGRT	0.48
**79720**	VPS37B	0.48
**6764**	ST5	0.48
**252969**	NEIL2	0.48
**8987**	STBD1	0.48
**41**	ACCN2	0.48
**7905**	REEP5	0.48
**5919**	RARRES2	0.48
**10544**	PROCR	0.48
**6876**	TAGLN	0.48
**8436**	SDPR	0.49
**23500**	DAAM2	0.49
**130132**	RFTN2	0.49
**80310**	PDGFD	0.49
**4215**	MAP3K3	0.49
**282775**	OR5J2	0.49
**51161**	C3orf18	0.49
**29098**	RANGRF	0.49
**53336**	CPXCR1	0.49
**9081**	PRY	0.49
**9459**	ARHGEF6	0.49
**2995**	GYPC	0.49
**23057**	NMNAT2	0.49
**4669**	NAGLU	0.49
**6452**	SH3BP2	0.49
**6237**	RRAS	0.49
**5288**	PIK3C2G	0.49
**10252**	SPRY1	0.49
**79026**	AHNAK	0.49
**9693**	RAPGEF2	0.49
**51226**	COPZ2	0.49
**158326**	FREM1	0.49
**1956**	EGFR	0.49
**5360**	PLTP	0.49
**290**	ANPEP	0.49
**1756**	DMD	0.49
**5118**	PCOLCE	0.49
**56654**	NPDC1	0.49
**9254**	CACNA2D2	0.49
**55536**	CDCA7L	0.49
**124975**	GGT6	0.49
**1906**	EDN1	0.49
**81029**	WNT5B	0.49
**2646**	GCKR	0.49
**9811**	CTIF	0.50
**145376**	PPP1R36	0.50
**222865**	TMEM130	0.50
**92999**	ZBTB47	0.50
**168002**	DACT2	0.50
**6829**	SUPT5H	0.50
**9992**	KCNE2	0.50
**58509**	C19orf29	0.50
**79706**	PRKRIP1	0.50
**1153**	CIRBP	0.50
**9639**	ARHGEF10	0.50
**4054**	LTBP3	0.50
**1120**	CHKB	0.50
**286046**	XKR6	0.50
**9590**	AKAP12	0.50
**64115**	C10orf54	0.50
**2067**	ERCC1	0.50
**7507**	XPA	0.50
**22897**	CEP164	0.50
**652**	BMP4	0.50
**55702**	CCDC94	0.50
**57613**	KIAA1467	0.50
**28514**	DLL1	0.50
**169270**	ZNF596	0.50
**83982**	IFI27L2	0.50
**51458**	RHCG	0.49
**1112**	FOXN3	0.49
**29954**	POMT2	0.49
**9612**	NCOR2	0.49
**3198**	HOXA1	0.49
**5311**	PKD2	0.49
**2946**	GSTM2	0.49
**2109**	ETFB	0.49
**56062**	KLHL4	0.49
**6915**	TBXA2R	0.50
**64288**	ZNF323	0.50
**5195**	PEX14	0.50
**84557**	MAP1LC3A	0.50
**6164**	RPL34	0.50
**8835**	SOCS2	0.50
**2735**	GLI1	0.50
**26022**	TMEM98	0.50
**3908**	LAMA2	0.50
**1825**	DSC3	0.50
**5730**	PTGDS	0.50
**162515**	SLC16A11	0.51
**274**	BIN1	0.51
**79654**	HECTD3	0.51
**22863**	ATG14	0.51
**25949**	SYF2	0.51
**84872**	ZC3H10	0.51
**23187**	PHLDB1	0.51
**5434**	POLR2E	0.51
**6181**	RPLP2	0.51
**6141**	RPL18	0.51
**84747**	UNC119B	0.51
**23399**	CTDNEP1	0.51
**599**	BCL2L2	0.51
**197258**	FUK	0.51
**5207**	PFKFB1	0.51
**8131**	NPRL3	0.51
**25839**	COG4	0.51
**10816**	SPINT3	0.51
**60485**	SAV1	0.51
**5681**	PSKH1	0.51
**80318**	GKAP1	0.51
**57088**	PLSCR4	0.51
**93129**	ORAI3	0.51
**5829**	PXN	0.51
**2247**	FGF2	0.50
**26248**	OR2K2	0.50
**84303**	CHCHD6	0.50
**3615**	IMPDH2	0.50
**1813**	DRD2	0.50
**80148**	PQLC1	0.50
**390081**	OR52E4	0.50
**352954**	GATS	0.50
**90871**	C9orf123	0.50
**50945**	TBX22	0.52
**5204**	PFDN5	0.52
**5338**	PLD2	0.52
**94**	ACVRL1	0.52
**54039**	PCBP3	0.52
**7691**	ZNF132	0.52
**338**	APOB	0.52
**84658**	EMR3	0.52
**283232**	TMEM80	0.52
**5430**	POLR2A	0.52
**54623**	PAF1	0.52
**11070**	TMEM115	0.52
**10395**	DLC1	0.52
**57140**	RNPEPL1	0.52
**79781**	IQCA1	0.52
**1838**	DTNB	0.52
**51386**	EIF3L	0.52
**56919**	DHX33	0.52
**57542**	KLHDC5	0.52
**3628**	INPP1	0.52
**4520**	MTF1	0.52
**8547**	FCN3	0.52
**60401**	EDA2R	0.52
**8082**	SSPN	0.52
**80755**	AARSD1	0.52
**710**	SERPING1	0.52
**56246**	MRAP	0.52
**10555**	AGPAT2	0.52
**949**	SCARB1	0.52
**23743**	BHMT2	0.52
**3910**	LAMA4	0.52
**60370**	AVPI1	0.52
**5021**	OXTR	0.52
**55997**	CFC1	0.52
**23144**	ZC3H3	0.52
**56776**	FMN2	0.51
**85456**	TNKS1BP1	0.51
**283**	ANG	0.51
**7035**	TFPI	0.51
**51232**	CRIM1	0.51
**112616**	CMTM7	0.51
**22981**	NINL	0.51
**8727**	CTNNAL1	0.51
**9902**	MRC2	0.51
**10900**	RUNDC3A	0.51
**51299**	NRN1	0.51
**79632**	FAM184A	0.52
**80820**	EEPD1	0.52
**150709**	ANKAR	0.52
**6591**	SNAI2	0.52
**10129**	FRY	0.52
**5166**	PDK4	0.52
**146433**	IL34	0.52
**118812**	MORN4	0.53
**10516**	FBLN5	0.53
**9463**	PICK1	0.53
**127495**	LRRC39	0.53
**7753**	ZNF202	0.53
**79827**	CLMP	0.53
**203260**	CCDC107	0.53
**83657**	DYNLRB2	0.53

**Table S1:** Up-regulated genes found in dataset GSE29174. There are 437 up-regulated genes in this list. *Q*-values reported by SAM were 0 for all genes in this list.

NCBI gene ID	Gene Symbol	Fold Change
**1300**	COL10A1	42.74
**3007**	HIST1H1D	29.72
**8366**	HIST1H4B	25.58
**6286**	S100P	25.19
**1301**	COL11A1	24.72
**3627**	CXCL10	17.83
**4283**	CXCL9	15.88
**1387**	CREBBP	12.83
**27299**	ADAMDEC1	12.78
**54986**	ULK4	12.46
**55771**	PRR11	12.02
**54790**	TET2	11.25
**6241**	RRM2	10.60
**3433**	IFIT2	10.49
**6999**	TDO2	9.73
**1656**	DDX6	9.72
**55088**	C10orf118	9.37
**9648**	GCC2	9.24
**6696**	SPP1	8.92
**2803**	GOLGA4	8.57
**83540**	NUF2	7.73
**10112**	KIF20A	7.66
**9833**	MELK	7.59
**55165**	CEP55	7.50
**10142**	AKAP9	7.44
**9447**	AIM2	7.42
**54443**	ANLN	5.79
**6710**	SPTB	5.71
**7272**	TTK	5.64
**10635**	RAD51AP1	5.49
**4069**	LYZ	5.37
**55183**	RIF1	5.34
**891**	CCNB1	5.34
**91543**	RSAD2	5.31
**81610**	FAM83D	5.24
**64581**	CLEC7A	5.10
**10051**	SMC4	5.02
**4085**	MAD2L1	4.96
**55872**	PBK	4.83
**991**	CDC20	4.82
**9221**	NOLC1	4.74
**2124**	EVI2B	4.66
**375248**	ANKRD36	4.66
**1164**	CKS2	4.64
**1230**	CCR1	4.62
**890**	CCNA2	4.56
**127933**	UHMK1	4.49
**10274**	STAG1	4.45
**597**	BCL2A1	4.43
**55355**	HJURP	4.41
**54210**	TREM1	4.36
**253558**	LCLAT1	4.26
**2706**	GJB2	7.33
**6498**	SKIL	7.13
**219285**	SAMD9L	7.06
**10261**	IGSF6	7.01
**2335**	FN1	6.95
**699**	BUB1	6.75
**1058**	CENPA	6.75
**332**	BIRC5	6.73
**51203**	NUSAP1	6.59
**259266**	ASPM	6.54
**1063**	CENPF	6.49
**165918**	RNF168	6.44
**9232**	PTTG1	6.34
**5996**	RGS1	6.07
**29089**	UBE2T	5.96
**22974**	TPX2	5.94
**4321**	MMP12	5.91
**983**	CDK1	5.89
**85444**	LRRCC1	5.87
**29121**	CLEC2D	3.83
**4090**	SMAD5	3.80
**2123**	EVI2A	3.80
**57695**	USP37	3.79
**133418**	EMB	3.76
**4131**	MAP1B	3.76
**9787**	DLGAP5	3.75
**9768**	KIAA0101	3.74
**54625**	PARP14	3.73
**2215**	FCGR3B	3.71
**9134**	CCNE2	3.70
**3117**	HLA-DQA1	3.68
**10380**	BPNT1	3.67
**79056**	PRRG4	3.63
**10673**	TNFSF13B	3.63
**8467**	SMARCA5	3.61
**115908**	CTHRC1	3.61
**3428**	IFI16	3.61
**1520**	CTSS	3.61
**10797**	MTHFD2	3.57
**55681**	SCYL2	3.57
**9749**	PHACTR2	3.57
**94240**	EPSTI1	3.56
**64151**	NCAPG	3.51
**25879**	DCAF13	3.51
**1033**	CDKN3	4.24
**79801**	SHCBP1	4.23
**126731**	C1orf96	4.21
**6772**	STAT1	4.20
**55729**	ATF7IP	4.14
**6713**	SQLE	4.14
**157570**	ESCO2	4.10
**79871**	RPAP2	4.09
**9493**	KIF23	4.09
**4751**	NEK2	4.05
**10631**	POSTN	4.03
**23515**	MORC3	4.02
**7153**	TOP2A	4.02
**10403**	NDC80	4.00
**10915**	TCERG1	3.99
**57650**	KIAA1524	3.99
**23049**	SMG1	3.93
**80231**	CXorf21	3.87
**5111**	PCNA	3.86
**79682**	MLF1IP	3.11
**29123**	ANKRD11	3.09
**5429**	POLH	3.09
**701**	BUB1B	3.07
**200030**	NBPF11	3.06
**55677**	IWS1	3.06
**160418**	TMTC3	3.04
**9147**	NEMF	3.04
**11320**	MGAT4A	3.04
**5238**	PGM3	3.03
**2820**	GPD2	3.02
**388886**	FAM211B	3.01
**7852**	CXCR4	3.00
**57082**	CASC5	2.99
**22926**	ATF6	2.98
**7594**	ZNF43	2.98
**968**	CD68	2.97
**7171**	TPM4	2.96
**11004**	KIF2C	2.96
**10808**	HSPH1	2.95
**84909**	C9orf3	2.94
**1894**	ECT2	2.93
**1629**	DBT	2.92
**116969**	ART5	2.90
**3227**	HOXC11	2.88
**116064**	LRRC58	3.47
**29899**	GPSM2	3.47
**135114**	HINT3	3.45
**27333**	GOLIM4	3.43
**55839**	CENPN	3.43
**23213**	SULF1	3.41
**81671**	VMP1	3.39
**9889**	ZBED4	3.36
**3092**	HIP1	3.34
**51512**	GTSE1	3.34
**92797**	HELB	3.34
**51426**	POLK	3.30
**5611**	DNAJC3	3.30
**6596**	HLTF	3.28
**9910**	RABGAP1L	3.25
**528**	ATP6V1C1	3.23
**3833**	KIFC1	3.23
**197131**	UBR1	3.20
**29923**	HILPDA	3.20
**28998**	MRPL13	3.19
**58527**	C6orf115	3.19
**79000**	C1orf135	3.19
**9857**	CEP350	3.18
**84296**	GINS4	3.18
**81034**	SLC25A32	3.15
**55723**	ASF1B	3.14
**7110**	TMF1	3.14
**84081**	NSRP1	3.14
**23075**	SWAP70	3.12
**6726**	SRP9	2.69
**55215**	FANCI	2.68
**57590**	WDFY1	2.67
**55142**	HAUS2	2.66
**23047**	PDS5B	2.66
**5373**	PMM2	2.66
**11065**	UBE2C	2.66
**23085**	ERC1	2.66
**389197**	C4orf50	2.65
**11260**	XPOT	2.65
**29980**	DONSON	2.65
**64399**	HHIP	2.64
**6453**	ITSN1	2.63
**29108**	PYCARD	2.63
**9877**	ZC3H11A	2.62
**3149**	HMGB3	2.87
**10437**	IFI30	2.87
**57489**	ODF2L	2.87
**2151**	F2RL2	2.86
**23215**	PRRC2C	2.85
**128710**	C20orf94	2.85
**23594**	ORC6	2.84
**5205**	ATP8B1	2.83
**51430**	C1orf9	2.80
**57405**	SPC25	2.80
**112401**	BIRC8	2.80
**3606**	IL18	2.80
**115362**	GBP5	2.80
**50515**	CHST11	2.79
**83461**	CDCA3	2.79
**10744**	PTTG2	2.78
**51765**	MST4	2.77
**10926**	DBF4	2.76
**27125**	AFF4	2.75
**10615**	SPAG5	2.75
**55143**	CDCA8	2.74
**51602**	NOP58	2.74
**51478**	HSD17B7	2.73
**2209**	FCGR1A	2.73
**9958**	USP15	2.72
**5469**	MED1	2.72
**8813**	DPM1	2.70
**6731**	SRP72	2.70
**9991**	PTBP3	2.70
**79866**	BORA	2.41
**7072**	TIA1	2.40
**55632**	G2E3	2.40
**2213**	FCGR2B	2.40
**3987**	LIMS1	2.39
**829**	CAPZA1	2.39
**26973**	CHORDC1	2.38
**435**	ASL	2.38
**29979**	UBQLN1	2.38
**8548**	BLZF1	2.37
**9694**	TTC35	2.37
**55055**	ZWILCH	2.36
**4481**	MSR1	2.36
**10213**	PSMD14	2.35
**9966**	TNFSF15	2.35
**81624**	DIAPH3	2.62
**79723**	SUV39H2	2.61
**55789**	DEPDC1B	2.61
**10097**	ACTR2	2.59
**23036**	ZNF292	2.58
**22936**	ELL2	2.57
**8477**	GPR65	2.57
**23397**	NCAPH	2.57
**3015**	H2AFZ	2.54
**55749**	CCAR1	2.53
**25937**	WWTR1	2.52
**360023**	ZBTB41	2.51
**5080**	PAX6	2.51
**4193**	MDM2	2.51
**24137**	KIF4A	2.51
**9212**	AURKB	2.51
**168850**	ZNF800	2.50
**55109**	AGGF1	2.49
**23185**	LARP4B	2.49
**51571**	FAM49B	2.49
**51077**	FCF1	2.49
**23167**	EFR3A	2.49
**23468**	CBX5	2.48
**5396**	PRRX1	2.48
**10096**	ACTR3	2.47
**10308**	ZNF267	2.47
**6782**	HSPA13	2.47
**3832**	KIF11	2.47
**917**	CD3G	2.47
**80821**	DDHD1	2.46
**52**	ACP1	2.46
**4179**	CD46	2.46
**10499**	NCOA2	2.44
**60558**	GUF1	2.44
**55676**	SLC30A6	2.43
**6646**	SOAT1	2.43
**5440**	POLR2K	2.43
**84955**	NUDCD1	2.42
**54739**	XAF1	2.42
**84295**	PHF6	2.23
**7295**	TXN	2.23
**2710**	GK	2.23
**10905**	MAN1A2	2.22
**6780**	STAU1	2.22
**51582**	AZIN1	2.35
**54843**	SYTL2	2.34
**9039**	UBA3	2.33
**933**	CD22	2.33
**5685**	PSMA4	2.33
**9885**	OSBPL2	2.33
**9262**	STK17B	2.33
**56942**	C16orf61	2.32
**10767**	HBS1L	2.32
**87178**	PNPT1	2.32
**6303**	SAT1	2.32
**7316**	UBC	2.32
**4205**	MEF2A	2.32
**85465**	EPT1	2.31
**84640**	USP38	2.31
**5810**	RAD1	2.30
**64397**	ZFP106	2.29
**5706**	PSMC6	2.29
**22948**	CCT5	2.29
**10672**	GNA13	2.29
**339344**	MYPOP	2.28
**7292**	TNFSF4	2.28
**57103**	C12orf5	2.28
**388403**	YPEL2	2.28
**54876**	DCAF16	2.27
**113235**	SLC46A1	2.27
**11177**	BAZ1A	2.27
**339175**	METTL2A	2.26
**26586**	CKAP2	2.26
**55785**	FGD6	2.26
**24145**	PANX1	2.25
**253461**	ZBTB38	2.25
**23232**	TBC1D12	2.25
**995**	CDC25C	2.25
**55974**	SLC50A1	2.25
**472**	ATM	2.25
**23008**	KLHDC10	2.24
**10024**	TROAP	2.24
**9521**	EEF1E1	2.24
**7402**	UTRN	2.09
**55589**	BMP2K	2.08
**158747**	MOSPD2	2.08
**56886**	UGGT1	2.07
**203100**	HTRA4	2.07
**10282**	BET1	2.22
**134430**	WDR36	2.21
**4299**	AFF1	2.21
**6747**	SSR3	2.21
**7334**	UBE2N	2.21
**5965**	RECQL	2.21
**4605**	MYBL2	2.2
**6093**	ROCK1	2.19
**161725**	OTUD7A	2.19
**23518**	R3HDM1	2.18
**2239**	GPC4	2.18
**28977**	MRPL42	2.18
**64859**	OBFC2A	2.18
**3845**	KRAS	2.18
**51388**	NIP7	2.18
**7586**	ZKSCAN1	2.18
**10762**	NUP50	2.17
**7328**	UBE2H	2.17
**10730**	YME1L1	2.17
**23093**	TTLL5	2.17
**6790**	AURKA	2.17
**22889**	KIAA0907	2.17
**10875**	FGL2	2.17
**23161**	SNX13	2.17
**9169**	SCAF11	2.16
**1788**	DNMT3A	2.15
**9088**	PKMYT1	2.15
**23033**	DOPEY1	2.13
**89882**	TPD52L3	2.13
**6556**	SLC11A1	2.13
**64216**	TFB2M	2.13
**3071**	NCKAP1L	2.13
**51068**	NMD3	2.13
**509**	ATP5C1	2.13
**953**	ENTPD1	2.13
**51105**	PHF20L1	2.13
**5062**	PAK2	2.13
**9205**	ZMYM5	2.12
**55157**	DARS2	2.12
**8520**	HAT1	2.11
**79739**	TTLL7	2.11
**9495**	AKAP5	2.10
**3181**	HNRNPA2B 1	2.10
**55279**	ZNF654	2.07
**54499**	TMCO1	2.07
**81930**	KIF18A	2.07
**142686**	ASB14	2.06
**55209**	SETD5	2.06
**9736**	USP34	2.04
**116285**	ACSM1	2.04
**2201**	FBN2	2.04
**963**	CD53	2.04
**55159**	RFWD3	2.03
**9871**	SEC24D	2.03
**9887**	SMG7	2.02
**23376**	UFL1	2.02
**79646**	PANK3	2.01
**50613**	UBQLN3	2.00
**201595**	STT3B	2.00
**59345**	GNB4	1.99
**5876**	RABGGTB	1.99
**79820**	CATSPERB	1.99
**6637**	SNRPG	1.99
**51330**	TNFRSF12A	1.99
**9928**	KIF14	1.99
**286097**	EFHA2	1.98
**9131**	AIFM1	1.98
**488**	ATP2A2	1.98
**23042**	PDXDC1	1.98
**7114**	TMSB4X	1.98
**9123**	SLC16A3	1.98
**54454**	ATAD2B	1.97
**23143**	LRCH1	1.97
**4212**	MEIS2	1.97
**1457**	CSNK2A1	1.97
**80012**	PHC3	1.97
**128497**	SPATA25	1.96
**186**	AGTR2	1.96
**53981**	CPSF2	1.96
**56996**	SLC12A9	1.96
**1584**	CYP11B1	1.96
**133619**	PRRC1	1.96
**4288**	MKI67	1.96
**9014**	TAF1B	1.96
**55858**	TMEM165	1.96
**2212**	FCGR2A	1.96
**389898**	UBE2NL	2.10
**29850**	TRPM5	2.10
**3070**	HELLS	2.10
**331**	XIAP	2.09
**55751**	TMEM184C	2.09
**2146**	EZH2	2.09
**26057**	ANKRD17	1.95
**128061**	C1orf131	1.95
**64090**	GAL3ST2	1.94
**130507**	UBR3	1.93
**2298**	FOXD4	1.93
**123169**	LEO1	1.93
**57187**	THOC2	1.93
**148789**	B3GALNT2	1.93
**58508**	MLL3	1.92
**5701**	PSMC2	1.92
**148066**	ZNRF4	1.92
**6670**	SP3	1.92
**10075**	HUWE1	1.96
**220988**	HNRNPA3	1.96
**80146**	UXS1	1.95
**122011**	CSNK1A1L	1.95
**150468**	CKAP2L	1.95
**84624**	FNDC1	1.95
**7332**	UBE2L3	1.92
**3336**	HSPE1	1.92
**54800**	KLHL24	1.92
**2290**	FOXG1	1.91
**50848**	F11R	1.91
**10627**	MYL12A	1.91
**5074**	PAWR	1.91
**6476**	SI	1.91
**1009**	CDH11	1.90
**29066**	ZC3H7A	1.90
**51319**	RSRC1	1.90

**Table S4:** Result of ROC curve analysis on our miRNA array data. ROC analysis was done to validate the diagnostic value of the miRNA in the miRNA-regulated PINs.

miRBase Accession	miRNA name	AUC	*p*-value
**MIMAT0002856**	hsa-miR-520d-3p	0.49	0.549112
**MIMAT0000265**	hsa-miR-204-5p	0.98	6.47 × 10 ^−10^ ***
**MIMAT0000272**	hsa-miR-215	0.21	0.999782
**MIMAT0000271**	hsa-miR-214-3p	0.68	0.010387 *
**MIMAT0002820**	hsa-miR-497-5p	0.99	2.75 × 10 ^−10^ ***
**MIMAT0000076**	hsa-miR-21-5p	0.78	0.000184 ***
**MIMAT0000738**	hsa-miR-383	0.60	0.106284
**MIMAT0000423**	hsa-miR-125b-5p	0.99	2.48 × 10 ^−10^ ***
**MIMAT0000064**	hsa-let-7c	0.93	3.79 × 10 ^−8^ ***
**MIMAT0000089**	hsa-miR-31-5p	0.80	8.63 × 10 ^−5^ ***
**MIMAT0000077**	hsa-miR-22-3p	0.27	0.99749
**MIMAT0000098**	hsa-miR-100-5p	0.98	5.55 × 10 ^−10^ ***
**MIMAT0000097**	hsa-miR-99a-5p	0.99	2.55 × 10 ^−10^ ***
**MIMAT0000443**	hsa-miR-125a-5p	0.31	0.990694
**MIMAT0002819**	hsa-miR-193b-3p	0.41	0.86128
**MIMAT0000250**	hsa-miR-139-5p	0.99	2.42 × 10 ^−10^ ***
**MIMAT0000437**	hsa-miR-145-5p	0.96	3.14 × 10 ^−9^ ***
**MIMAT0000421**	hsa-miR-122-5p	0.48	0.597483

AUC: area under (ROC) curve;

* *p*-value < 0.05;

*** *p*-value < 0.001.

**Table S5:** Summary of constructed miRNA-regulated networks. L0 gene: genes connected directly to the miRNA (*i.e.*, direct target of miRNA); L1 gene: genes not connected directly to the miRNA.

miRBase Accession	miR name	Total gene count	L0 count	L1 count
**MIMAT0002819**	hsa-miR-193b-3p	16	1	15
**MIMAT0000250**	hsa-miR-139-5p	28	10	18
**MIMAT0000437**	hsa-miR-145-5p	86	22	64
**MIMAT0000423**	hsa-miR-125b-5p	211	16	195
**MIMAT0000443**	hsa-miR-125a-5p	206	14	192
**MIMAT0000097**	hsa-miR-99a-5p	14	1	13
**MIMAT0000265**	hsa-miR-204-5p	64	18	46
**MIMAT0000076**	hsa-miR-21-5p	91	16	75
**MIMAT0000064**	hsa-let-7c	96	20	76
**MIMAT0000421**	hsa-miR-122-5p	5	3	2
**MIMAT0000098**	hsa-miR-100-5p	14	1	13
**MIMAT0000272**	hsa-miR-215	3	3	0
**MIMAT0000271**	hsa-miR-214-3p	14	8	6
**MIMAT0000738**	hsa-miR-383	34	3	31
**MIMAT0002856**	hsa-miR-520d-3p	146	23	123
**MIMAT0000077**	hsa-miR-22-3p	46	11	35
**MIMAT0002820**	hsa-miR-497-5p	267	32	235
**MIMAT0000089**	hsa-miR-31-5p	34	3	31

**Table S6:** Specific enriched GO terms of each miRNA-regulated PINs. Genes annotated with the specific GO term in the PIN were also listed in this table. Adj. *p*-value: multiple-test adjusted p-value calculated by the method described in the work of Benjamini and Yekutieli [48].

MIMAT0002856(hsa-miR-520d-3p)		

GO term	Genes	Adj. *p*-value
GO:0007169, Transmembrane receptor protein tyrosine kinase signaling pathway	SH3KBP1, HDAC2, RET, ABI1, LYN, GRB2, SORBS1, CLTC, CLTA, CDC42, CASP9, RAF1, SRC, AP2A1, AP2B1, MAPK3, ARHGEF7, PRKCA, RPS6, PRKAR2B, MAPK1, ARHGEF6, CDK1, SH3GL2, EIF4G1, HDAC1, ECT2, MKNK1, CASP3, PRKACA, ADRB2, PRKAR2A, EIF4B, SHC1, RAC1	2.77 × 10 ^−29^

GO:0048011, Nerve growth factor receptor signaling pathway	HDAC2, GRB2, CLTC, CLTA, CASP9, RAF1, SRC, AP2A1, AP2B1, MAPK3, ARHGEF7, PRKCA, PRKAR2B, MAPK1, ARHGEF6, CDK1, SH3GL2, HDAC1, ECT2, CASP3, PRKACA, PRKAR2A, SHC1, RAC1	5.09 × 10 ^−24^

GO:0007173, Epidermal growth factor receptor signaling pathway	SH3KBP1, GRB2, CLTC, CLTA, CDC42, CASP9, RAF1, SRC, AP2A1, AP2B1, MAPK3, ARHGEF7, PRKCA, PRKAR2B, MAPK1, CDK1, SH3GL2, PRKACA, PRKAR2A, SHC1	2.96 × 10 ^−22^

GO:0043067, Regulation of programmed cell death	HDAC2, STK17B, ESR1, ABL1, LYN, TP53, GABRB3, PAK2, LCK, CASP9, RAF1, PLK1, ARHGEF7, PRKCA, RPS6, SH3RF1, MAPK1, IFT57, ARHGAP10, ARHGEF6, CDK1, APAF1, HDAC1, ECT2, CASP3, SOX10, EP300, ARAF, TFAP2A, ADRB2, HCK, KLHL20, CASP8, HIP1, RAC1	4.76 × 10 ^−19^

GO:0042058, Regulation of epidermal growth factor receptor signaling pathway	SH3KBP1, ESR1, GRB2, CLTC, CLTA, CDC42, AP2A1, AP2B1, ARHGEF7, SH3GL2, SHC1	2.36 × 10 ^−12^

GO:0008543, Fibroblast growth factor receptor signaling pathway	GRB2, CASP9, RAF1, SRC, MAPK3, PRKCA, PRKAR2B, MAPK1, CDK1, MKNK1, PRKACA, PRKAR2A, SHC1	2.39 × 10 ^−12^

GO:0043068, Positive regulation of programmed cell death	STK17B, ABL1, LYN, TP53, LCK, CASP9, ARHGEF7, PRKCA, RPS6, SH3RF1, MAPK1, ARHGEF6, APAF1, ECT2, CASP3, EP300, TFAP2A, ADRB2, CASP8, HIP1, RAC1	3.09 × 10 ^−12^

GO:0010942, Positive regulation of cell death	STK17B, ABL1, LYN, TP53, LCK, CASP9, ARHGEF7, PRKCA, RPS6, SH3RF1, MAPK1, ARHGEF6, APAF1, ECT2, CASP3, EP300, TFAP2A, ADRB2, CASP8, HIP1, RAC1	4.49 × 10 ^−12^

GO:0006917, Induction of apoptosis	STK17B, ABL1, TP53, LCK, CASP9, ARHGEF7, PRKCA, SH3RF1, MAPK1, ARHGEF6, APAF1, ECT2, CASP3, EP300, CASP8, HIP1, RAC1	6.91 × 10 ^−11^

GO:0012502, Induction of programmed cell death	STK17B, ABL1, TP53, LCK, CASP9, ARHGEF7, PRKCA, SH3RF1, MAPK1, ARHGEF6, APAF1, ECT2, CASP3, EP300, CASP8, HIP1, RAC1	7.42 × 10 ^−11^

GO:0042059, Negative regulation of epidermal growth factor receptor signaling pathway	SH3KBP1, GRB2, CLTC, CLTA, CDC42, AP2A1, AP2B1, ARHGEF7, SH3GL2	8.63 × 10 ^−11^

GO:0015630, Microtubule cytoskeleton	STMN1, SORBS1, SMAD4, CLTC, CDC42, LCK, RACGAP1, PLK1, PRKAR2B, YES1, MAPK1, IFT57, CDK1, ECT2, PRKACA, RB1, EP300, CCNB1, CHAF1B, TFAP2A, CASP8, PRKAR2A	4.75 × 10 ^−10^

GO:0060548, Negative regulation of cell death	HDAC2, ESR1, TP53, SMAD4, RAF1, PLK1, PRKCA, RPS6, SH3RF1, CDK1, HDAC1, CASP3, SOX10, ARAF, TFAP2A, HCK, KLHL20	6.27 × 10 ^−8^

GO:0008286, Insulin receptor signaling pathway	GRB2, SORBS1, RAF1, MAPK3, RPS6, MAPK1, CDK1, EIF4G1, EIF4B, SHC1	2.15 × 10 ^−7^

GO:0043069, Negative regulation of programmed cell death	HDAC2, ESR1, TP53, RAF1, PLK1, PRKCA, RPS6, SH3RF1, CDK1, HDAC1, CASP3, SOX10, ARAF, TFAP2A, HCK, KLHL20	3.13 × 10 ^−7^

GO:0008284, Positive regulation of cell proliferation	HDAC2, ESR1, LYN, CDC42, E2F1, PRKCA, MAPK1, CDK1, RHOG, HDAC1, NCK1, SOX10, CCNB1, ADRB2, HCK, SHC1	8.34 × 10 ^−7^

GO:0051988, Regulation of attachment of spindle microtubules to kinetochore	CDC42, RACGAP1, ECT2, CCNB1	3.27 × 10 ^−5^

GO:0008629, Induction of apoptosis by intracellular signals	ABL1, TP53, CASP9, APAF1, CASP3, EP300, CASP8	5.83 × 10 ^−5^

**MIMAT0002820(hsa-miR-497-5p)**		

**GO term**	**Genes**	**Adj. *p* -value**

GO:0043067, Regulation of programmed cell death	ESR1, MEN1, ABL1, HIPK3, PPARGC1A, SIAH1, SH3RF1, PAK2, LCK, MED1, PPARG, CBX4, ARHGEF7, YWHAB, RXRA, ACVR1, MAPK1, CASP3, CASP6, AR, PTPRF, MDM2, BRCA1, MLH1, RAB27A, PIAS4, FAF1, RAC1, VHL, SKI, NR4A1, LYN, TP53, PSMC2, GATA1, GATA6, GATA3, RAF1, CDKN1B, PLK1, PSMD11, HOXA13, RPS6, ESR2, ARHGAP10, ARHGEF6, SMAD3, SKIL, RYR2, PSEN1, HCK, TRAF2	2.67 × 10 ^−25^

GO:0043068, Positive regulation of programmed cell death	MEN1, ABL1, SIAH1, SH3RF1, LCK, PPARG, ARHGEF7, YWHAB, RXRA, MAPK1, CASP3, CASP6, PTPRF, BRCA1, MLH1, RAB27A, PIAS4, FAF1, RAC1, NR4A1, LYN, TP53, GATA6, CDKN1B, HOXA13, RPS6, ESR2, ARHGEF6, SMAD3, RYR2, PSEN1, TRAF2	1.74 × 10 ^−17^

GO:0010942, Positive regulation of cell death	MEN1, ABL1, SIAH1, SH3RF1, LCK, PPARG, ARHGEF7, YWHAB, RXRA, MAPK1, CASP3, CASP6, PTPRF, BRCA1, MLH1, RAB27A, PIAS4, FAF1, RAC1, NR4A1, LYN, TP53, GATA6, CDKN1B, HOXA13, RPS6, ESR2, ARHGEF6, SMAD3, RYR2, PSEN1, TRAF2	3.08 × 10 ^−17^
GO:0008285, Negative regulation of cell proliferation	MEN1, MED1, PPARG, RXRA, CASP3, AR, PTPRF, VDR, VHL, SKI, LYN, TP53, TOB1, GATA1, GATA3, RAF1, HNF4A, CDKN1B, BRD7, MED25, ESR2, ABI1, SMAD1, SMAD2, SMAD3, SMAD4, SOX7	3.85 × 10 ^−14^

GO:0015629, Actin cytoskeleton	ABL1, SORBS1, FLNA, SEPT7, ANLN, MACF1, HAP1, SH3PXD2A, IQGAP2, BRCA1, ACTC1, ACTA1, MYL2, MYLK, SORBS2, ARPC4, ARPC5, ACTR2, ACTR3, ARPC1B, WASF1, WASF2, HCK	2.52 × 10 ^−13^

GO:0006917, Induction of apoptosis	ABL1, SH3RF1, LCK, PPARG, ARHGEF7, YWHAB, MAPK1, CASP3, CASP6, BRCA1, MLH1, RAB27A, RAC1, NR4A1, TP53, CDKN1B, ARHGEF6, SMAD3, RYR2, PSEN1, TRAF2	9.69 × 10 ^−11^

GO:0012502, Induction of programmed cell death	ABL1, SH3RF1, LCK, PPARG, ARHGEF7, YWHAB, MAPK1, CASP3, CASP6, BRCA1, MLH1, RAB27A, RAC1, NR4A1, TP53, CDKN1B, ARHGEF6, SMAD3, RYR2, PSEN1, TRAF2	1.06 × 10 ^−10^

GO:0007178, Transmembrane receptor protein serine/threonine kinase signaling pathway	ACVR1, SMURF2, SKI, GDF6, BMP6, ZNF8, GATA4, HNF4A, SMAD1, SMAD2, SMAD3, SMAD4, SMAD5, RYR2	1.22 × 10 ^−10^

GO:0007169, Transmembrane receptor protein tyrosine kinase signaling pathway	SORBS1, CDC42, SRC, MAPK3, ARHGEF7, YWHAB, MAPK1, SH3GL2, CASP3, MDM2, EIF4G1, RAC1, SH3KBP1, NR4A1, LYN, GRB2, RAF1, CDKN1B, RPS6, ABI1, ARHGEF6, MKNK1, PSEN1, EIF4B	1.67 × 10 ^−10^

GO:0090092, Regulation of transmembrane receptor protein serine/threonine kinase signaling pathway	MEN1, ACVR1, SMURF2, SKI, GDF6, TP53, BMP6, GATA4, GATA6, HOXA13, SMAD2, SMAD3, SMAD4, SKIL	7.51 × 10 ^−10^

GO:0030509, BMP signaling pathway	ACVR1, SMURF2, SKI, GDF6, BMP6, ZNF8, SMAD1, SMAD4, SMAD5, RYR2	3.25 × 10 ^−9^

GO:0060548, Negative regulation of cell death	ESR1, HIPK3, PPARGC1A, SH3RF1, MED1, CBX4, ACVR1, CASP3, AR, MDM2, VHL, TP53, GATA1, GATA6, GATA3, RAF1, CDKN1B, PLK1, RPS6, SMAD3, SMAD4, PSEN1, HCK	7.88 × 10 ^−9^

GO:0007173, Epidermal growth factor receptor signaling pathway	CDC42, SRC, MAPK3, ARHGEF7, YWHAB, MAPK1, SH3GL2, MDM2, SH3KBP1, NR4A1, GRB2, RAF1, CDKN1B	9.22 × 10 ^−9^

GO:0030521, Androgen receptor signaling pathway	PPARGC1A, MED14, MED1, AR, BRCA1, MED12, PIAS1, RAN, NR1I3	1.42 × 10 ^−8^

GO:0043069, Negative regulation of programmed cell death	ESR1, HIPK3, PPARGC1A, SH3RF1, MED1, CBX4, ACVR1, CASP3, AR, MDM2, VHL, TP53, GATA1, GATA6, GATA3, RAF1, CDKN1B, PLK1, RPS6, SMAD3, PSEN1, HCK	2.44 × 10 ^−8^

GO:0048011, Nerve growth factor receptor signaling pathway	SRC, MAPK3, ARHGEF7, YWHAB, MAPK1, SH3GL2, CASP3, MDM2, RAC1, NR4A1, GRB2, RAF1, CDKN1B, ARHGEF6, PSEN1	2.64 × 10 ^−8^

GO:0032956, Regulation of actin cytoskeleton organization	ABL1, LRP1, ARPC4, ARPC5, ACTR3, ARPC1B, SMAD3, NCK1, SORBS3, HCK, LIMK1	6.42 × 10 ^−6^

GO:0008543, Fibroblast growth factor receptor signaling pathway	SRC, MAPK3, YWHAB, MAPK1, MDM2, NR4A1, GRB2, RAF1, CDKN1B, MKNK1	9.06 × 10 ^−6^

GO:0042059, Negative regulation of epidermal growth factor receptor signaling pathway	CDC42, ARHGEF7, SH3GL2, PTPRF, SH3KBP1, GRB2, PSEN1	1.11 × 10 ^−5^

GO:0042058, Regulation of epidermal growth factor receptor signaling pathway	ESR1, CDC42, ARHGEF7, SH3GL2, PTPRF, SH3KBP1, GRB2, PSEN1	1.17 × 10 ^−5^

GO:0007179, Transforming growth factor beta receptor signaling pathway	ACVR1, SMURF2, SKI, SMAD1, SMAD2, SMAD3, SMAD4, SMAD5	1.67 × 10 ^−5^

GO:0015630, Microtubule cytoskeleton	STMN1, RIF1, SORBS1, CDC42, LCK, RACGAP1, YES1, YWHAB, MAPK1, SEPT7, KIF23, CDC16, MACF1, BRCA1, FEZ1, NCOR1, PLK1, CHD3, SMAD4, CEP350, CDC27, PSEN1	2.14 × 10 ^−5^

GO:0017015, Regulation of transforming growth factor beta receptor signaling pathway	MEN1, SMURF2, SKI, TP53, SMAD2, SMAD3, SMAD4, SKIL	2.30 × 10 ^−5^

GO:0070302, Regulation of stress-activated protein kinase signaling cascade	MEN1, ZEB2, HIPK3, SH3RF1, CDC42, MAPK3, MAPK1, LYN, NCOR1, TRAF2	2.74 × 10 ^−5^

GO:0001959, Regulation of cytokine-mediated signaling pathway	HSP90AB1, MED1, PPARG, PTPRF, NR1H2, PIAS1, IL36RN, HIPK1	6.35 × 10 ^−5^

GO:0008284, Positive regulation of cell proliferation	ESR1, CDC42, MED1, RARA, MAPK1, AR, MDM2, NR4A1, LYN, FZR1, BMP6, GATA1, GATA4, GATA6, CDKN1B, NCK1, HCLS1, HCK	7.29 × 10 ^−5^

**MIMAT0000423(hsa-miR-125b-5p)**		

**GO term**	**Genes**	**Adj. *p* -value**

GO:0043067, Regulation of programmed cell death	HMGA2, PML, PRNP, FGF2, XRCC4, BRCA1, IGFBP3, HDAC3, CTNNB1, CD5, CDK1, NKX2-5, MEF2C, PRKCI, CASP2, PSMA4, PSMA3, CFDP1, CAV1, FAF1, YWHAB, HIF1A, RELA, TCF7L2, TNFSF12, PSEN2, TP53, TOP2A, TNFRSF4, BID, MYC, JUN, OGT, CDKN1A, HOXA13, RNF7, PPP2R4, HDAC2, HDAC1, SNCA, PTEN, NFKBIA, IFI16, NOL3, TRAF2, HSP90B1	4.56 × 10 ^−24^

GO:0060548, Negative regulation of cell death	HMGA2, PRNP, FGF2, XRCC4, HDAC3, CTNNB1, CDK1, NKX2-5, MEF2C, PRKCI, CFDP1, HIF1A, RELA, TCF7L2, PSEN2, TP53, TNFRSF4, MYC, JUN, CDKN1A, RNF7, HDAC2, HDAC1, SNCA, PTEN, MGMT, NFKBIA, NOL3, HSP90B1	8.04 × 10 ^−17^

GO:0008284, Positive regulation of cell proliferation	HMGA2, FGF2, XRCC4, CDC25B, CTNNB1, EGR1, AGGF1, CDK1, NKX2-5, MEF2C, PRKCI, IRS1, HIF1A, RELA, HCLS1, TNFSF12, ARNT, PTPRC, TNFSF4, TNFRSF4, MYC, JUN, FGF1, CDKN1A, HDAC2, HDAC1, NOLC1, PTEN	2.20 × 10 ^−15^
GO:0043069, Negative regulation of programmed cell death	HMGA2, PRNP, XRCC4, HDAC3, CTNNB1, CDK1, NKX2-5, MEF2C, PRKCI, CFDP1, HIF1A, RELA, TCF7L2, PSEN2, TP53, TNFRSF4, MYC, JUN, CDKN1A, RNF7, HDAC2, HDAC1, SNCA, PTEN, NFKBIA, NOL3, HSP90B1	3.62 × 10 ^−15^

GO:0043068, Positive regulation of programmed cell death	HMGA2, PML, BRCA1, IGFBP3, CTNNB1, CD5, MEF2C, PRKCI, CASP2, CAV1, FAF1, YWHAB, TNFSF12, PSEN2, TP53, TOP2A, BID, JUN, OGT, CDKN1A, HOXA13, RNF7, PPP2R4, PTEN, IFI16, TRAF2	4.51 × 10 ^−14^

GO:0010942, Positive regulation of cell death	HMGA2, PML, BRCA1, IGFBP3, CTNNB1, CD5, MEF2C, PRKCI, CASP2, CAV1, FAF1, YWHAB, TNFSF12, PSEN2, TP53, TOP2A, BID, JUN, OGT, CDKN1A, HOXA13, RNF7, PPP2R4, PTEN, IFI16, TRAF2	7.15 × 10 ^−14^

GO:0006916, Anti-apoptosis	PRNP, HDAC3, CDK1, NKX2-5, MEF2C, PRKCI, CFDP1, RELA, TCF7L2, PSEN2, RNF7, HDAC1, SNCA, NFKBIA, NOL3, HSP90B1	1.25 × 10 ^−10^

GO:0008285, Negative regulation of cell proliferation	SERPINF1, SRF, PML, PRNP, FGF2, CSNK2B, IGFBP3, CTNNB1, CAV1, HMGA1, VDR, CDH5, HSF1, COL18A1, TP53, MYC, JUN, CDKN1A, PAK1, PTEN	2.08 × 10 ^−9^

GO:0015630, Microtubule cytoskeleton	STMN1, KIF1C, RANGAP1, CDC25B, BRCA1, HDAC3, CTNNB1, PIN4, HSPH1, RANBP9, CDK1, SPTAN1, YWHAQ, DVL1, FKBP4, YWHAB, CCDC85B, MAPT, PSEN2, TOP2A, SPIB, MYC, OGT, APEX1, PAFAH1B1	2.24 × 10 ^−9^

GO:0048011, Nerve growth factor receptor signaling pathway	HDAC3, CDK1, MEF2C, PRKCI, CASP2, IRS1, YWHAB, RELA, PSEN2, HDAC2, HDAC1, PTEN, ATF1, NFKBIA	1.99 × 10 ^−8^

GO:0050678, Regulation of epithelial cell proliferation	SERPINF1, PGR, FGF2, CTNNB1, AGGF1, CAV1, HIF1A, TNFSF12, ARNT, MYC, JUN, FGF1, PTEN	3.41 × 10 ^−8^

GO:0007169, Transmembrane receptor protein tyrosine kinase signaling pathway	FGF2, HDAC3, CDK1, MEF2C, PRKCI, CASP2, IRS1, FIBP, PTPN1, YWHAB, RELA, PSEN2, FGF1, HDAC2, HDAC1, PTEN, ATF1, NFKBIA, EIF4EBP1	6.39 × 10 ^−8^
GO:0006917, Induction of apoptosis	PML, BRCA1, CD5, CASP2, CAV1, YWHAB, TNFSF12, PSEN2, TP53, BID, OGT, CDKN1A, RNF7, PTEN, IFI16, TRAF2	1.68 × 10 ^−7^

GO:0012502, Induction of programmed cell death	PML, BRCA1, CD5, CASP2, CAV1, YWHAB, TNFSF12, PSEN2, TP53, BID, OGT, CDKN1A, RNF7, PTEN, IFI16, TRAF2	1.81 × 10 ^−7^

GO:0035666, TRIF-dependent toll-like receptor signaling pathway	ATF2, CDK1, MEF2C, FOS, RELA, JUN, ATF1, NFKBIA	2.40 × 10 ^−6^

GO:0034138, Toll-like receptor 3 signaling pathway	ATF2, CDK1, MEF2C, FOS, RELA, JUN, ATF1, NFKBIA	2.71 × 10 ^−6^

GO:0051693, Actin filament capping	SPTB, SPTBN1, SPTAN1, SPTA1, ADD1, EPB49	3.43 × 10 ^−6^
GO:0002756, MyD88- independent toll-like receptor signaling pathway	ATF2, CDK1, MEF2C, FOS, RELA, JUN, ATF1, NFKBIA	3.82 × 10 ^−6^

GO:0034134, Toll-like receptor 2 signaling pathway	ATF2, CDK1, MEF2C, FOS, RELA, JUN, ATF1, NFKBIA	5.33 × 10 ^−6^

GO:0034130, Toll-like receptor 1 signaling pathway	ATF2, CDK1, MEF2C, FOS, RELA, JUN, ATF1, NFKBIA	5.33 × 10 ^−6^

GO:0030835, Negative regulation of actin filament depolymerization	SPTB, SPTBN1, SPTAN1, SPTA1, ADD1, EPB49	5.58 × 10 ^−6^

GO:0015629, Actin cytoskeleton	WAS, CDH1, BRCA1, SPTB, SPTBN1, SPTAN1, CTDP1, STX1A, SPTA1, PAK1, SNCA, ADD1, EPB41, EPB49	5.58 × 10 ^−6^

GO:0002755, MyD88-dependent toll-like receptor signaling pathway	ATF2, CDK1, MEF2C, FOS, RELA, JUN, ATF1, NFKBIA	7.66 × 10 ^−6^

GO:0034142, Toll-like receptor 4 signaling pathway	ATF2, CDK1, MEF2C, FOS, RELA, JUN, ATF1, NFKBIA	1.14 × 10 ^−5^

GO:0050679, Positive regulation of epithelial cell proliferation	FGF2, CTNNB1, AGGF1, HIF1A, TNFSF12, ARNT, MYC, JUN, FGF1	1.14 × 10 ^−5^

**MIMAT0000076(hsa-miR-21-5p)**		

**GO term**	**Genes**	**Adj. *p* -value**

GO:0030834, Regulation of actin filament depolymerization	SPTB, SPTBN1, SPTAN1, SPTA1, ADD1, EPB49	1.27 × 10 ^−5^

GO:0030837, Negative regulation of actin filament polymerization	SPTB, SPTBN1, SPTAN1, SPTA1, ADD1, EPB49	2.71 × 10 ^−5^

GO:0002224, Toll-like receptor signaling pathway	ATF2, CDK1, MEF2C, FOS, RELA, JUN, ATF1, NFKBIA	2.96 × 10 ^−5^

GO:0008629, Induction of apoptosis by intracellular signals	PML, BRCA1, YWHAB, TP53, BID, CDKN1A, RNF7, IFI16	3.52 × 10 ^−5^

GO:0043067, Regulation of programmed cell death	SPRY2, TP53, ADAMTSL4, ETS1, TDGF1, RAF1, HOXA5, HOXA13, MSX1, MSX2, NKX2-5, CBL, INHBB, COL4A3, ACVR1C, TRAF2	1.92 × 10 ^−5^
GO:0007173, Epidermal growth factor receptor signaling pathway	SPRY2, SPRY1, GRB2, PTPN11, TDGF1, RAF1, CBL	9.44 × 10 ^−5^

**MIMAT0000250(hsa-miR-139-5p)**		

**GO term**	**Genes**	**Adj. *p* -value**

GO:0035583, Negative regulation of transforming growth factor beta receptor signaling pathway by extracellular sequestering of TGFbeta	LTBP1, FBN1, FBN2	2.61 × 10 ^−5^

**MIMAT0000089(hsa-miR-31-5p)**		

**GO term**	**Genes**	**Adj. *p* -value**

GO:0007187, G-protein signaling, coupled to cyclic nucleotide second messenger	GNA12, GNA13, DRD5, MTNR1A, S1PR3, TSHR, S1PR4	8.74 × 10 ^−7^

GO:0019935, Cyclic-nucleotidemediated signaling	GNA12, GNA13, DRD5, MTNR1A, S1PR3, TSHR, S1PR4	1.60 × 10 ^−6^

GO:0007188, G-protein signaling, coupled to cAMP nucleotide second messenger	GNA12, GNA13, DRD5, S1PR3, TSHR, S1PR4	6.82 × 10 ^−6^

GO:0048011, Nerve growth factor receptor signaling pathway	ARHGEF1, PRKCD, ARHGEF12, PRKACA, PRKCE, MCF2, ARHGEF11	6.93 × 10 ^−6^

GO:0019933, CAMP-mediated signaling	GNA12, GNA13, DRD5, S1PR3, TSHR, S1PR4	1.05 × 10 ^−5^

GO:0043067, Regulation of programmed cell death	PTK2B, PRKCD, TGFBR1, ARHGEF12, CTNNB1, PRKCE, TIA1, MCF2, FASTK, ARHGEF11, F2R	1.25 × 10 ^−5^

GO:0003376, Sphingosine-1- phosphate signaling pathway	S1PR3, S1PR2, S1PR4	3.22 × 10 ^−5^

GO:0007169, Transmembrane receptor protein tyrosine kinase signaling pathway	PTK2B, ARHGEF1, PRKCD, ARHGEF12, PRKACA, PRKCE, MCF2, ARHGEF11	5.20 × 10 ^−5^

GO:0043068, Positive regulation of programmed cell death	TGFBR1, ARHGEF12, CTNNB1, PRKCE, TIA1, MCF2, FASTK, ARHGEF11	7.67 × 10 ^−5^

GO:0010942, Positive regulation of cell death	TGFBR1, ARHGEF12, CTNNB1, PRKCE, TIA1, MCF2, FASTK, ARHGEF11	8.78 × 10 ^−5^

GO:0006917, Induction of apoptosis	TGFBR1, ARHGEF12, PRKCE, TIA1, MCF2, FASTK, ARHGEF11	9.32 × 10 ^−5^
GO:0012502, Induction of programmed cell death	TGFBR1, ARHGEF12, PRKCE, TIA1, MCF2, FASTK, ARHGEF11	9.51 × 10 ^−5^

**MIMAT0000437(hsa-miR-145-5p)**		

**GO term**	**Genes**	**Adj. *p* -value**

GO:0030509, BMP signaling pathway	BMP6, ZNF8, ACVR1, SMAD1, SMAD4, RYR2, SMAD5, SMURF2, GDF6	1.37 × 10 ^−11^

GO:0007178, Transmembrane receptor protein serine/threonine kinase signaling pathway	BMP6, ZNF8, ACVR1, SMAD1, SMAD4, RYR2, SMAD5, SMURF2, GDF6	2.56 × 10 ^−8^

GO:0090092, Regulation of transmembrane receptor protein serine/threonine kinase signaling pathway	MEN1, TP53, BMP6, HOXA13, ACVR1, SMAD4, SULF1, SMURF2, GDF6	8.22 × 10 ^−8^

**MIMAT0000064(hsa-let-7c)**		

**GO term**	**Genes**	**Adj. *p* -value**

GO:0043067, Regulation of programmed cell death	RRM2B, ACVR1, TP53, RASA1, TGFBR1, PSMA3, BIRC5, ACTN2, HOXA13, IRS2, FASTK, VAV1, PSMB6, BCL2, CDK1, HDAC1, SOX10, TIA1, AKT1, AURKB	3.43 × 10 ^−8^

GO:0043069, Negative regulation of programmed cell death	RRM2B, ACVR1, TP53, RASA1, TGFBR1, BIRC5, IRS2, BCL2, CDK1, HDAC1, SOX10, AKT1, AURKB	6.58 × 10 ^−6^

GO:0060548, Negative regulation of cell death	RRM2B, ACVR1, TP53, RASA1, TGFBR1, BIRC5, IRS2, BCL2, CDK1, HDAC1, SOX10, AKT1, AURKB	8.92 × 10 ^−6^

GO:0015630, Microtubule cytoskeleton	INCENP, SNTB2, SEPT1, TACC1, BIRC5, RACGAP1, PIN4, CDCA8, CDK1, PHF1, AKT1, AURKB, NINL, CCDC85B	5.15 × 10 ^−5^

GO:0043067, Regulation of programmed cell death	HMGA2, PML, PRNP, FGF2, XRCC4, BRCA1, IGFBP3, HDAC3, CTNNB1, CD5, CDK1, NKX2-5, MEF2C, PRKCI, CASP2, PSMA4, PSMA3, CFDP1, CAV1, FAF1, YWHAB, HIF1A, RELA, TCF7L2, TNFSF12, PSEN2, TP53, TOP2A, TNFRSF4, BID, MYC, JUN, OGT, CDKN1A, RNF7, PPP2R4, HDAC2, HDAC1, SNCA, PTEN, NFKBIA, IFI16, NOL3, TRAF2, HSP90B1	1.62 × 10 ^−23^

GO:0060548, Negative regulation of cell death	HMGA2, PRNP, FGF2, XRCC4, HDAC3, CTNNB1, CDK1, NKX2-5, MEF2C, PRKCI, CFDP1, HIF1A, RELA, TCF7L2, PSEN2, TP53, TNFRSF4, MYC, JUN, CDKN1A, RNF7, HDAC2, HDAC1, SNCA, PTEN, MGMT, NFKBIA, NOL3, HSP90B1	4.53 × 10 ^−17^

GO:0008284, Positive regulation of cell proliferation	HMGA2, FGF2, XRCC4, CDC25B, CTNNB1, EGR1, AGGF1, CDK1, NKX2-5, MEF2C, PRKCI, IRS1, HIF1A, RELA, HCLS1, TNFSF12, ARNT, PTPRC, TNFSF4, TNFRSF4, MYC, JUN, FGF1, CDKN1A, HDAC2, HDAC1, NOLC1, PTEN	1.23 × 10 ^−15^

**MIMAT0000443(hsa-miR-125a-5p)**		

**GO term**	**Genes**	**Adj. *p* -value**

GO:0043069, Negative regulation of programmed cell death	HMGA2, PRNP, XRCC4, HDAC3, CTNNB1, CDK1, NKX2-5, MEF2C, PRKCI, CFDP1, HIF1A, RELA, TCF7L2, PSEN2, TP53, TNFRSF4, MYC, JUN, CDKN1A, RNF7, HDAC2, HDAC1, SNCA, PTEN, NFKBIA, NOL3, HSP90B1	2.02 × 10 ^−15^

GO:0043068, Positive regulation of programmed cell death	HMGA2, PML, BRCA1, IGFBP3, CTNNB1, CD5, MEF2C, PRKCI, CASP2, CAV1, FAF1, YWHAB, TNFSF12, PSEN2, TP53, TOP2A, BID, JUN, OGT, CDKN1A, RNF7, PPP2R4, PTEN, IFI16, TRAF2	2.80 × 10 ^−13^

GO:0010942, Positive regulation of cell death	HMGA2, PML, BRCA1, IGFBP3, CTNNB1, CD5, MEF2C, PRKCI, CASP2, CAV1, FAF1, YWHAB, TNFSF12, PSEN2, TP53, TOP2A, BID, JUN, OGT, CDKN1A, RNF7, PPP2R4, PTEN, IFI16, TRAF2	4.35 × 10 ^−13^

GO:0006916, Anti-apoptosis	PRNP, HDAC3, CDK1, NKX2-5, MEF2C, PRKCI, CFDP1, RELA, TCF7L2, PSEN2, RNF7, HDAC1, SNCA, NFKBIA, NOL3, HSP90B1	9.04 × 10 ^−11^

GO:0008285, Negative regulation of cell proliferation	SERPINF1, SRF, PML, PRNP, FGF2, CSNK2B, IGFBP3, CTNNB1, CAV1, HMGA1, VDR, CDH5, HSF1, COL18A1, TP53, MYC, JUN, CDKN1A, PAK1, PTEN	1.32 × 10 ^−9^

GO:0015630, Microtubule cytoskeleton	STMN1, KIF1C, RANGAP1, CDC25B, BRCA1, HDAC3, CTNNB1, PIN4, HSPH1, RANBP9, CDK1, SPTAN1, YWHAQ, DVL1, FKBP4, YWHAB, CCDC85B, MAPT, PSEN2, TOP2A, SPIB, MYC, OGT, APEX1, PAFAH1B1	1.32 × 10 ^−9^

GO:0048011, Nerve growth factor receptor signaling pathway	HDAC3, CDK1, MEF2C, PRKCI, CASP2, IRS1, YWHAB, RELA, PSEN2, HDAC2, HDAC1, PTEN, ATF1, NFKBIA	1.50 × 10 ^−8^

GO:0050678, Regulation of epithelial cell proliferation	SERPINF1, PGR, FGF2, CTNNB1, AGGF1, CAV1, HIF1A, TNFSF12, ARNT, MYC, JUN, FGF1, PTEN	2.66 × 10 ^−8^

GO:0007169, Transmembrane receptor protein tyrosine kinase signaling pathway	FGF2, HDAC3, CDK1, MEF2C, PRKCI, CASP2, IRS1, FIBP, PTPN1, YWHAB, RELA, PSEN2, FGF1, HDAC2, HDAC1, PTEN, ATF1, NFKBIA, EIF4EBP1	4.37 × 10 ^−8^

GO:0006917, Induction of apoptosis	PML, BRCA1, CD5, CASP2, CAV1, YWHAB, TNFSF12, PSEN2, TP53, BID, OGT, CDKN1A, RNF7, PTEN, IFI16, TRAF2	1.22 × 10 ^−7^

GO:0012502, Induction of programmed cell death	PML, BRCA1, CD5, CASP2, CAV1, YWHAB, TNFSF12, PSEN2, TP53, BID, OGT, CDKN1A, RNF7, PTEN, IFI16, TRAF2	1.31 × 10 ^−7^

GO:0035666, TRIF-dependent toll-like receptor signaling pathway	ATF2, CDK1, MEF2C, FOS, RELA, JUN, ATF1, NFKBIA	2.01 × 10 ^−6^

GO:0034138, Toll-like receptor 3 signaling pathway	ATF2, CDK1, MEF2C, FOS, RELA, JUN, ATF1, NFKBIA	2.27 × 10 ^−6^

GO:0051693, Actin filament capping	SPTB, SPTBN1, SPTAN1, SPTA1, ADD1, EPB49	2.97 × 10 ^−6^

GO:0002756, MyD88- independent toll-like receptor signaling pathway	ATF2, CDK1, MEF2C, FOS, RELA, JUN, ATF1, NFKBIA	3.20 × 10 ^−6^

GO:0015629, Actin cytoskeleton	WAS, CDH1, BRCA1, SPTB, SPTBN1, SPTAN1, CTDP1, STX1A, SPTA1, PAK1, SNCA, ADD1, EPB41, EPB49	4.25 × 10 ^−6^

GO:0034134, Toll-like receptor 2 signaling pathway	ATF2, CDK1, MEF2C, FOS, RELA, JUN, ATF1, NFKBIA	4.43 × 10 ^−6^

GO:0034130, Toll-like receptor 1 signaling pathway	ATF2, CDK1, MEF2C, FOS, RELA, JUN, ATF1, NFKBIA	4.43 × 10 ^−6^

GO:0030835, Negative regulation of actin filament depolymerization	SPTB, SPTBN1, SPTAN1, SPTA1, ADD1, EPB49	4.72 × 10 ^−6^

GO:0002755, MyD88-dependent toll-like receptor signaling pathway	ATF2, CDK1, MEF2C, FOS, RELA, JUN, ATF1, NFKBIA	6.41 × 10 ^−6^

GO:0050679, Positive regulation of epithelial cell proliferation	FGF2, CTNNB1, AGGF1, HIF1A, TNFSF12, ARNT, MYC, JUN, FGF1	9.31 × 10 ^−6^

GO:0034142, Toll-like receptor 4 signaling pathway	ATF2, CDK1, MEF2C, FOS, RELA, JUN, ATF1, NFKBIA	9.31 × 10 ^−6^

GO:0030834, Regulation of actin filament depolymerization	SPTB, SPTBN1, SPTAN1, SPTA1, ADD1, EPB49	1.09 × 10 ^−5^

GO:0030837, Negative regulation of actin filament polymerization	SPTB, SPTBN1, SPTAN1, SPTA1, ADD1, EPB49	2.37 × 10 ^−5^

GO:0002224, Toll-like receptor signaling pathway	ATF2, CDK1, MEF2C, FOS, RELA, JUN, ATF1, NFKBIA	2.48 × 10 ^−5^

GO:0008629, Induction of apoptosis by intracellular signals	PML, BRCA1, YWHAB, TP53, BID, CDKN1A, RNF7, IFI16	2.92 × 10 ^−5^

GO:0050851, Antigen receptor-mediated signaling pathway	WAS, MEF2C, RELA, PSEN2, PTPRC, PAK1, PTEN, NFKBIA	8.55 × 10 ^−5^

GO:0002221, Pattern recognition receptor signaling pathway	ATF2, CDK1, MEF2C, FOS, RELA, JUN, ATF1, NFKBIA	9.15 × 10 ^−5^

GO:0001936, Regulation of endothelial cell proliferation	FGF2, AGGF1, CAV1, HIF1A, TNFSF12, ARNT, JUN	9.47 × 10 ^−5^

**MIMAT0000077(hsa-miR-22-3p)**		

**GO term**	**Genes**	**Adj. *p* -value**

GO:0035583, Negative regulation of transforming growth factor beta receptor signaling pathway by extracellular sequestering of TGFbeta	FBN2, FBN1, LTBP1	1.45 × 10 ^−5^

**MIMAT0000265(hsa-miR-204-5p)**		

**GO term**	**Genes**	**Adj. *p* -value**

GO:0035583, Negative regulation of transforming growth factor beta receptor signaling pathway by extracellular sequestering of TGFbeta	FBN1, FBN2, LTBP1	6.31 × 10 ^−5^

**Table S7:** GOBO survival analysis results. Genes which was annotated with the specified GO term of proteins in the PIN would be used as input gene set for GOBO analysis.

miRNA	GO term	*p*-value
MIMAT0000064(hsa-let-7c)	**GO:0015630, Microtubule cytoskeleton**	**9.97 × 10** ^−^ ^6^ ***
	GO:0043067, Regulation of programmed cell death	0.268067
	GO:0043069, Negative regulation of programmed cell death	0.0390439 *
	GO:0060548, Negative regulation of cell death	0.0390439 *

MIMAT0000076(hsa-miR-21-5p)	GO:0007173, Epidermal growth factor receptor signaling pathway	0.721139
	GO:0043067, Regulation of programmed cell death	0.266312

MIMAT0000077(hsa-miR-22-3p)	GO:0035583, Negative regulation of transforming growth factor beta receptor signaling pathway by extracellular sequestering of TGFbeta	0.940727

MIMAT0000089(hsa-miR-31-5p)	GO:0003376, Sphingosine-1-phosphate signaling pathway	0.062202
	**GO:0006917, Induction of apoptosis**	0.048584 *
	GO:0007169, Transmembrane receptor protein tyrosine kinase signaling pathway	0.050408
	GO:0007187, G-protein signaling, coupled to cyclic nucleotide second messenger	0.289466
	GO:0007188, G-protein signaling, coupled to cAMP nucleotide second messenger	0.687572
	GO:0010942, Positive regulation of cell death	0.356228
	**GO:0012502, Induction of programmed cell death**	0.048584 *
	GO:0019933, CAMP-mediated signaling	0.687572
	GO:0019935, Cyclic-nucleotide-mediated signaling	0.289466
	GO:0043067, Regulation of programmed cell death	0.694486
	GO:0043068, Positive regulation of programmed cell death	0.356228
	GO:0048011, Nerve growth factor receptor signaling pathway	0.154543

MIMAT0000250(hsa-miR-139-5p)	GO:0035583, Negative regulation of transforming growth factor beta receptor signaling pathway by extracellular sequestering of TGFbeta	0.940727

MIMAT0000265(hsa-miR-204-5p)	GO:0035583, Negative regulation of transforming growth factor beta receptor signaling pathway by extracellular sequestering of TGFbeta	0.940727

MIMAT0000423(hsa-miR-125b-5p)	GO:0002224, Toll-like receptor signaling pathway	0.380928
	GO:0002755, MyD88-dependent toll-like receptor signaling pathway	0.380928
	GO:0002756, MyD88-independent toll-like receptor signaling pathway	0.380928
	GO:0006916, Anti-apoptosis	0.0593
	GO:0006917, Induction of apoptosis	0.618064
	GO:0007169, Transmembrane receptor protein tyrosine kinase signaling pathway	0.776269
	GO:0008284, Positive regulation of cell proliferation	0.882324
	GO:0008285, Negative regulation of cell proliferation	0.883393
	GO:0008629, Induction of apoptosis by intracellular signals	0.073118
	GO:0010942, Positive regulation of cell death	0.972892
	GO:0012502, Induction of programmed cell death	0.618064
	GO:0015629, Actin cytoskeleton	0.596528
	**GO:0015630, Microtubule cytoskeleton**	0.028245 *
	GO:0030834, Regulation of actin filament depolymerization	0.654383
	GO:0030835, Negative regulation of actin filament depolymerization	0.654383
	GO:0030837, Negative regulation of actin filament polymerization	0.654383
	GO:0034130, Toll-like receptor 1 signaling pathway	0.380928
	GO:0034134, Toll-like receptor 2 signaling pathway	0.380928
	GO:0034138, Toll-like receptor 3 signaling pathway	0.380928
	GO:0034142, Toll-like receptor 4 signaling pathway	0.380928
	GO:0035666, TRIF-dependent toll-like receptor signaling pathway	0.380928
	GO:0043067, Regulation of programmed cell death	0.643418
	GO:0043068, Positive regulation of programmed cell death	0.972892
	GO:0043069, Negative regulation of programmed cell death	0.492576
	GO:0048011, Nerve growth factor receptor signaling pathway	0.171634
	**GO:0050678, Regulation of epithelial cell proliferation**	0.002205 **
	GO:0050679, Positive regulation of epithelial cell proliferation	0.205483
	GO:0051693, Actin filament capping	0.654383
	GO:0060548, Negative regulation of cell death	0.413665

MIMAT0000437(hsa-miR-145-5p)	GO:0007178, Transmembrane receptor protein serine/threonine kinase signaling pathway	0.196953
	GO:0030509, BMP signaling pathway	0.196953
	GO:0090092, Regulation of transmembrane receptor protein serine/threonine kinase signaling pathway	0.843529

MIMAT0000443(hsa-miR-125a-5p)	GO:0001936, Regulation of endothelial cell proliferation	0.115146
	GO:0002221, Pattern recognition receptor signaling pathway	0.380928
	GO:0002224, Toll-like receptor signaling pathway	0.380928
	GO:0002755, MyD88-dependent toll-like receptor signaling pathway	0.380928
	GO:0002756, MyD88-independent toll-like receptor signaling pathway	0.380928
	GO:0006916, Anti-apoptosis	0.0593
	GO:0006917, Induction of apoptosis	0.618064
	GO:0007169, Transmembrane receptor protein tyrosine kinase signaling pathway	0.776269
	GO:0008284, Positive regulation of cell proliferation	0.882324
	GO:0008285, Negative regulation of cell proliferation	0.883393
	GO:0008629, Induction of apoptosis by intracellular signals	0.073118
	GO:0010942, Positive regulation of cell death	0.972892
	GO:0012502, Induction of programmed cell death	0.618064
	GO:0015629, Actin cytoskeleton	0.596528
	**GO:0015630, Microtubule cytoskeleton**	0.028245 *
	GO:0030834, Regulation of actin filament depolymerization	0.654383
	GO:0030835, Negative regulation of actin filament depolymerization	0.654383
	GO:0030837, Negative regulation of actin filament polymerization	0.654383
	GO:0034130, Toll-like receptor 1 signaling pathway	0.380928
	GO:0034134, Toll-like receptor 2 signaling pathway	0.380928
	GO:0034138, Toll-like receptor 3 signaling pathway	0.380928
	GO:0034142, Toll-like receptor 4 signaling pathway	0.380928
	GO:0035666, TRIF-dependent toll-like receptor signaling pathway	0.380928
	GO:0043067, Regulation of programmed cell death	0.643418
	GO:0043068, Positive regulation of programmed cell death	0.972892
	GO:0043069, Negative regulation of programmed cell death	0.492576
	GO:0048011, Nerve growth factor receptor signaling pathway	0.171634
	**GO:0050678, Regulation of epithelial cell proliferation**	0.002205 **
	GO:0050679, Positive regulation of epithelial cell proliferation	0.205483
	GO:0050851, Antigen receptor-mediated signaling pathway	0.103325
	GO:0051693, Actin filament capping	0.654383
	GO:0060548, Negative regulation of cell death	0.413665

MIMAT0002820(hsa-miR-497-5p)	GO:0001959, Regulation of cytokine-mediated signaling pathway	0.06699
	GO:0006917, Induction of apoptosis	0.142401
	GO:0007169, Transmembrane receptor protein tyrosine kinase signaling pathway	0.837635
	GO:0007173, Epidermal growth factor receptor signaling pathway	0.387447
	GO:0007178, Transmembrane receptor protein serine/threonine kinase signaling pathway	0.240167
	**GO:0007179, Transforming growth factor beta receptor signaling pathway**	0.017876 *
	GO:0008284, Positive regulation of cell proliferation	0.430255
	GO:0008285, Negative regulation of cell proliferation	0.149994
	GO:0008543, Fibroblast growth factor receptor signaling pathway	0.521978
	GO:0010942, Positive regulation of cell death	0.237692
	GO:0012502, Induction of programmed cell death	0.142401
	GO:0015629, Actin cytoskeleton	0.228804
	GO:0015630, Microtubule cytoskeleton	0.18331
	GO:0017015, Regulation of transforming growth factor beta receptor signaling pathway	0.128773
	GO:0030509, BMP signaling pathway	0.39837
	GO:0030521, Androgen receptor signaling pathway	0.383811
	GO:0032956, Regulation of actin cytoskeleton organization	0.91762
	GO:0042058, Regulation of epidermal growth factor receptor signaling pathway	0.934045
	GO:0042059, Negative regulation of epidermal growth factor receptor signaling pathway	0.789492
	GO:0043067, Regulation of programmed cell death	0.111544
	GO:0043068, Positive regulation of programmed cell death	0.237692
	GO:0043069, Negative regulation of programmed cell death	0.856892
	GO:0048011, Nerve growth factor receptor signaling pathway	0.471986
	GO:0060548, Negative regulation of cell death	0.667437
	GO:0070302, Regulation of stress-activated protein kinase signaling cascade	0.561032
	GO:0090092, Regulation of transmembrane receptor protein serine/threonine kinase signaling pathway	0.182314

MIMAT0002856(hsa-miR-520d-3p)	GO:0006917, Induction of apoptosis	0.489781
	GO:0007169, Transmembrane receptor protein tyrosine kinase signaling pathway	0.171689
	GO:0007173, Epidermal growth factor receptor signaling pathway	0.449696
	GO:0008284, Positive regulation of cell proliferation	0.05916
	GO:0008286, Insulin receptor signaling pathway	0.237933
	GO:0008543, Fibroblast growth factor receptor signaling pathway	0.159318
	GO:0008629, Induction of apoptosis by intracellular signals	0.502822
	GO:0010942, Positive regulation of cell death	0.076906
	GO:0012502, Induction of programmed cell death	0.489781
	**GO:0015630, Microtubule cytoskeleton**	0.000292 ***
	GO:0042058, Regulation of epidermal growth factor receptor signaling pathway	0.762633
	GO:0042059, Negative regulation of epidermal growth factor receptor signaling pathway	0.826854
	GO:0043067, Regulation of programmed cell death	0.740092
	GO:0043068, Positive regulation of programmed cell death	0.076906
	GO:0043069, Negative regulation of programmed cell death	0.067499
	**GO:0048011, Nerve growth factor receptor signaling pathway**	0.014926 *
	**GO:0051988, Regulation of attachment of spindle microtubules to kinetochore**	**7.48 × 10** ^−^ ^6^ ***
	GO:0060548, Negative regulation of cell death	0.213035

* *p* < 0.05,

** *p* < 0.01;

*** *p* < 0.001.

**Table S8:** Pathophysiogical characteristics of miRNA array data used in ROC curve analysis.

Sample Name	ER	PR	HER	TNM	Stage	Grade
S621T1	0	0	0	pT1N0M0	I	2
S434T1	1	0	1	T2N1M0	IIB	3
S403T1	0	1	0	T2N1M0	IIB	2
S459T1	1	0	0	T4N0M1	IV	1
S455N1	1	0	0	T3N3M1	IV	3
S545T1	0	0	0	pT2N0M0	IIA	3
S173N1	0	0	1	T2N3M0	IIIC	3
S363T1	1	1	0	T2N1M0	IIB	1
S909T1	1	1	1	pT3N3aM0	IIIC	(Unknown)
S645T1	0	1	1	pT1bN0M0	I	3
S898T1	1	0	0	pT2N0(i-)M0	IIA	(Unknown)
S201T1	1	0	0	T1N1M0	IIA	2
S631T1	1	1	1	T2N3aM1	IV	2
S303T1	0	0	1	T2N0M0	IIA	2
S502T1	0	0	0	pT3N0M0	IIB	3
S498N1	1	1	1	pT1cN1aM0	IIA	2
S536T1	1	0	1	T1cN1miM0	IIA	2
S660T1	0	0	1	T1N0M0	I	3
S358N1	0	0	1	T2pN2M0	IIIA	2
S665T1	0	0	0	T2N3M0	IIIC	3
S475T1	0	0	1	pT1cN0M0	I	3
S423T1	1	1	0	T2N3M0	IIIC	1
S507T1	0	0	0	pT1cN1aM0	IIA	3
S891T1	1	0	0	pT2NxM0	IIA	2
S422T1	1	1	0	T2N0M0	IIA	1
S961T1	0	0	1	T2N2aM0	IIIA	2
S622T1	0	0	0	pT2N0M0	IIA	2
S454T1	0	0	0	T1cN0M0	I	2
S433T1	1	0	0	T2N0M0	IIA	1
S673T1	0	0	0	pT2N0M0	IIA	2
S450T1	0	0	1	T2N1M0	IIB	3
S430T1	1	1	0	T2N3M0	IIIC	2
S437T1	1	0	0	T3N1M0	IIIA	3
S574T1	0	0	0	T2N0M0	IIA	2
S401T1	1	1	0	T1N0M0	I	1
S427N1	0	0	0	T3N1M0	IIIA	2
S894T1	0	0	0	pT1cN0M0	I	2
S929T1	1	0	0	pT2N0M0	IIA	2
S173T1	0	0	1	T2N3M0	IIIC	3
S622N1	0	0	0	pT2N0M0	IIA	2
S562T1	1	1	1	pT1aN0M0	I	3
S602T1	0	0	0	pT1cN0M0	I	3
S490T1	1	1	1	pT2N0M0	IIA	2
S677T1	0	0	0	pT2N1M0	IIB	3
S881T1	0	0	0	pT2N1aM0	IIB	3
S619T1	0	0	0	pT2N0M0	IIA	3
S446T1	0	0	0	T2N2M0	IIIA	3
S446T2	0	0	0	T2N2M0	IIIA	3
S453T1	1	1	1	T2N1M0	IIB	2
S562N1	1	1	1	pT1aN0M0	I	3
S557T1	0	0	0	pT2N0M0	IIA	3
S594T1	1	1	1	pT2N3aM0	IIIC	3
S582T1	0	0	0	pT2N0M0	IIA	3
S358T1	0	0	1	T2pN2M0	IIIA	2
S368T1	0	0	1	T3N3M0	IIIC	2
S175T1	1	1	0	T3N3Mx	IIIC	3
S357T1	0	0	0	T2pN1M0	IIIC	3
S653T1	0	0	0	pT3N3aM0	IIIC	2
S722T1	1	1	1	pT1N0M0	I	3
S593T1	0	0	0	pT1N0M0	I	3
S543T1	1	1	1	pT2N1M0	IIB	2
S498T1	1	1	1	pT1cN1aM0	IIA	2
S389T1	1	0	0	T2N1M0	IIB	3
S614T1	0	1	1	TxN0M1	IIIA	(Unknown)
S536N1	1	0	1	T1cN1miM0	IIA	2
S462T1	1	1	0	T3N3M0	IIIC	2
S477T1	0	0	0	pT1cN0M0	I	3
S917T1	0	0	0	T2N0M0	IIA	(Unknown)
S213T1	0	0	1	T1cN1M0	IIA	3
S628T1	0	0	0	T1N0M0	I	2
S291T1	0	0	0	T3pN2M0	IIIA	3
S593N1	0	0	0	pT1N0M0	I	3
S418T1	1	1	0	T2N3M0	IIIC	2
S420T1	1	1	0	T1N0M0	I	2
S363N1	1	1	0	T2N1M0	IIB	1
S629T1	1	0	1	pT1N0M0	I	2
S586T1	1	1	1	pT2N3aM0	IIIC	2
S415T1	0	1	0	T2N2M0	IIIA	2
S439T1	1	1	0	T2N2M0	IIIA	2
S941N1	0	0	0	pT1cN1aM0	IIA	3
S893T1	0	0	0	pT2N1micM0	IIB	3
S380T1	1	0	0	T2N1M0	IIB	2
S906N1	1	1	1	pT3N3aM0	IIIC	2
S918T1	0	0	0	pT2N0M0	IIA	3
S400T1	0	0	1	T2N0M0	IIA	2
S328T1	0	0	0	T1cpN0M0	I	3
S367T1	1	0	0	T1N1M0	IIIA	1
S420N1	1	1	0	T1N0M0	I	2
S922T1	0	0	0	pT1cN0(i-)M0	I	3
S896T1	0	0	1	pT2N1aM0	IIB	2
S410T1	0	1	1	T1N0M0	I	3
S572T1	0	0	1	T4N1aM0	IIIC	3
S448T1	0	0	0	T1N0M0	I	3
S207T1	0	0	1	T2N0M0	IIA	3
S604T1	0	0	1	pT2N1M0	IIB	3
S379T1	0	0	1	T2N2M0	IIIA	2
S906T1	1	1	1	pT3N3aM0	IIIC	2
S941T2	0	0	0	pT1cN1aM0	IIA	3
S941T1	0	0	0	pT1cN1aM0	IIA	3
S375T1	1	1	0	T1N0M0	I	2
S427T1	0	0	0	T3N1M0	IIIA	2
S417T1	0	0	0	pT2N0M0	IIA	3
S180T1	1	0	0	T4NxM0	IIIC	
S455T1	1	0	0	T3N3M1	IV	
S887T1	0	0	0	pT2N0M0	IIA	
S445T1	1	0	1	T2N1M0	IIB	
S469T1	1	1	0	T2N0M0	IIA	
S469T2	1	1	0	T2N0M0	IIA	
S483T1	1	1	0	T2N3M0	IIIC	
S444T1	1	1	0	T1NxM0	(Unknown)	
S909N1	1	1	1	pT3N3aM0	IIIC	
S464T1	1	1	0	T1N0M0	I	
S894N1	0	0	0	pT1cN0M0	I	
S698T1	1	1	1	pT1cN0M0	I	
S452T1	0	0	0	T1N0M0	I	
S474T1	(Unknown)	(Unknown)	(Unknown)	(Unknown)	(Unknown)	

## Supplementary Information



## Figures and Tables

**Figure 3:**
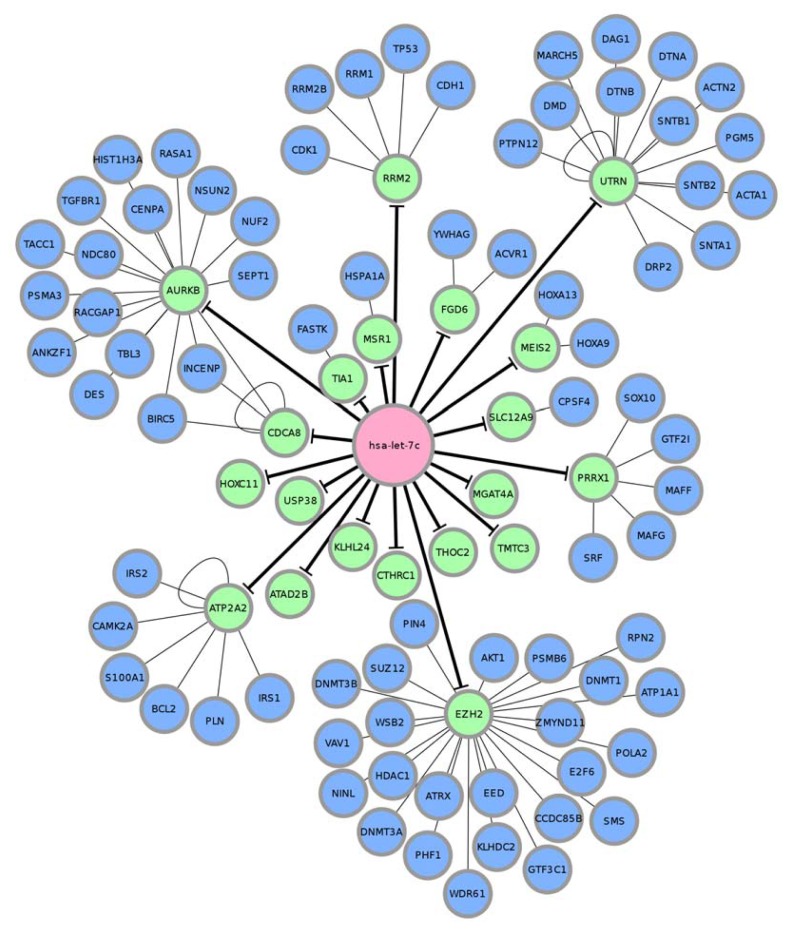
The let-7c-regulated protein interaction network (PIN). This is one of the 18 miRNA-regulated PINs constructed in this work. Figures of all other miRNA-regulated PINs are displayed in Supplementary Figures S2–S13.

**Table 1:** Selected enriched functions of let-7c. Member genes of the let-7c-regulated network annotated with corresponding enriched functions are listed.

MIMAT0000064(hsa-let-7c)		
GO term	Genes	Adj. *p*-value
GO:0043067, Regulation of programmed cell death	RRM2B, ACVR1, TP53, RASA1, TGFBR1, PSMA3, BIRC5, ACTN2, HOXA13, IRS2, FASTK, VAV1, PSMB6, BCL2, CDK1, HDAC1, SOX10, TIA1, AKT1, AURKB	3.43 × 10^−8^
GO:0043069, Negative regulation of programmed cell death	RRM2B, ACVR1, TP53, RASA1, TGFBR1, BIRC5, IRS2, BCL2, CDK1, HDAC1, SOX10, AKT1, AURKB	6.58 × 10^−6^
GO:0060548, Negative regulation of cell death	RRM2B, ACVR1, TP53, RASA1, TGFBR1, BIRC5, IRS2, BCL2, CDK1, HDAC1, SOX10, AKT1, AURKB	8.92 × 10^−6^
GO:0015630, Microtubule cytoskeleton	INCENP, SNTB2, SEPT1, TACC1, BIRC5, RACGAP1, PIN4, CDCA8, CDK1, PHF1, AKT1, AURKB, NINL, CCDC85B	5.15 × 10^−5^
