# Supplementary material for: MicroRNA-Regulated Protein-Protein Interaction Networks and Their Functions in Breast Cancer
Source: Int J Mol Sci. 2013 May 30;14(6):11560–606. doi: 10.3390/ijms140611560 (PMC3709748; doi:10.3390/ijms140611560)
Supplement: Supplementary file 1 [file ijms-14-11560-s001.pdf]

# Supplementary Information

**Figure S1.** ROC curves and expression distribution of miRNAs. Expression profiles of NTUH miRNA dataset was used for validating our results. Solid line: actual prediction performance of the miRNA as diagnostic marker; dashed line: AUC = 0.5 (no predictive power). ***** *p*-value < 0.05; ****** *p*-value < 0.01; ******* *p*-value < 0.001.

**A. hsa-let-7c. MIMAT0000064. *****

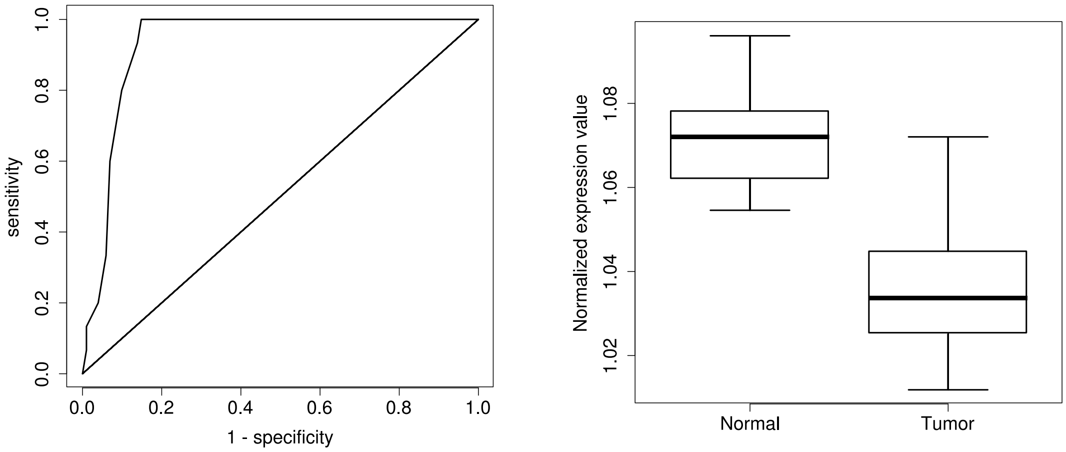


**B. hsa-miR-21-5p. MIMAT0000076. *****

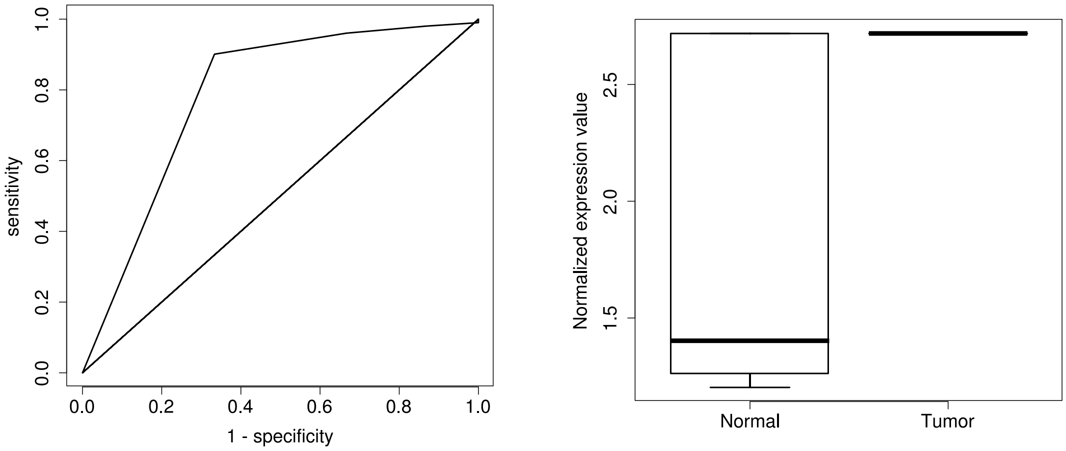


**C. hsa-miR-22-3p. MIMAT0000077.**

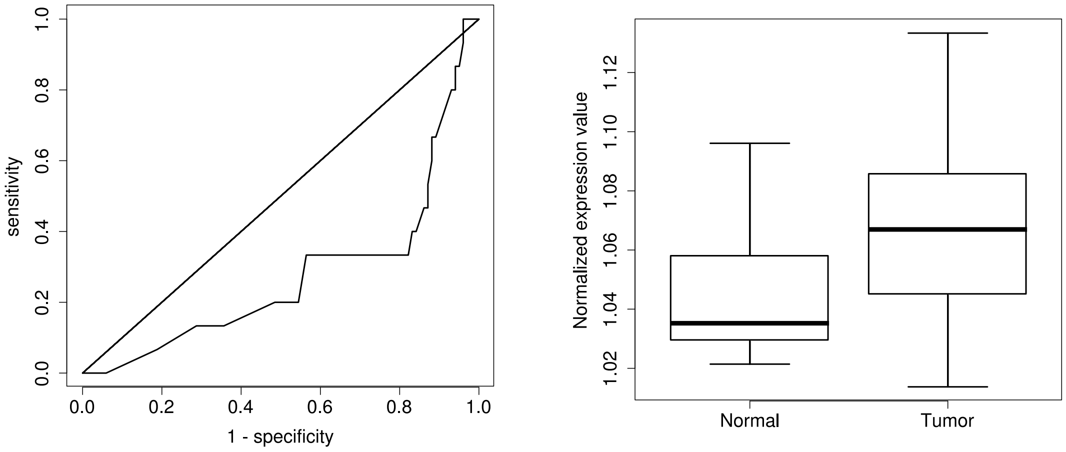

**Figure S1.** *Cont.*

**D. hsa-miR-31-5p. MIMAT0000089. *****

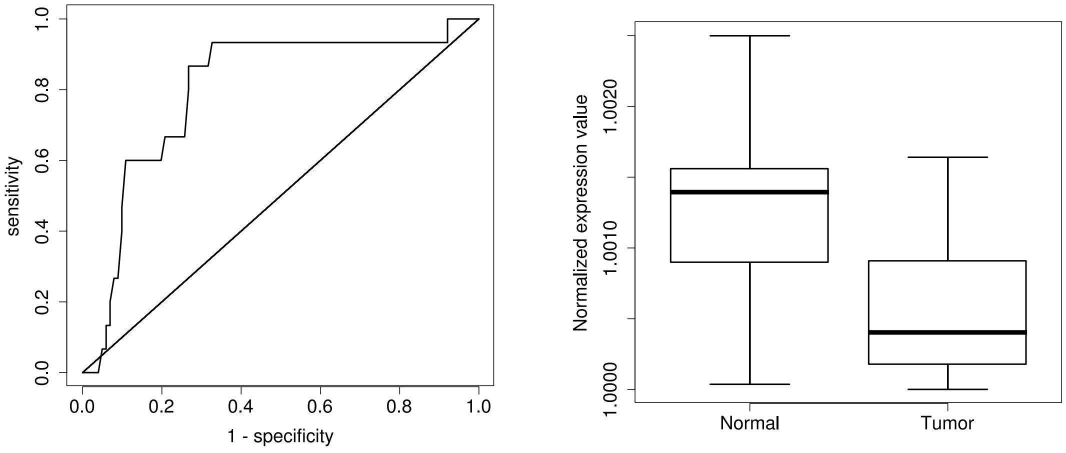


**E. hsa-miR-99a-5p. MIMAT0000097. *****

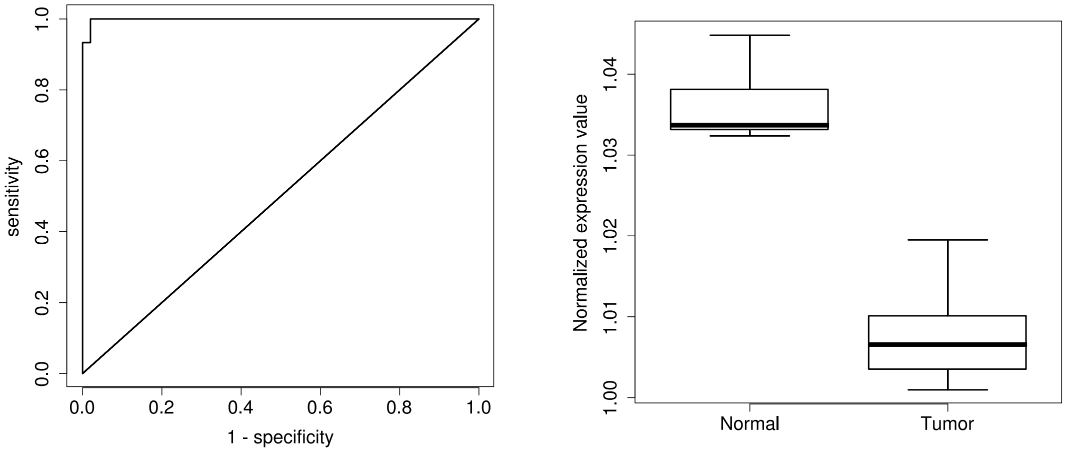


**F. hsa-miR-100-5p. MIMAT0000098. *****

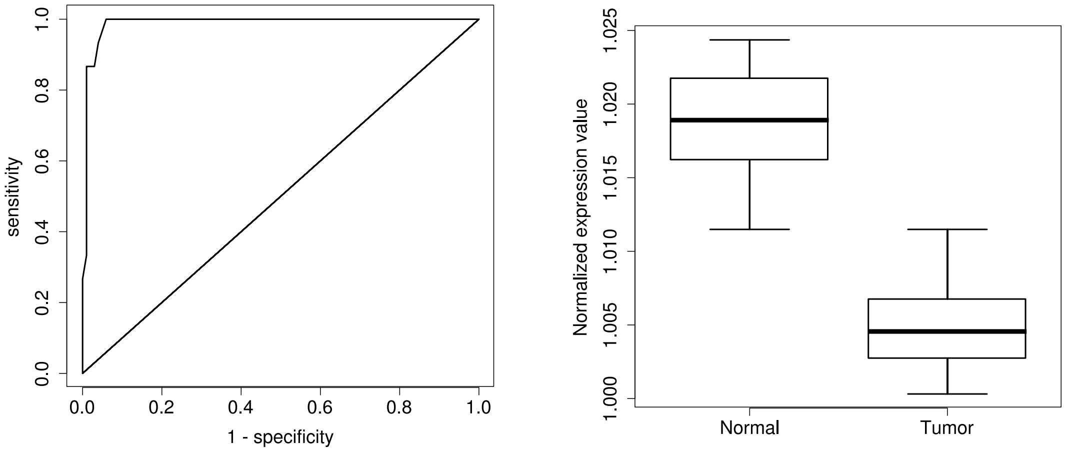

**Figure S1.** *Cont.*

**G. hsa-miR-139-5p. MIMAT0000250. *****

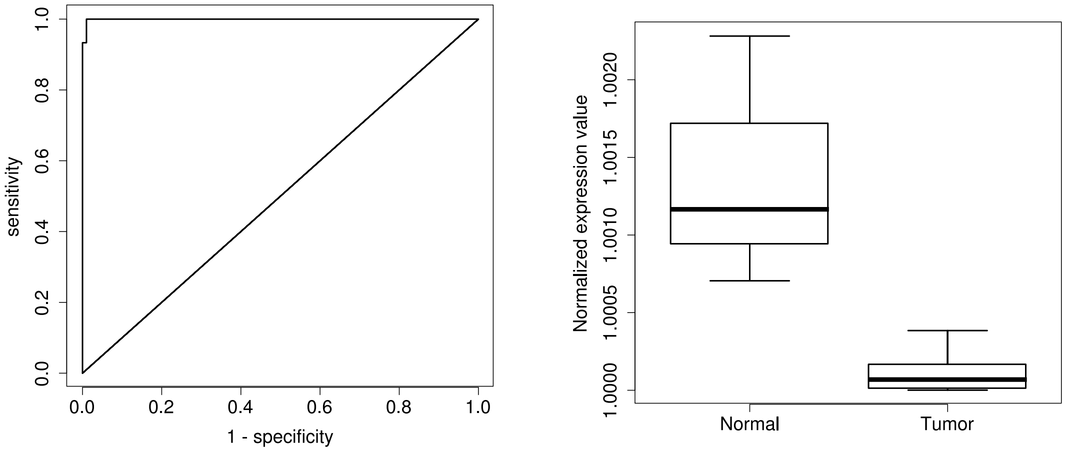


**H. hsa-miR-204-5p. MIMAT0000265. *****

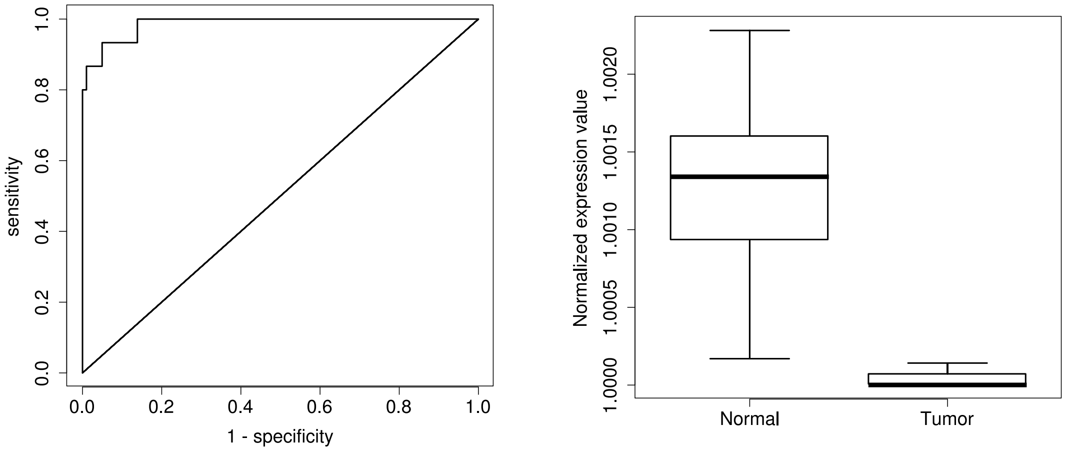


**I. hsa-miR-214-3p. MIMAT0000271. ***

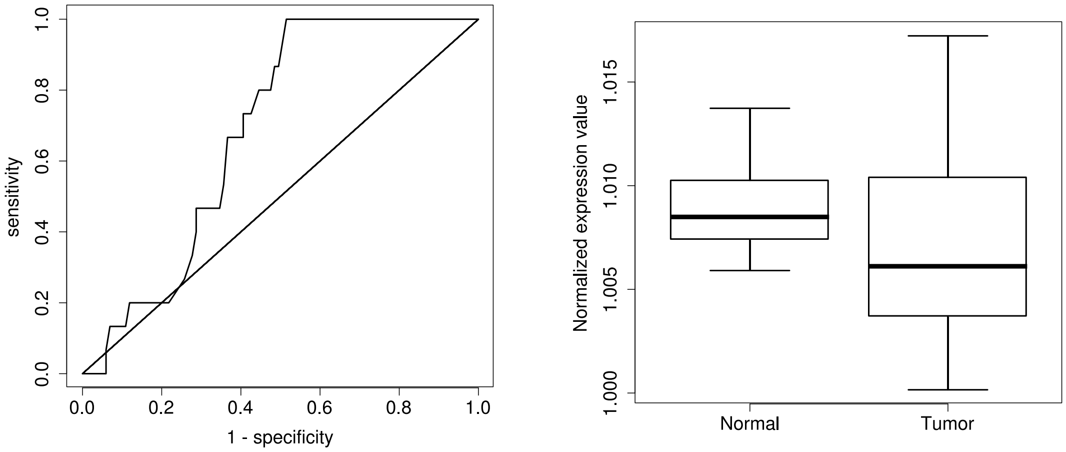

**Figure S1.** *Cont.*

**J. hsa-miR-215. MIMAT0000272.**

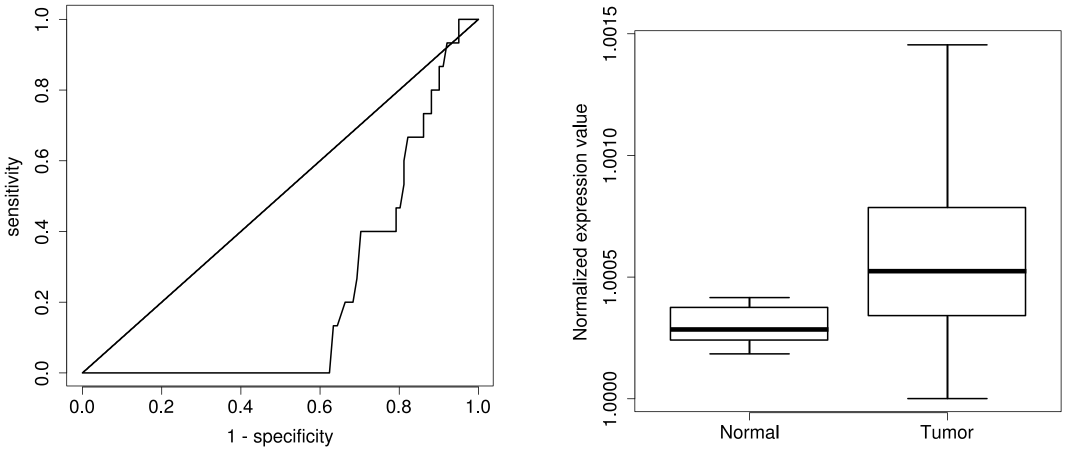


**K. hsa-miR-122-5p. MIMAT0000421.**

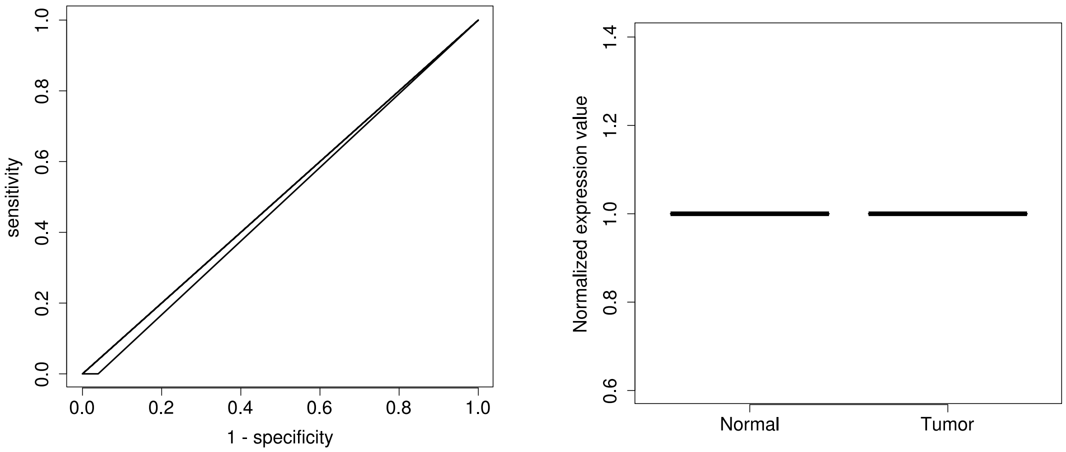


**L. hsa-miR-125b-5p. MIMAT0000423. *****

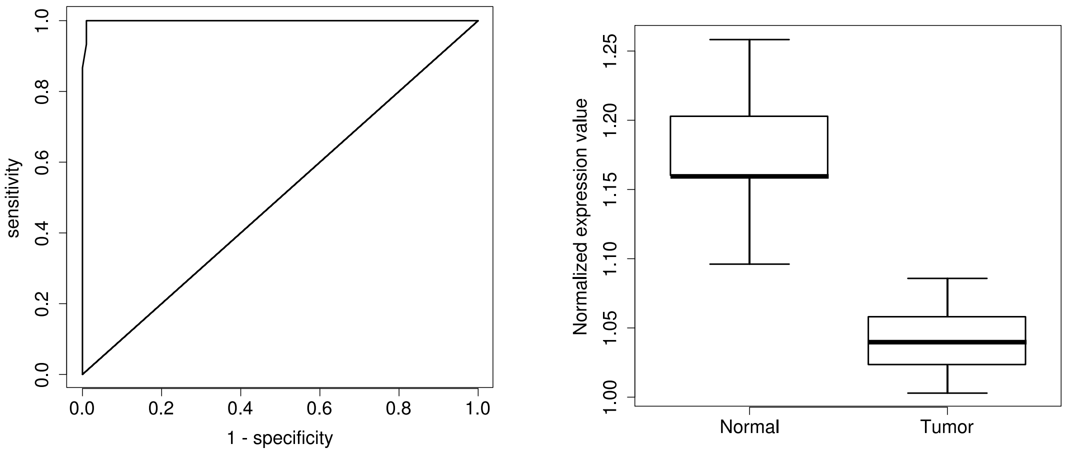

**Figure S1.** *Cont.*

**M. hsa-miR-145-5p. MIMAT0000437. *****

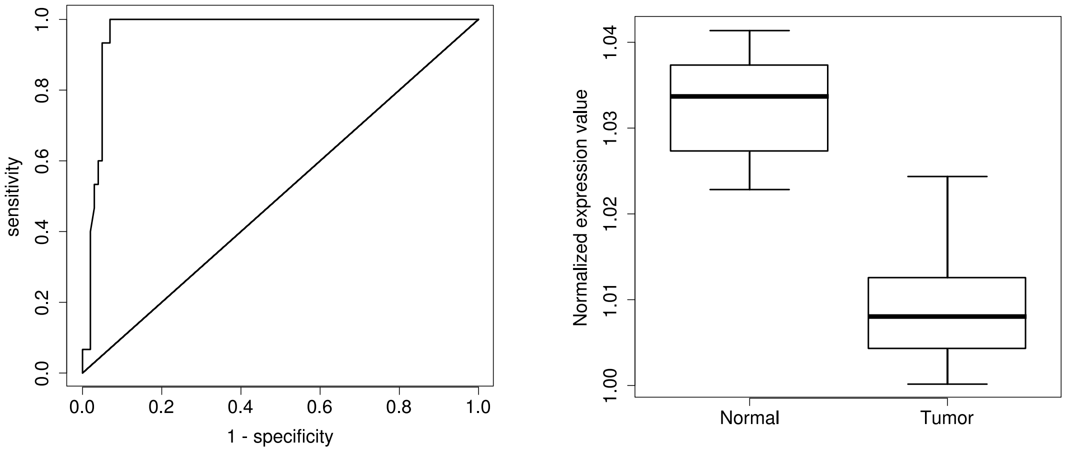


**N. hsa-miR-125a-5p. MIMAT0000443.**

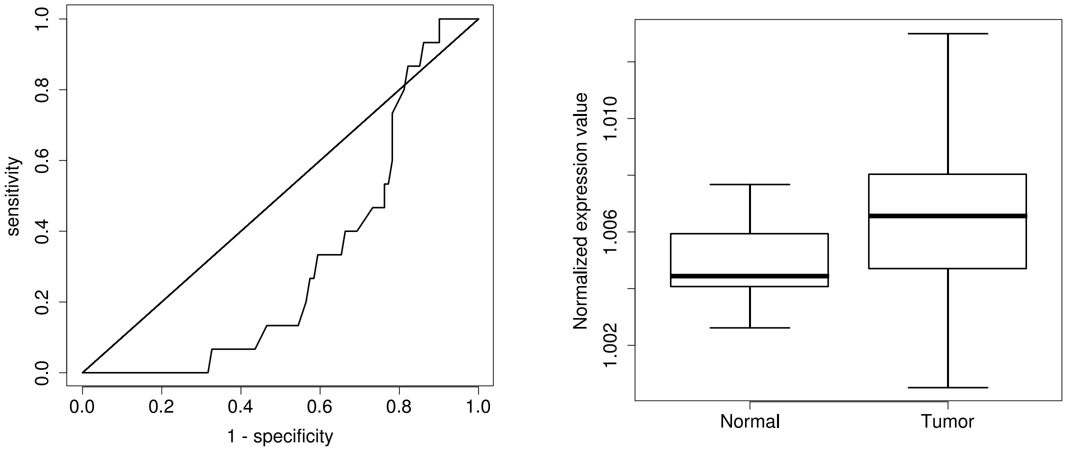


**O. hsa-miR-383. MIMAT0000738.**

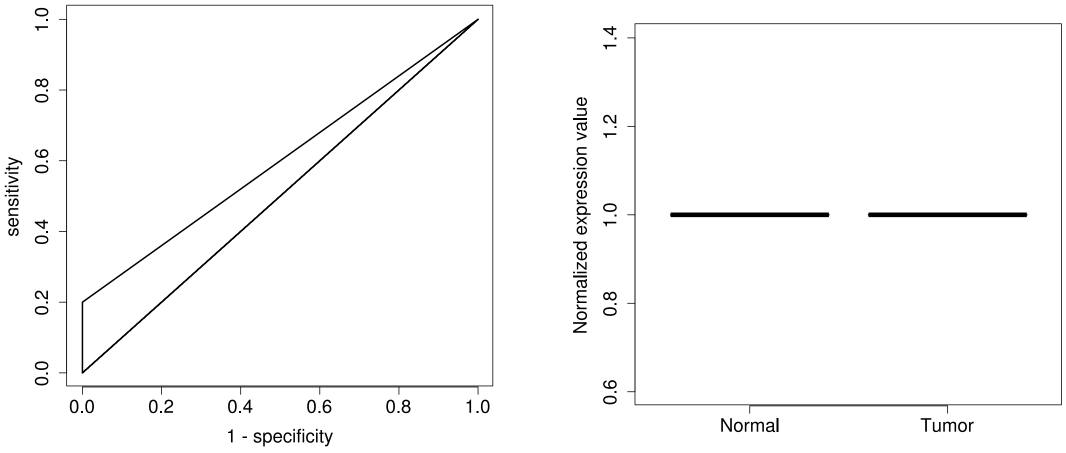

**Figure S1.** *Cont.*

**P. hsa-miR-193b-3p. MIMAT0002819.**

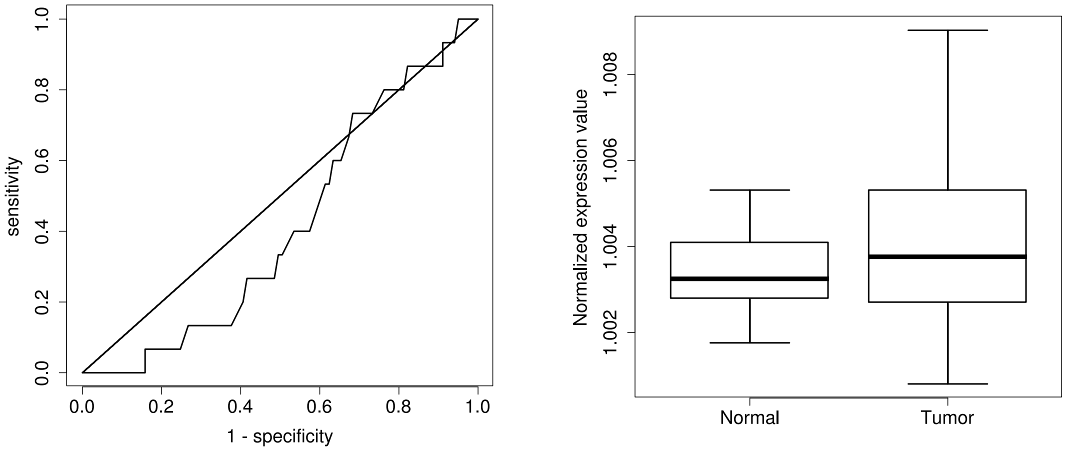


**Q. hsa-miR-497-5p. MIMAT0002820. *****

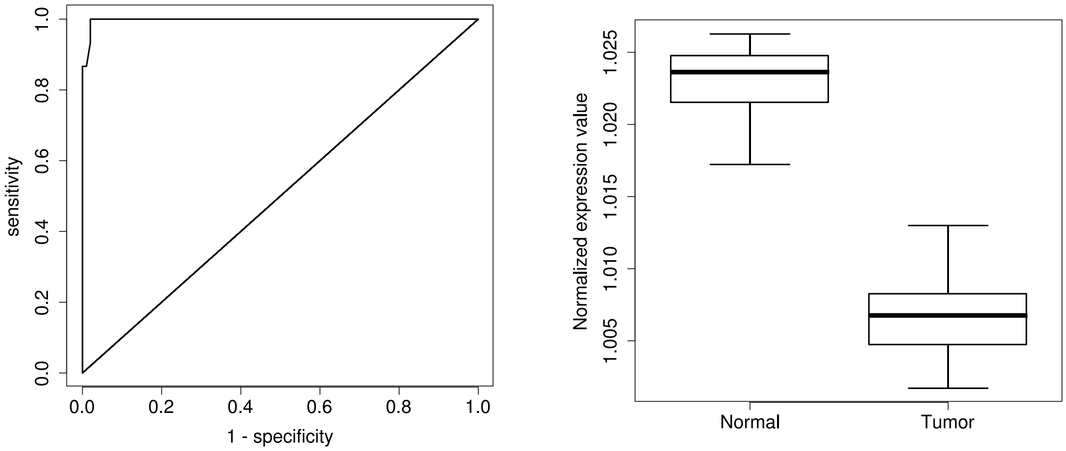


**R. hsa-miR-520d-3p. MIMAT0002856.**

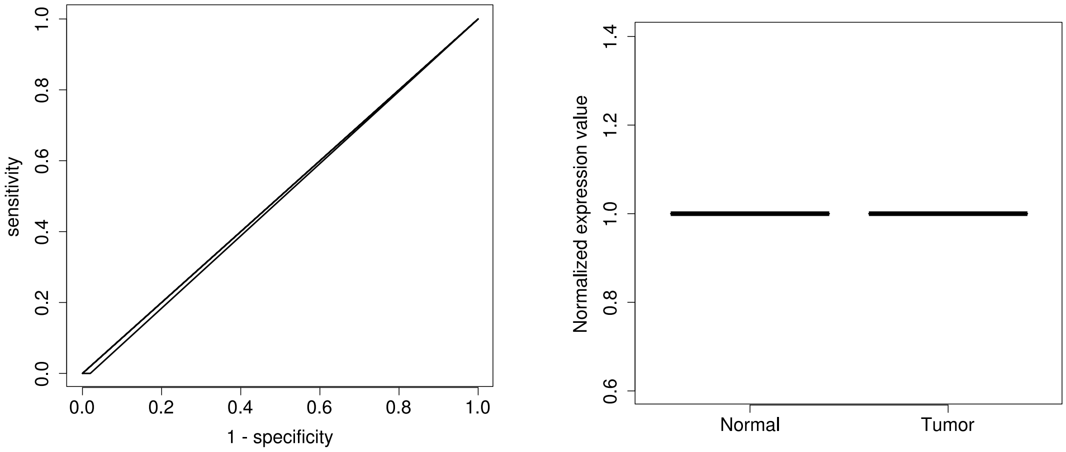

**Figure S2.** miRNA-regulated PIN. (hsa-miR-21-5p). **Red**: miRNA; **green**: direct target of the miRNA; **blue**: proteins interacting with direct target of the miRNA.

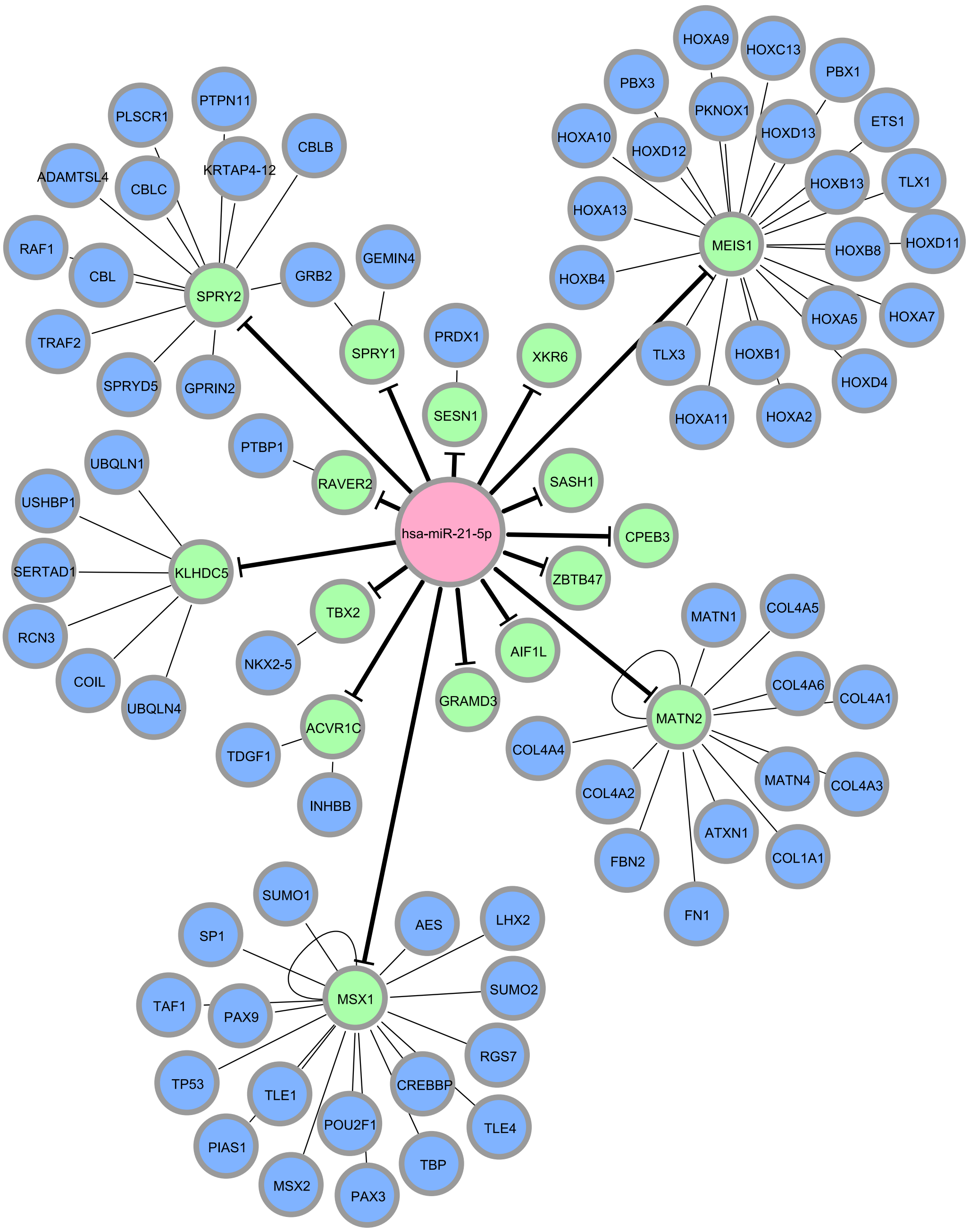

**Figure S3.** miRNA-regulate PIN. (hsa-miR-22-3p). **Red**: miRNA; **green**: direct target of the miRNA; **blue**: proteins interacting with direct target of the miRNA.

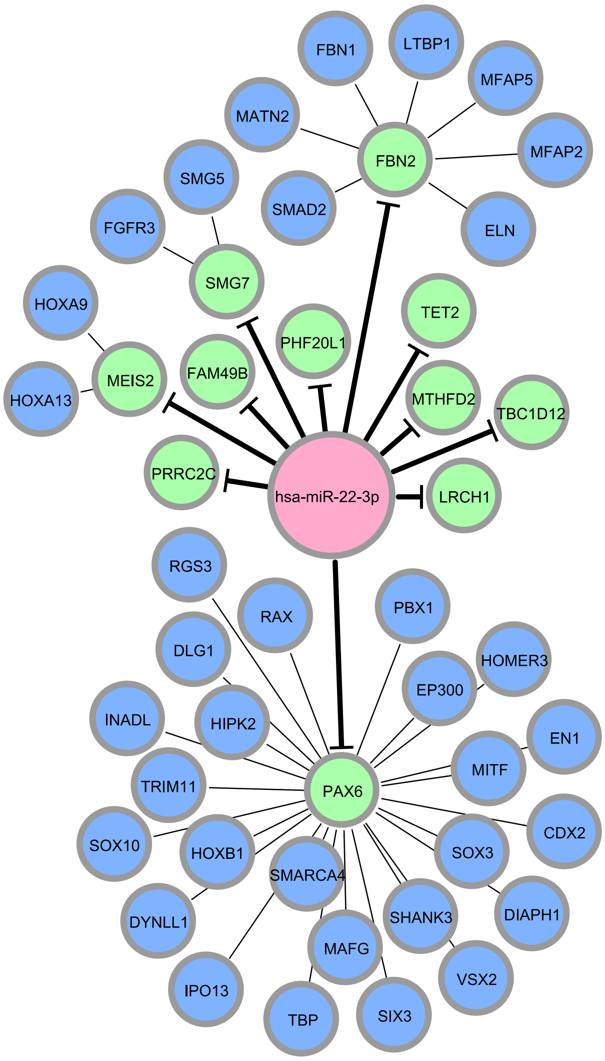


**Figure S4.** miRNA-regulated PINs. (hsa-miR-31-5p and hsa-miR-99a-5p). **Red**: miRNA; **green**: direct target of the miRNA; **blue**: proteins interacting with direct target of the miRNA.

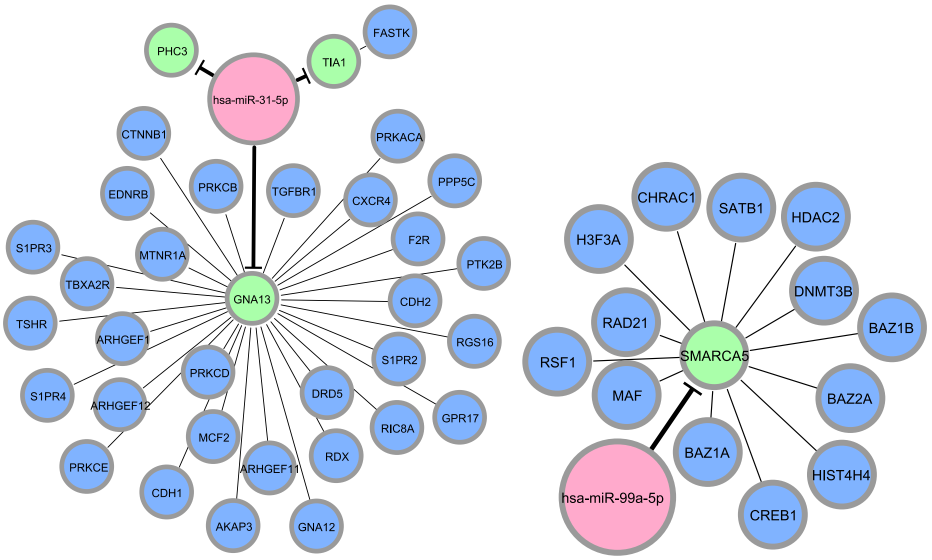

**Figure S5.** miRNA-regulated PINs. (hsa-miR-100-5p and hsa-miR-139-5p). **Red**: miRNA; **green**: direct target of the miRNA; **blue**: proteins interacting with direct target of the miRNA.

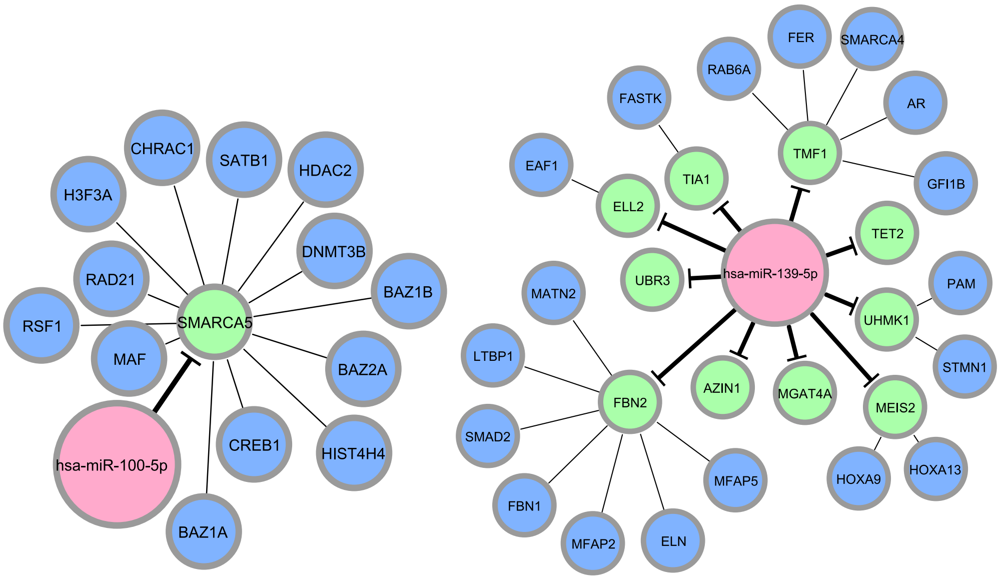


**Figure S6.** miRNA-regulated PIN. (hsa-204-5p). **Red**: miRNA; **green**: direct target of the miRNA; **blue**: proteins interacting with direct target of the miRNA.

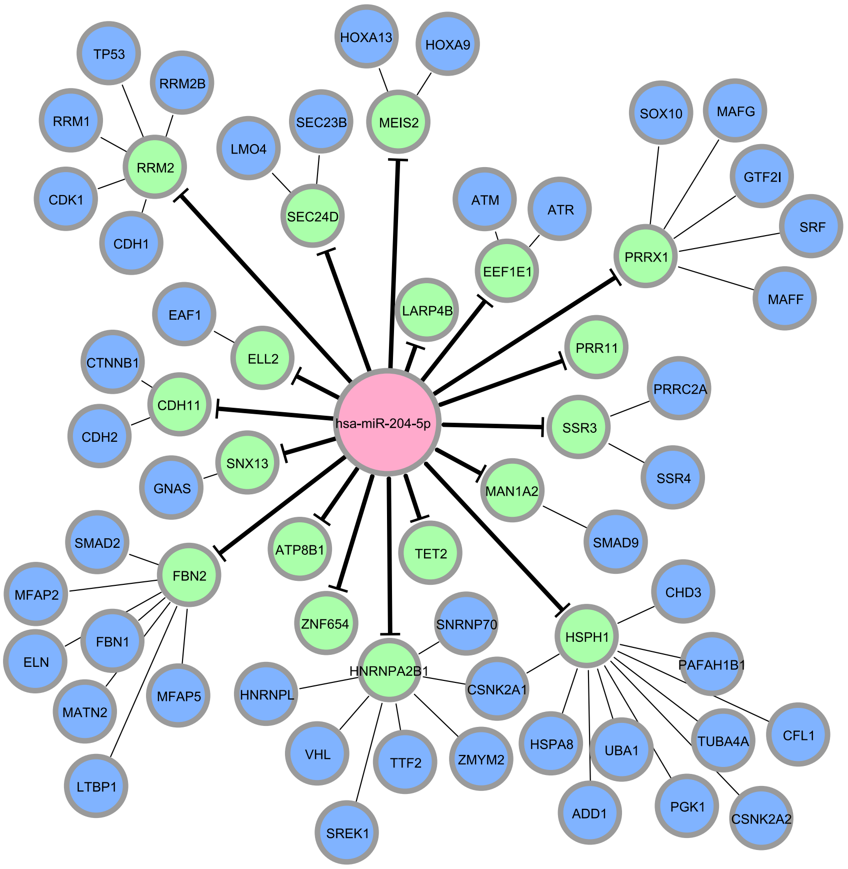

**Figure S7**. miRNA-regulated PINs. (hsa-miR-214-3p, hsa-miR-215, and hsa-miR-122-5p). **Red**: miRNA; **green**: direct target of the miRNA; **blue**: proteins interacting with direct target of the miRNA.

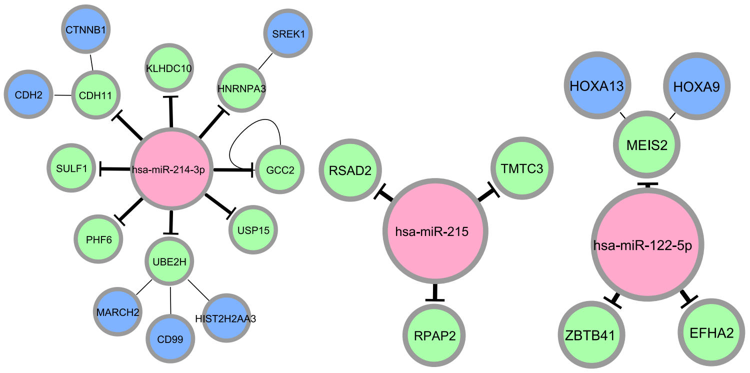


**Figure S8**. miRNA-regulated PIN. (hsa-miR-125b-5p). **Red**: miRNA; **green**: direct target of the miRNA; **blue**: proteins interacting with direct target of the miRNA.

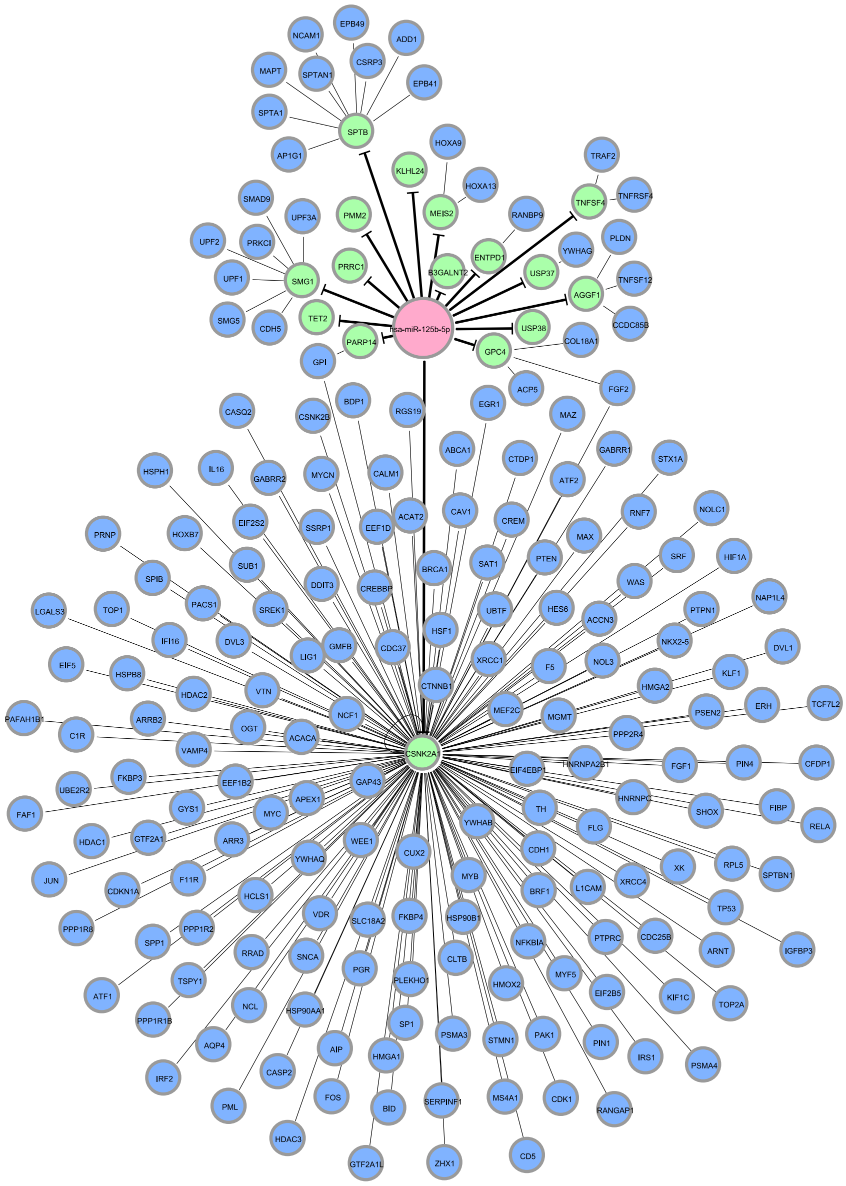

**Figure S9.** miRNA-regulated PIN. (hsa-miR-145-5p). **Red**: miRNA; **green**: direct target of the miRNA; **blue**: proteins interacting with direct target of the miRNA.

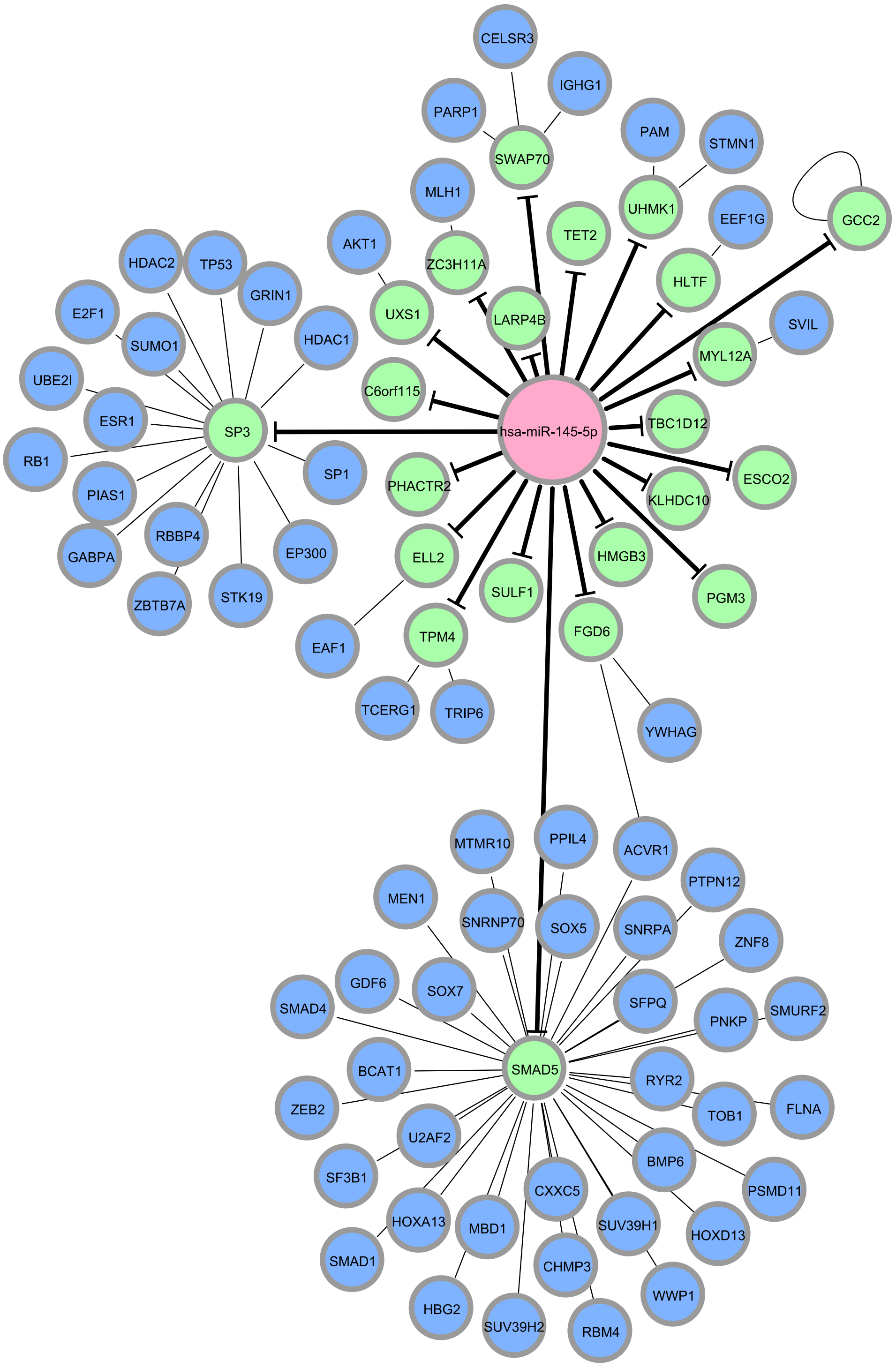

**Figure S10**. miRNA-regulated PIN. (hsa-miR-125a-5p). **Red**: miRNA; **green**: direct target of the miRNA; **blue**: proteins interacting with direct target of the miRNA.

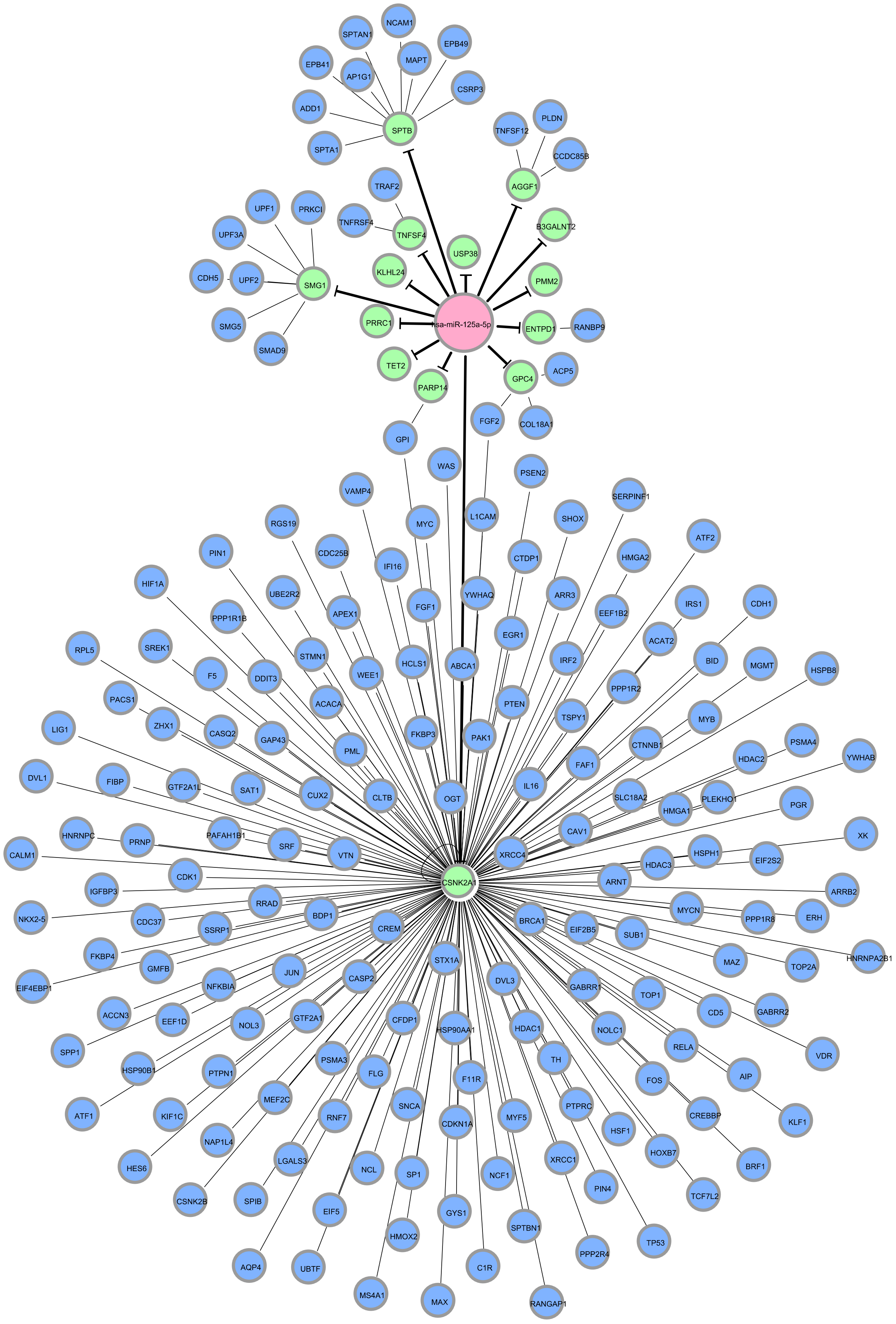

**Figure S11.** miRNA-regulated PINs. (hsa-miR-383 and hsa-miR-193b-3p). **Red**: miRNA; **green**: direct target of the miRNA; **blue**: proteins interacting with direct target of the miRNA.

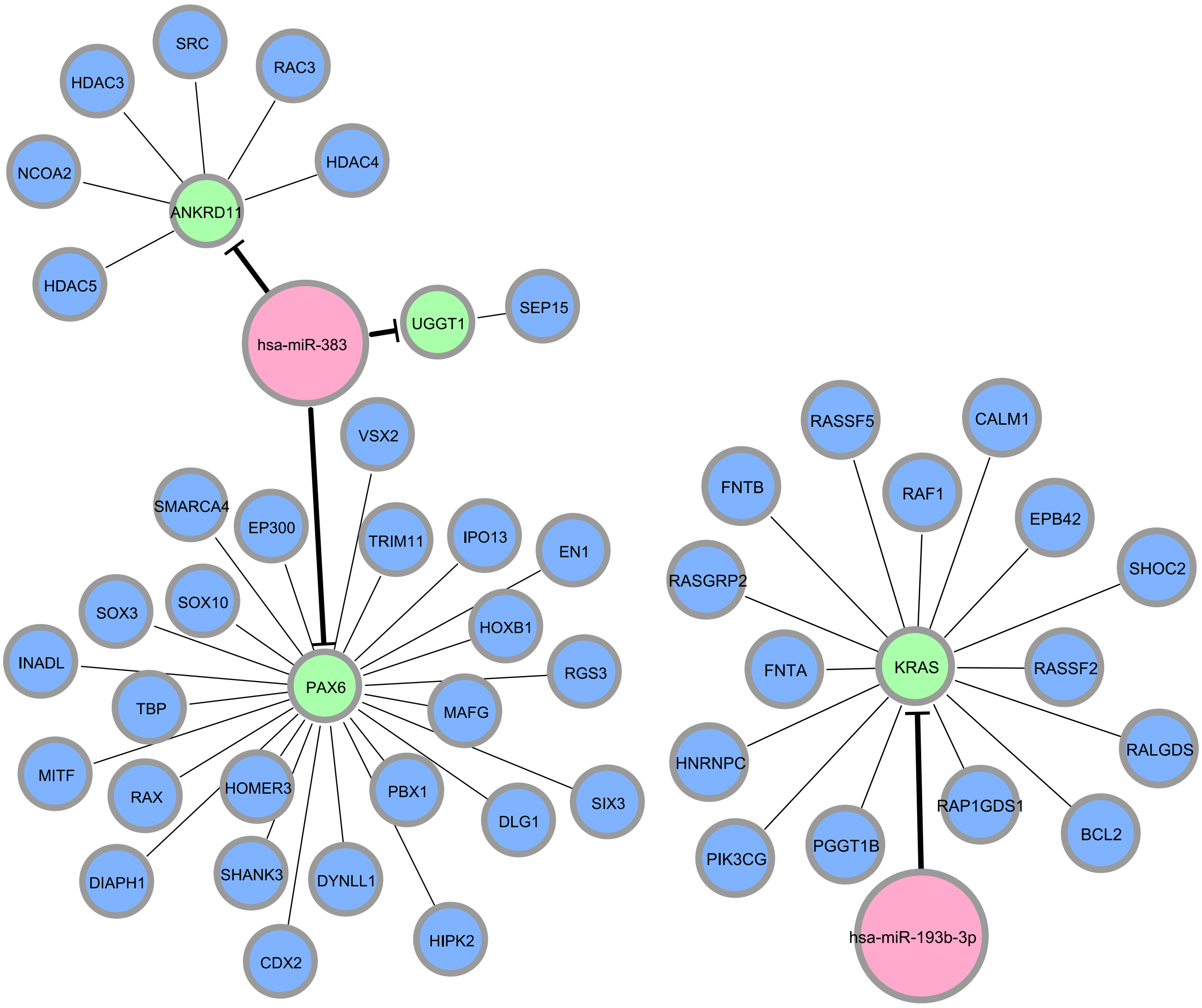

**Figure S12.** miRNA-regulated PIN. (hsa-miR-497-5p). **Red**: miRNA; **green**: direct target of the miRNA; **blue**: proteins interacting with direct target of the miRNA.

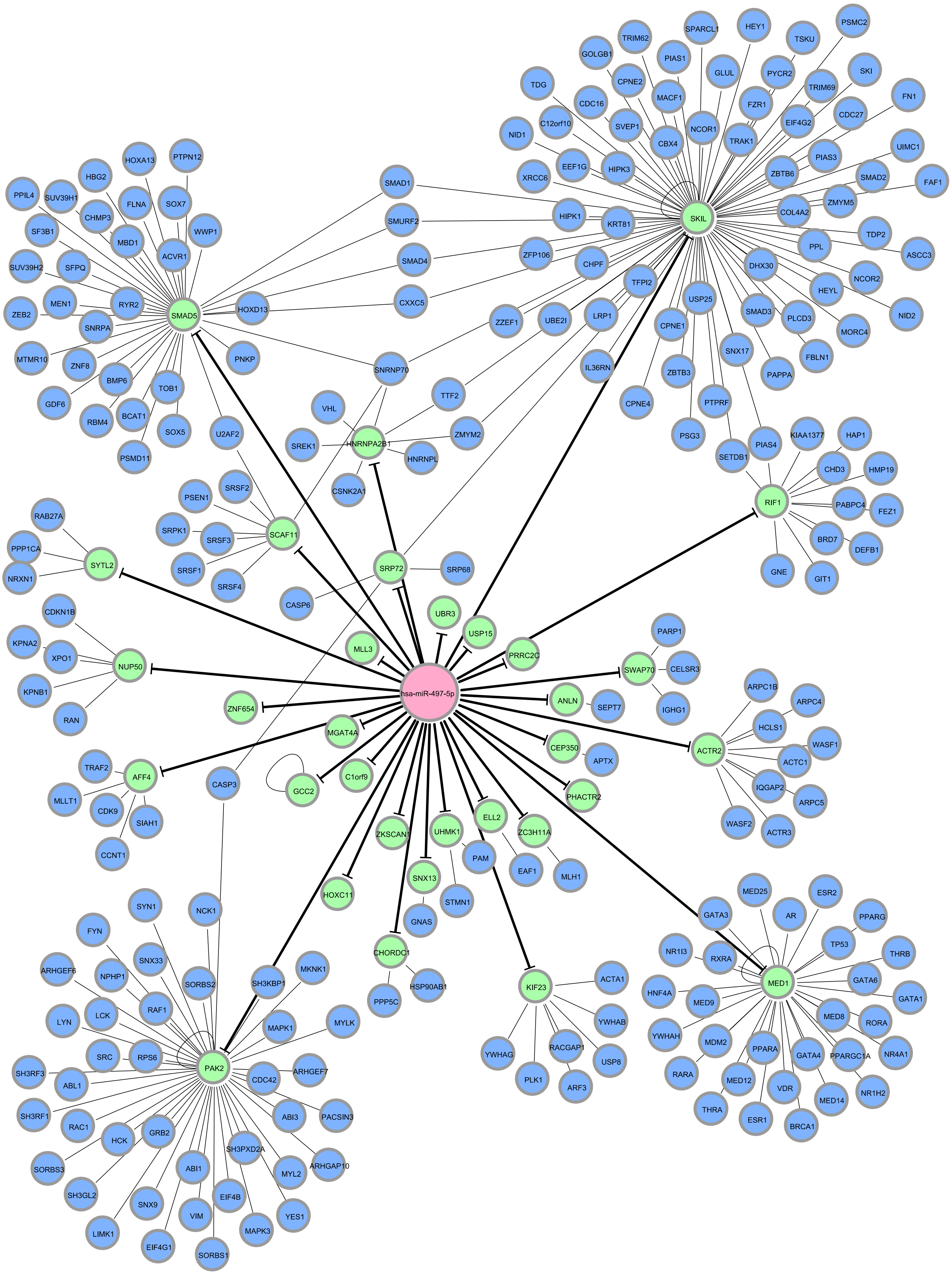

**Figure S13.** miRNA-regulated PIN. (hsa-miR-520d-3p). **Red**: miRNA; **green**: direct target of the miRNA; **blue**: proteins interacting with direct target of the miRNA.

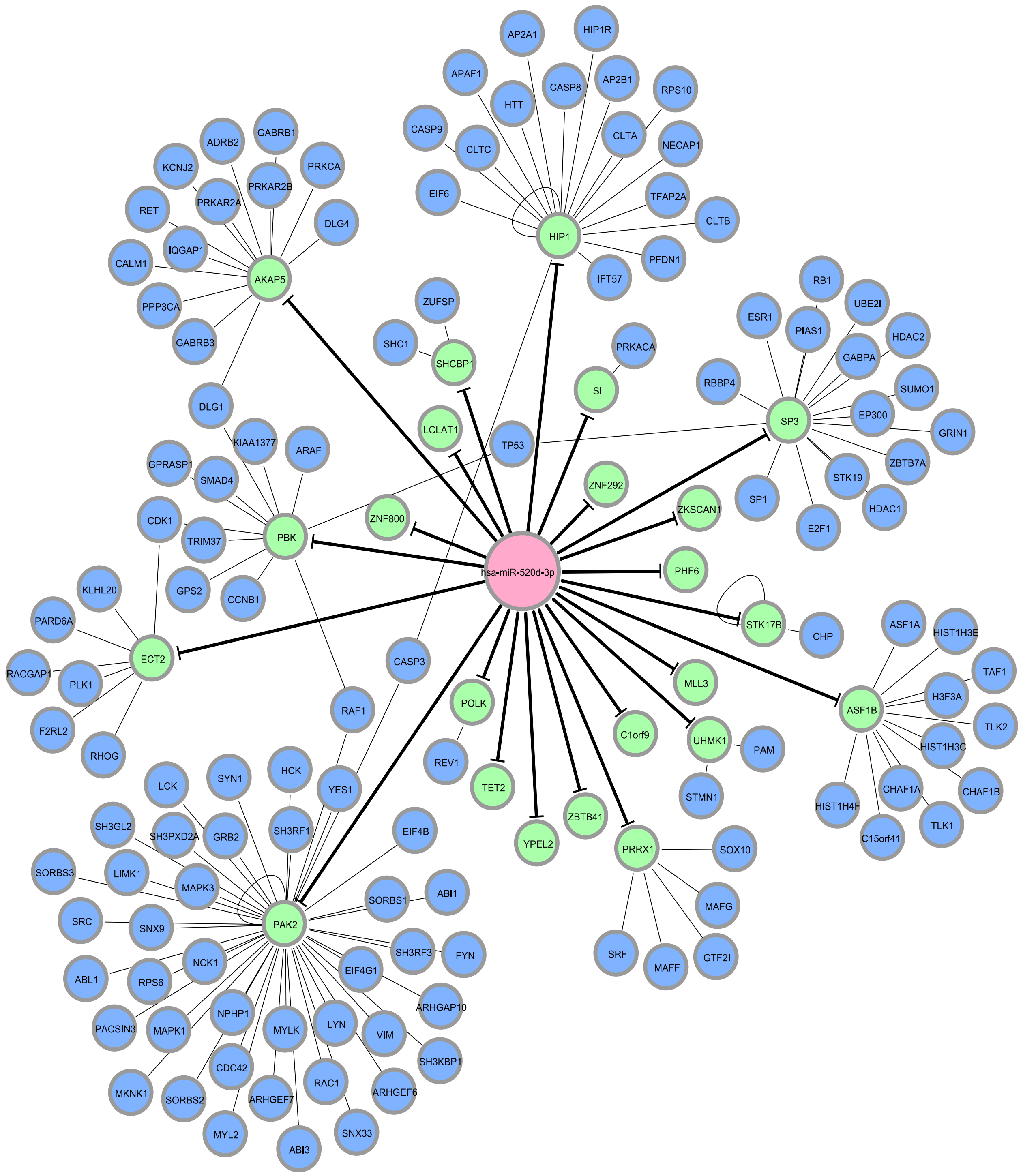

**Figure S14.** GOBO Kaplan-Meier survival curve of selected gene set with significant clinical outcome. (continue). The gene set was formed by selecting genes annotated with designated GO term in the miRNA-regulated PIN. Full GOBO Kaplan-Meier survival p-value were calculated and listed in Table 9. * $p < 0.05$; ** $p < 0.01$; *** $p < 0.001$.

**MIMAT0000064 (hsa-let-7c)*****

GO:0015630, Microtubule cytoskeleton

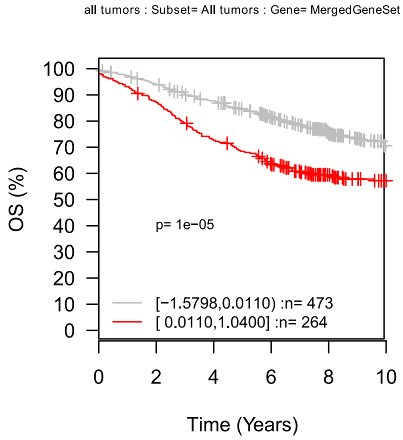


**MIMAT0000064 (hsa-let-7c)***

GO:0043069, Negative regulation of programmed cell death

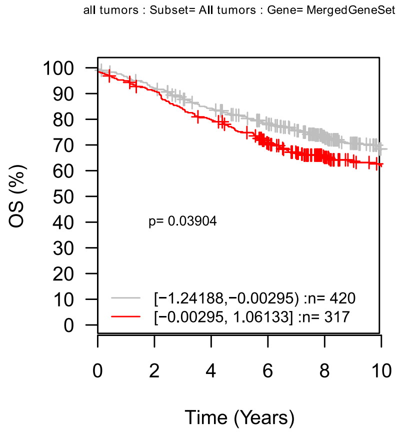


**MIMAT0000064 (hsa-let-7c)***

GO:0060548, Negative regulation of cell death

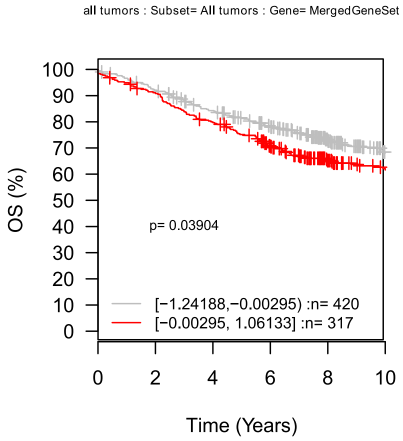


**MIMAT0000089 (hsa-miR-31-5p)***

GO:0006917, Induction of apoptosis

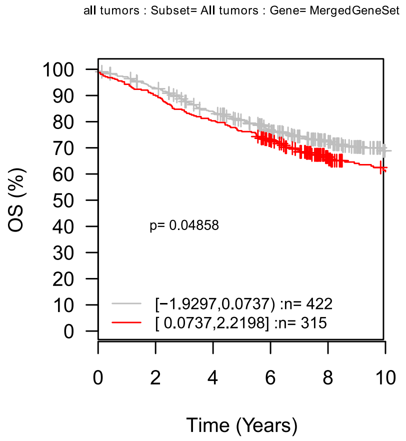


**MIMAT0000089 (hsa-miR-31-5p)***

GO:0012502, Induction of programmed cell death

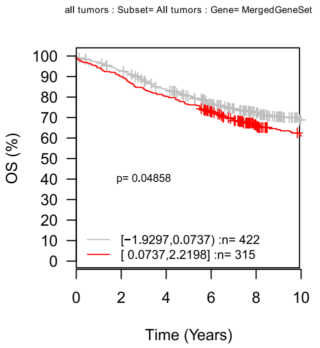


**MIMAT0000423 (hsa-miR-125b-5p)***

GO:0015630, Microtubule cytoskeleton

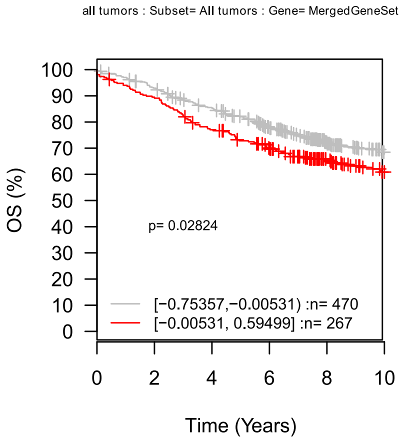

**Figure S14.** *Cont.*

**MIMAT0000423 (hsa-miR-125b-5p)****
GO:0050678, Regulation of epithelial cell proliferation

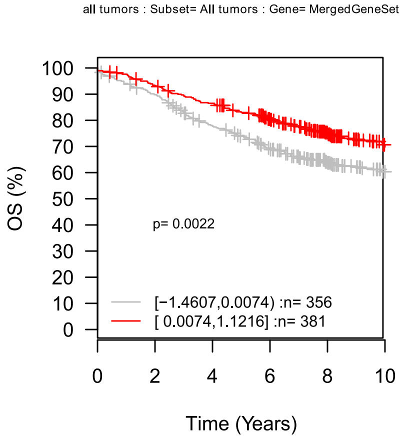


**MIMAT0000443 (hsa-miR-125a-5p)***
GO:0015630, Microtubule cytoskeleton

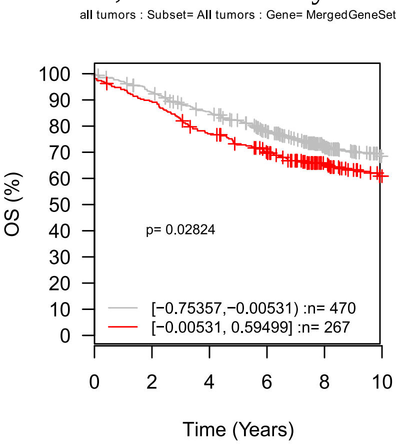


**MIMAT0000443 (hsa-miR-125a-5p)***
GO:0050678, Regulation of epithelial cell proliferation

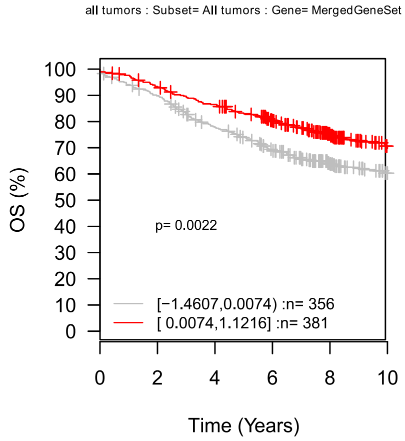


**MIMAT0002820 (hsa-miR-497-5p)***
GO:0007179, Transforming growth factor beta receptor signaling pathway

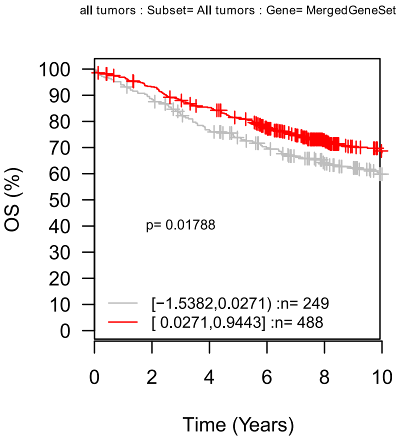


**MIMAT0002856 (hsa-miR-520d-3p)*****
GO:0015630, Microtubule cytoskeleton

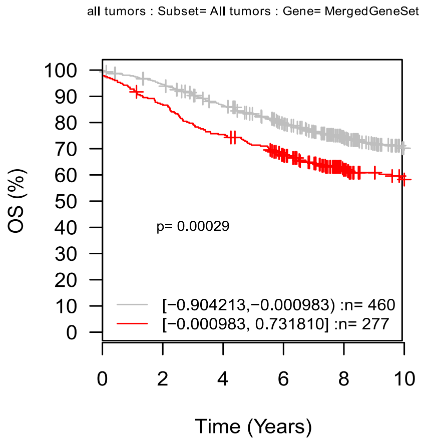


**MIMAT0002856 (hsa-miR-520d-3p)***
GO:0048011, Nerve growth factor receptor signaling pathway

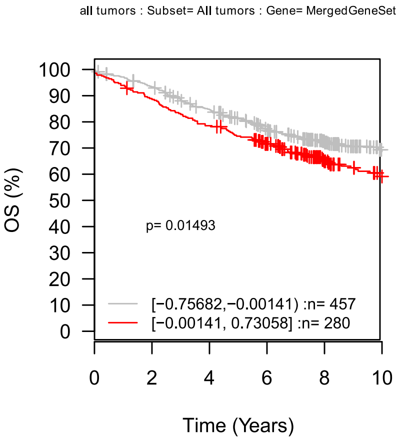

**Figure S14.** *Cont.*

**MIMAT0002856 (hsa-miR-520d-3p)*****

GO:0051988, Regulation of attachment of spindle microtubules to kinetochore

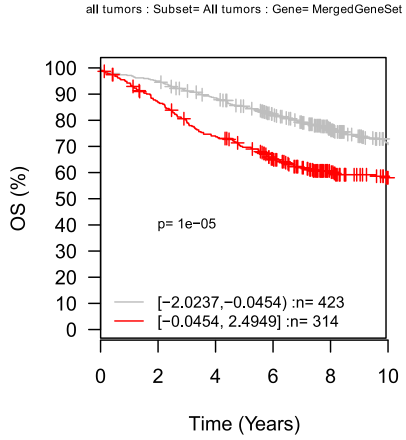


© 2013 by the authors; licensee MDPI, Basel, Switzerland. This article is an open access article distributed under the terms and conditions of the Creative Commons Attribution license (http://creativecommons.org/licenses/by/3.0/).
